# Cryptic speciation in arid mountains: An integrative revision of the *Pristurus rupestris* species complex (Squamata, Sphaerodactylidae) from Arabia based on morphological, genetic and genomic data, with the description of four new species

**DOI:** 10.1371/journal.pone.0315000

**Published:** 2025-02-24

**Authors:** Bernat Burriel-Carranza, Thore Koppetsch, Juliana Tabares, Adrián Talavera, Gabriel Mochales-Riaño, Maria Estarellas, Benjamin Wipfler, Johannes Els, Marc Simó-Riudalbas, Dean Adams, Saleh Al Saadi, Joan Garcia-Porta, Karin Tamar, Jiří Šmíd, Salvador Carranza

**Affiliations:** 1 Passeig Marítim de la Barceloneta, Institute of Evolutionary Biology (CSIC-Universitat Pompeu Fabra), Barcelona, Spain; 2 Museu de Ciències Naturals de Barcelona, Barcelona, Spain; 3 Natural History Museum, University of Oslo, Blindern, Oslo, Norway; 4 Zoological Research Museum Alexander Koenig, Leibniz Institute for the Analysis of Biodiversity Change, Bonn, Germany; 5 Breeding Centre for Endangered Arabian Wildlife, Environment and Protected Areas Authority, Sharjah, United Arab Emirates; 6 Department of Ecology, Evolution, and Organismal Biology, Iowa State University, Ames, Iowa, United States of America; 7 Environment Authority, Muscat, Oman; 8 Department of Biodiversity, Ecology and Evolution, Complutense University of Madrid, Madrid, Spain; 9 Department of Zoology, National Museum, , Prague, Czech Republic; 10 Department of Zoology, Faculty of Science, Charles University, Prague, Czech Republic; State Museum of Natural History, GERMANY

## Abstract

In the arid landscapes of the Arabian Peninsula, high levels of cryptic diversity among reptiles, and especially in geckos, have recently been revealed. Mountain ranges within the peninsula were shown to contain the highest richness of reptile endemicity, serving as refugia to species less adapted to the hyper-arid conditions of the lowlands. With up to 19 endemic reptile species, the Hajar Mountains of southeastern Arabia are a clear example of this pattern. Owing to its old geological history, complex topography and geographic isolation from the rest of the peninsula, this mountain range rises as a hotspot of reptile biodiversity and endemicity in Arabia, and provides the perfect scenario to study the processes of evolution and diversification of reptiles in arid mountain ranges. In the present study we investigate the systematics of the *Pristurus rupestris* species complex, a group of geckos exhibiting cryptic morphological traits along with a remarkably deep evolutionary history. Initially considered a single species distributed throughout coastal Arabia, and with some scattered populations at the Horn of Africa, several recent studies have shown that *Pristurus rupestris* actually comprises a species complex restricted to the Hajar Mountains of southeastern Arabia. Here, we utilize an integrative approach assembling several morphological, genetic, genomic, and ecological datasets to resolve this long-standing systematic challenge. Results support the existence of four new cryptic *Pristurus* species in the Hajar Mountains with three new Oman endemics. While no unique diagnostic morphological characters were identified, some slight morphological differences occur between species, especially among high-elevation species relative to the rest. Despite the lack of clear morphological differentiation, extreme levels of genetic variation were found between species with genetic distances of up to 24% in the *12S* mitochondrial marker, resulting from deep divergence times of up to 10 mya. Moreover, all species have been found in sympatry with at least another representative of the species complex and without any signs of apparent and ongoing gene flow among them. These findings yield profound implications for conservation efforts, as one of these newly described species presents an extremely restricted distribution (only known from a single locality and three individuals), requiring immediate attention for protection. Overall, this study sheds light on the hidden diversity within the *P*. *rupestris* species complex, emphasizing the importance of preserving biodiversity in the face of ongoing environmental changes, while highlighting, once again, the Hajar Mountains of southeastern Arabia as a cradle of reptile biodiversity.

## Introduction

Over the past two decades, considerable taxonomic efforts have been made to better understand the Arabian reptile fauna. This has led to the discovery and description of dozens of species endemic to the peninsula [1–16], reshaping our understanding of reptile species inhabiting arid environments. What was once considered an impoverished biome filled with homogeneous species with wide distributions has revealed to be a region teeming with hidden diversity. For instance, a recent review on Arabian squamates highlights the significance of mountain ranges in the peninsula as hotspots of reptile diversity and endemicity [17]. These mountains are dominated by local, sometimes ancient, radiations of closely related and narrow-ranging species [17]. In one of such hotspots, the Hajar Mountains of southeastern Arabia, a small but extremely abundant semaphore gecko of the genus *Pristurus* Rüppell, 1835, exemplifies one of these radiations: The *Pristurus rupestris* Blanford, 1874 species complex.

**Fig 1:**
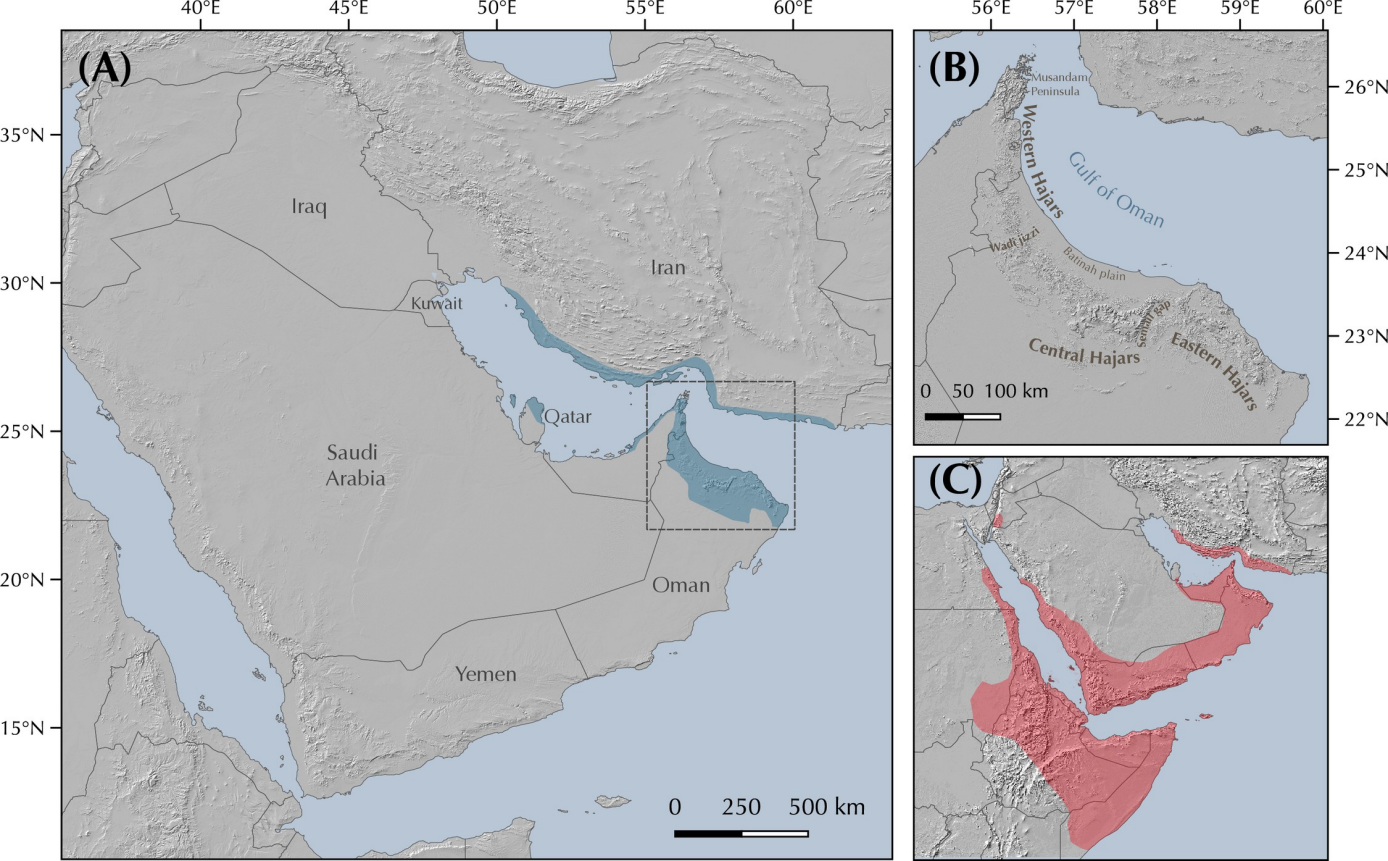
Geographic map showing **(A)** the *Pristurus rupestris* species complex distribution (blue). **(B)** a regional topographic map of the Hajar Mountains of southeastern Arabia with the main topographic features highlighted; **(C)** map showing the entire distribution of the *Pristurus* genus (red) with the exception of an isolated species in Mauritania (*P*. *adrarensis*).

The genus *Pristurus* is distributed across the southern coast of Iran, Arabia, the Socotra Archipelago, and the northeastern African coast, with a single geographically isolated species occurring in Mauritania [18–20] (Fig 1A). With 28 recognized species [21, 22], this genus thrives as the most diverse group of mostly diurnal geckos inhabiting the arid regions of the Old World. Like many sphaerodactylid geckos, all *Pristurus* have elongated fingers lacking adhesive digital pads, femoral and preanal pores, and cloacal sacs and bones [19, 23–25]. Most of them evolved distinctive adaptations to warm and photopic environments such as round pupils and relatively small eyes, but their most distinct characteristic, which gave rise to their common name ’semaphore geckos’, is their particular visual signaling using their body and tail; the latter often raised or curled during intraspecific signaling [19, 25, 26].

One particularly intricate group within the genus is the aforementioned *Pristurus rupestris* species complex, encompassing a variety of deep lineages with cryptic morphological traits [27]. Initially thought to be a single widespread taxon, “*P*. *rupestris”* was first recognized as polyphyletic through a taxonomic revision [18]. This led to its division into two clades: the Western Clade (which extends from coastal Southern Oman to Yemen, Western Saudi Arabia, and up to Jordan) temporarily named *Pristurus* sp 1 [18] and posteriorly assigned to *P*. *guweirensis* [22], and the Eastern Clade, which was restricted to the Hajar Mountains of southeastern Arabia and the coast of Iran, and maintained the nomen *P*. *rupestris*. Notably, subsequent molecular analyses revealed a cryptic species complex within the Arabian *P*. *rupestris*, characterized by up to 14 mitochondrial deep lineages with allopatric or parapatric distributions [27]. These findings were corroborated by a recent study on the endemic reptile fauna of the Hajar Mountains using genome-wide SNP data, with up to 12 nuclear deep lineages identified within the *P*. *rupestris* species complex [28].

In Arabia, the members of the *P*. *rupestris* species complex are endemic to the largest mountain range of southeastern Arabia, the Hajar Mountains, with scattered (and most probably introduced) populations outside the Hajar Mountains in the southeastern coast of Oman, on the northern United Arab Emirates (UAE) coast, from Sharjah to Abu Dhabi, and also in Qatar, Bahrain, and the eastern coast of Saudi Arabia. The Hajar Mountains have an old and complex geological history that dates back approximately 90 million years ago (mya) [29]. However, the current topography probably originated about 40 mya and was linked to the Arabian-Eurasian plate collision, with two secondary uplifts circa 15 and 6 mya that ended up fine tuning the current topography of this spectacular mountain range [29–32]. Nowadays, the Hajar Mountains are divided into three main mountain blocks: the Western, Central and Eastern Hajars (Fig 1B). The mountains reach up to 3,009 m above sea level (asl) in Jabal Shams, Jabal Akhdar, Central Hajars, but peaks above 2,000 m are found in all three mountain blocks. Therefore, they reach high enough elevations to significantly affect the local climate, representing the most climatically variable region in Oman and the UAE, with rainfall being considerably higher than their surrounding arid lowland regions to the South and West [26, 33, 34].

Due to its heterogeneous topography and geographic isolation from other mountain systems, the Hajar Mountains stand out as a hotspot of endemicity in Arabia, especially for reptiles [17, 26, 35]. With up to 19 described endemic species, the Hajar Mountains are home to a relatively high number of endemic squamates. Among them, we find one species of saw-scaled viper, *Echis omanensis* Babocsay, 2004; two lacertid species of the endemic genus *Omanosaura*, *O*. *cyanura* (Arnold, 1972) and *O*. *jayakari* (Boulenger, 1887); one species of agamid, *Pseudotrapelus jensvindumi* Melnikov, Ananjeva and Papenfuss, 2013; seven species of geckos of the genus *Asaccus*, *A*. *montanus* Gardner, 1994, *A*. *gardneri* Carranza, Simó-Riudalbas, Jayasinghe, Wilms and Els, 2016, *A*. *caudivolvulus* Arnold and Gardner, 1994, *A*. *margaritae* Carranza, Simó-Riudalbas, Jayasinghe, Wilms and Els, 2016, *A*. *platyrhynchus* Arnold and Gardner, 1994, *A*. *arnoldi* Simó-Riudalbas, Tarroso, Papenfuss, Al-Sariri and Carranza, 2018, and *A*. *gallagheri* Arnold, 1972; two species of geckos of the genus *Hemidactylus*, *H*. *luqueorum* Carranza and Arnold, 2012 and *H*. *hajarensis* Carranza and Arnold, 2012; two species of geckos of the genus *Pristurus*, *P*. *gallagheri* Arnold, 1986 and *P*. *celerrimus* Arnold, 1977; two species of geckos of the genus *Ptyodactylus*, *P*. *orlovi* Nazarov, Melnikov and Melnikova, 2013 and *P*. *ruusaljibalicus* Simó-Riudalbas, Metallinou, de Pous, Els, Jayasinghe, Péntek-Zakar, Wilms, Al-Saadi and Carranza, 2017; one species of gecko of the genus *Trachydactylus*, *T*. *hajarensis* (Arnold, 1980); and several candidate species assigned to the *P*. *rupestris* species complex [26–28, 33, 35].

Recent molecular studies have shown that cryptic species are not uncommon in this mountain range, as exemplified by both *Ptyodactylus* species [13], or by *Asaccus gallagheri* and *A*. *arnoldi* [14]. Moreover, analyses using genome-wide SNP data showed that the endemic reptile diversity is highly structured following the local topography of the mountain range, identifying up to 49 endemic deep lineages, usually found in allopatry or parapatry to other lineages of the same species [28]. The disparity between taxonomically described species diversity and the number of deep lineages identified provides a hint towards the levels of unrecognized, cryptic diversity still hidden within this mountain range. Such disparity was most apparent for *P*. *rupestris* geckos, where up to 12 deep lineages were identified [28]. *Pristurus rupestris* represents the second oldest and the most diverse group originating from a single colonization event in the Hajar Mountains, only comparable to the prolific species radiation of the second Hajar Mountain’s colonization of *Asaccus* (clade comprised by all *Asaccus* species mentioned above except for *A*. *montanus;* [28]). With respect to the latter, six species are currently recognized, while *P*. *rupestris* is still referred to as a single taxon.

However, discerning between the intertwined fine line of the Wallacean and Linnean shortfalls (that of our poor biogeographic and poor taxonomic knowledge respectively) when dealing with cryptic species constitutes a multifaceted endeavor. While the morphological uniformity of this complex conceals high levels of genetic diversity, the challenge still remains on where to set the threshold to discern intra- from interspecific variability. With the increasing availability of genome-wide data, several studies have raised concerns showing that Multispecies Coalescent (MSC) species delimitation methods tend to capture population splits rather than species-level divergence when using hundreds to thousands of SNPs [36–41]. Therefore, in the present work we implement an integrative approach analyzing morphological, genetic, genomic and ecological data to investigate in depth the nuances of cryptic speciation in this intricate species complex, aiming to resolve the systematics and diversification history of the *P*. *rupestris* species complex inhabiting the Hajar Mountains of Oman and the UAE. The results of our integrative analyses revealed four new species of *Pristurus* that are described herein, three of them endemic to Oman. Moreover, the findings on this taxonomic review are of paramount importance for effective conservation efforts since some of the herein described species exhibit restricted distributions (with one particular species restricted to a single locality), warranting immediate attention for protection. This study aims to contribute to a holistic understanding of the true diversity within the *Pristurus rupestris* species complex, and by extension, highlights the importance of balanced taxonomic assessments in the era of genomics and evolutionary insights.

## Materials and methods

### Ethics statement

No in vivo experiments were performed. Specimens were collected and manipulated with the authorization and under strict control and permission of the governments of Oman (Environment Authority) and the United Arab Emirates (Environment and Protected Areas Authority, Government of Sharjah), who approved the study. Specimens were captured and processed following the guidelines and protocols stated in the collecting permits and agreements obtained from the competent authorities of Oman and the United Arab Emirates (see references below). Members of the government supervised collecting activities. All efforts were made to minimize animal suffering. All the necessary collecting and export permits for this study in Oman were issued by the Nature Conservation Department of the Ministry of Environment and Climate Affairs, Oman (currently Environment Authority) (Refs: 08/2005; 16/2008; 38/2010; 12/2011; 13/ 2013; 21/2013; 37/2014; 601 31/2016; 6210/10/21) and the research in the United Arab Emirates was done under the supervision and permission of the Environment and Protected Areas Authority, Government of Sharjah. This research is not institutional. As a result of the characteristics of this study and the total control and compliance with the laws, regulations and procedures of this kind of biodiversity studies in Oman and the United Arab Emirates, it did not need the approval of an Institutional Animal Care and Use Committee (IACUC) or ethics committee.

### Data collection

In the present work, we assess the morphological, genetic, genomic, and geographic variability within the *Pristurus rupestris* species complex, aiming at resolving a more than one-decade old taxonomic challenge. To achieve this, we gathered or generated the following data: *i*) 11 morphological linear measurements from 150 specimens extracted from Garcia-Porta et al. 2017 [27]; *ii)* μ-computer-tomography (μ-CT) scans from four specimens representing the holotypes of the new species of *Pristurus* described herein; *iii*) one mitochondrial gene from 173 specimens (115 extracted from Garcia-Porta et al. 2017 [27]; 58 sequenced in the present study); *iv*) Four nuclear genes from up to 75 specimens extracted from Garcia-Porta et al., 2017 [27]; *v*) genome-wide ddRADseq data from 173 specimens (available from Dryad repository https://doi.org/10.5061/dryad.r7sqv9sj3) [28]; *vi*) Geographic data from 604 specimens [33, 34]. All specimens used in this study were collected or recorded between 2006 and 2016. Vouchers were preserved in 99% ethanol and stored at -20ºC. Tissues from specimens used in molecular analyses were tail tips stored in ethanol at -20ºC until DNA extraction.

### Morphological analyses

We characterized the morphology of each candidate species by analyzing 11 different measurements from 150 adult *Pristurus rupestris* (data obtained from Garcia-Porta et al. 2017) [27]. Measurements characterized body size by measuring snout-vent length (SVL; measured from the snout to the cloaca); head length by measuring from the snout to the anterior ear border (HL), head width, taken at the anterior ear border (HW) and head height, taken laterally at the anterior ear border (HH in the present study; head depth, HD, in [27]); body proportions by measuring the trunk length as the distance between the internal parts of the fore and hind limb insertion points (TrL in the present study; axilla to groin length, AGL, in [27]); the body width at the level of the scapular and pelvic girdles (ASG and APG respectively); the forelimb proportions by measuring the humerous length from the elbow to the insertion of the forelimb on the anterior part of body (LHu in the present study; brachium length, BL, in [27]), the ulna length, measured from the wrist to the elbow (LUn in the present study; ante-brachium length, AL, in [27]); the hindlimb proportions by measuring the femur length from the knee to the insertion of the hindlimb on the posterior side of body (LFe in the present study; thigh length, TL, in [27]), and the tibia length measured from the ankle to the knee (LTb in the present study; crus length, CL, in [27]).

As morphological differentiation is negligible among sexes in this species complex [27], we pooled all specimens for subsequent analyses without accounting for sex. Then, data were log10 transformed to improve normality and homoscedasticity. Body size effect was removed by extracting the residuals from regressing the data on SVL. We then determined the morphospace with a Principal Component Analysis (PCA; ‘geomorph v.4.0.4’; [42]). Because all linear measurements were in commensurate units and of similar scale, we did not standardize the data prior to computing the PCA (see [43, 44]), in contrast to Garcia-Porta et al. [27]. We quantified the relative amount of shape variation with a Procrustes ANOVA using shape as a response variable and specimen assignment to each species described below as the independent variable (package ‘geomorph v.4.0.4’; [42]). Significance was estimated via distributions generated from a resampling of 1,000 permutations (package ‘geomorph v.4.0.4’; [42]). Then, we generated pairwise statistics for each pair of putative species interrogating differences on shape means between candidate species (package ‘RRPP’; [45, 46]). To account for multiple testing of the data, pairwise comparisons were evaluated at the Bonferroni-adjusted alpha = 0.05 (for 10 pairwise comparisons).

Additionally, we obtained osteological information from each holotype specimen by micro-computed tomography (μ-CT) scanning. We scanned each specimen with a Skyscan 1173 μ-CT (Bruker, Billerica / USA) at the Museum Koenig in Bonn, Germany. The specimens were firmly fixed with foam in 70% EtOH filled tubes that were glued on a stub before scanning. Scans were performed with the following parameters: Voltage: 49 kV; Current: 133 μA; Rotation steps: 0.21º over 360º; Exposure time: 850 ms; Image pixel size: 18.1 μm; Frame averaging: 6; and a random movement: 15. All scans are part of the digital collection of the LIB and can be acquired by contacting T.K. or B.W. Subsequently, the scans were reconstructed with the software NRecon (Bruker, Billerica / USA). They were imported into Amira 6.1.2 (Thermofisher) where the skulls were separated from scales, the remaining skeletons, the filling material, and the tube. Subsequent volume rendering was performed in VG Studio 3.3 (Volume Graphics). Final images and plates were edited and arranged with Adobe CS6 (Adobe, San Jose / USA). Measurements of osteological elements were conducted by using the ImageJ software (National Institutes of Health, Bethesda, MD). Terminology of cranial osteological characters follows Lobon-Rovira & Bauer (2021) [47].

### Single locus analyses

We sequenced 58 specimens for the 12S rRNA (*12S*) mitochondrial gene and, together with 115 additional samples downloaded from GenBank, we assembled a dataset of 173 *Pristurus* specimens (Table 1) with the same specimen composition to the one in Burriel-Carranza et al. (2024) [28]. This way, we were able to compare both mitochondrial and nuclear phylogenetic histories and interrogate if there were any mito-nuclear discordances among taxa. DNA extraction was done following the protocol in MacManes (2013) [48] and PCR amplification conditions and primers used were the same as described in Metallinou et al. (2015) [49]. PCR products were purified and Sanger sequenced by Macrogen Inc. to obtain a fragment of approximately 400 bp of the mitochondrial gene *12S*. Sequences were aligned with Geneious 2021.1.1 (Biomatters Ltd.) and a Maximum Likelihood (ML) phylogeny was reconstructed in RAxML-ng v.1.0.2 [50] with a GTR+G model, a total of 100 starting trees (50 random and 50 parsimony) and 1,000 bootstrap replicates to estimate branch support. Uncorrected genetic distances (*p*-distances) were also inferred for the same dataset of the mitochondrial *12S* gene, using the package ‘ape v5.0’ [51] within the R environment.

**Table 1:** Table of all specimens used for the present study with the assignment of each specimen to its corresponding species described herein. Information regarding specimen assignment to genetic and genomic deep lineages, specimens used as type material, geographic location, elevation, 12S accession numbers, MorphoBank accession codes, as well as information on which individuals were included in each morphological, genetic, genomic and geographic dataset are provided. Genomic *dataset1* and *dataset2* include all specimens used for genomic analyses (Genomic column) except *P*. *flavipuntatus* specimens; Genomic *dataset3* includes all specimens used for genomic data; Specimens selected for *dataset4* are highlighted in bold in the Genomic column. Holotype (H) and paratype (P) museum codes refer to the following collections: IBE[X]: field series of S. Carranza housed at the Institute of Evolutionary Biology (CSIC-UPF); ZFMK[X]: Zoologisches Forschungsmuseum Alexander Koenig, Bonn, Germany; NHMOK[X]: the Naturhistorisk Museum, Oslo, Norway; MZB [X]: Museu de Ciències Naturals de Barcelona, Spain.

Species	Specimen Code	Candidate species in Garcia-Porta et al. (2017)	Candidate species in Burriel-Carranza et al. (2024)	Type material and Museum codes	Lat.	Lon.	Elev	12S accession code	Morphology	Nuclear genes	Genomic	MorphoBank code
*P*. *rupestris* sensu stricto	CN190	1	BFD1	-	23.075	57.606	1906	KY023454	yes		yes	-
*P*. *rupestris* sensu stricto	S6134	1	BFD1	-	22.923	57.680	601	KY023464	yes	*cmos*,*rag1*	yes	-
*P*. *rupestris* sensu stricto	S6120	1	BFD1	-	22.982	57.701	1164	KY023462	-		**yes**	-
*P*. *rupestris* sensu stricto	S7809	1	BFD1	-	23.091	57.734	1892	KY023470	yes		yes	-
*P*. *rupestris* sensu stricto	S6101	1	BFD1	-	23.080	57.613	1920	KY023461	-	*cmos*,*mc1r*,*rag1*,*rag2*	yes	-
*P*. *rupestris* sensu stricto	S7352	1	BFD1	-	22.974	57.700	990	KY023466	-		yes	-
*P*. *rupestris* sensu stricto	CN51	1	-	-	23.075	57.610	1800	-	-		-	-
*P*. *rupestris* sensu stricto	CN6	1	-	-	23.072	57.603	1877	-	-		-	-
*P*. *rupestris* sensu stricto	CN11	1	-	-	23.095	57.723	2008	-	-		-	-
*P*. *rupestris* sensu stricto	CN4268	1	-	-	23.093	57.723	2008	-	-		-	M849627–M849629
*P*. *rupestris* sensu stricto	CN4018	1	-	-	22.926	57.667	584	-	yes		-	-
*P*. *rupestris* sensu stricto	CN4246	1	-	-	22.926	57.667	584	-	yes		-	-
*P*. *rupestris* sensu stricto	S7652b	1	-	-	22.933	57.669	671	-	-		-	-
*P*. *rupestris* sensu stricto	S6121	1	-	-	22.971	57.696	967	-	-		-	-
*P*. *rupestris* sensu stricto	S6067	1	-	-	22.996	57.704	1472	-	-		-	-
*P*. *rupestris* sensu stricto	S6076	1	-	-	23.009	57.698	1814	-	-		-	M849612–M849614
*P*. *rupestris* sensu stricto	S6064	1	-	-	22.974	57.700	990	-	yes	*cmos*,*mc1r*,*rag1*,*rag2*	-	-
*P*. *rupestris* sensu stricto	S7792	1	-	-	22.974	57.700	990	-	-		-	-
*P*. *rupestris* sensu stricto	S7291	1	-	-	22.974	57.700	990	-	-		-	-
*P*. *rupestris* sensu stricto	S6035	1	-	-	23.080	57.613	1920	-	-	*cmos*,*mc1r*,*rag1*,*rag2*	-	-
*P*. *rupestris* sensu stricto	S7802	1	-	-	22.974	57.700	990	-	-		-	-
*P*. *rupestris* sensu stricto	CN7227	2	BFD1	-	23.163	57.033	1748	PQ653629	-		yes	-
*P*. *rupestris* sensu stricto	CN4055	2	BFD1	-	23.031	57.324	615	KY023475	-		yes	-
*P*. *rupestris* sensu stricto	S7239	2	BFD1	-	23.100	57.119	1001	KY023478	yes	*cmos*,*mc1r*,*rag1*,*rag2*	yes	M849616–M849619
*P*. *rupestris* sensu stricto	S7285	2	BFD1	-	23.119	57.096	991	KY023482	-		yes	-
*P*. *rupestris* sensu stricto	S7489	2	BFD1	-	23.125	57.404	1495	KY023491	yes		yes	-
*P*. *rupestris* sensu stricto	S7745	2	BFD1	-	23.080	57.422	956	KY023496	-		yes	-
*P*. *rupestris* sensu stricto	S7896	2	BFD1	-	23.081	57.350	682	KY023498	-		yes	-
*P*. *rupestris* sensu stricto	S7477	2	BFD1	-	23.081	57.350	682	KY023490	-		**yes**	-
*P*. *rupestris* sensu stricto	CN7291	2	-	-	23.151	57.029	2068	-	-		-	-
*P*. *rupestris* sensu stricto	CN4020	2	-	-	23.155	57.031	2068	-	-		-	-
*P*. *rupestris* sensu stricto	CN4170	2	-	-	23.155	57.031	2068	-	-		-	-
*P*. *rupestris* sensu stricto	CN172	2	-	-	23.161	57.033	1748	-	-		-	-
*P*. *rupestris* sensu stricto	CN7287	2	-	-	23.157	57.034	1816	-	-		-	-
*P*. *rupestris* sensu stricto	CN7207	2	-	-	23.158	57.034	1816	-	-		-	-
*P*. *rupestris* sensu stricto	CN7004	2	-	-	23.174	57.035	1395	-	-		-	-
*P*. *rupestris* sensu stricto	CN7170	2	-	-	23.175	57.035	1012	-	-		-	-
*P*. *rupestris* sensu stricto	CN3581	2	-	-	22.948	57.285	539	-	-		-	-
*P*. *rupestris* sensu stricto	CN81	2	-	-	23.194	57.202	1951	-	-		-	-
*P*. *rupestris* sensu stricto	CN6975	2	-	-	23.031	57.324	615	-	yes		-	-
*P*. *rupestris* sensu stricto	CN4237	2	-	-	23.031	57.324	615	-	yes		-	M849631–M849634
*P*. *rupestris* sensu stricto	AO6	2	-	-	23.056	57.467	704	-	-		-	-
*P*. *rupestris* sensu stricto	AO7	2	-	-	23.056	57.467	704	-	-		-	-
*P*. *rupestris* sensu stricto	AO8	2	-	-	23.056	57.467	704	-	-		-	-
*P*. *rupestris* sensu stricto	AO65	2	-	-	23.140	57.410	1668	-	-		-	-
*P*. *rupestris* sensu stricto	S7702	2	-	-	23.035	57.316	608	-	yes	*cmos*,*rag1*	-	-
*P*. *rupestris* sensu stricto	S7438	2	-	-	23.187	57.139	958	-	-	*cmos*,*mc1r*,*rag1*	-	-
*P*. *rupestris* sensu stricto	S7245	2	-	-	23.119	57.096	991	-	yes	*cmos*,*mc1r*,*rag1*,*rag2*	-	-
*P*. *rupestris* sensu stricto	S9020	2	-	-	23.104	57.355	1044	-	-		-	-
*P*. *rupestris* sensu stricto	S7622	2	-	-	23.104	57.355	1044	-	-		-	-
*P*. *rupestris* sensu stricto	S7573	2	-	-	23.125	57.404	1495	-	-		-	-
*P*. *rupestris* sensu stricto	S7273	2	-	-	23.132	57.462	2011	-	yes		-	-
*P*. *rupestris* sensu stricto	S7297	2	-	-	23.132	57.462	2011	-	-		-	-
*P*. *rupestris* sensu stricto	S7270	2	-	-	23.124	57.457	1743	-	-		-	-
*P*. *rupestris* sensu stricto	S7468	2	-	-	23.124	57.457	1743	-	-		-	-
*P*. *rupestris* sensu stricto	S7368	2	-	-	23.101	57.442	1425	-	-		-	-
*P*. *rupestris* sensu stricto	S7777	2	-	-	23.101	57.442	1425	-	-		-	-
*P*. *rupestris* sensu stricto	S7743	2	-	-	23.080	57.422	956	-	-		-	-
*P*. *rupestris* sensu stricto	S7303	2	-	-	23.119	57.096	991	-	-		-	-
*P*. *rupestris* sensu stricto	S7463	2	-	-	23.187	57.139	958	-	-		-	-
*P*. *rupestris* sensu stricto	S7901	2	-	-	23.101	57.442	1425	-	-		-	-
*P*. *rupestris* sensu stricto	S7408	2	-	-	23.187	57.139	958	-	-		-	M849621–M849625
*P*. *rupestris* sensu stricto	CN80	3	BFD2	-	23.653	57.879	21	KY023522	-		yes	-
*P*. *rupestris* sensu stricto	CN663	3	BFD2	-	23.034	58.912	818	PQ653630	-		yes	-
*P*. *rupestris* sensu stricto	CN3582	3	BFD2	-	23.084	58.941	217	PQ653639	-		yes	M849574–M849578
*P*. *rupestris* sensu stricto	CN3498	3	BFD2	-	23.034	58.992	203	PQ653640	-		yes	M849565–M849567
*P*. *rupestris* sensu stricto	CN5853	3	BFD2	-	22.936	59.133	363	PQ653637	-		yes	M849580–M849584
*P*. *rupestris* sensu stricto	CN4296	3	BFD2	-	22.936	59.133	363	PQ653638	-		**yes**	M849569–M849572
*P*. *rupestris* sensu stricto	CN3584	3	BFD2	-	22.539	59.367	126	PQ653633	-		yes	-
*P*. *rupestris* sensu stricto	CN5850	3	BFD2	-	22.658	59.224	878	PQ653635	-		yes	-
*P*. *rupestris* sensu stricto	CN4046	3	BFD2	-	22.647	59.271	529	PQ653632	-		**yes**	M849560–M849563
*P*. *rupestris* sensu stricto	CN3488	3	BFD2	-	22.610	59.290	430	PQ653634	-		yes	-
*P*. *rupestris* sensu stricto	CN91	3	BFD2	-	23.787	57.800	12	PQ653636	-		yes	-
*P*. *rupestris* sensu stricto	CN4181	3	BFD2	-	22.866	59.204	261	PQ653631	-		yes	-
*P*. *rupestris* sensu stricto	CN8983	3	BFD2	-	23.132	58.619	1682	KY023528	-		yes	-
*P*. *rupestris* sensu stricto	CN10299	3	BFD2	-	23.995	57.089	0	PQ653641	-		yes	-
*P*. *rupestris* sensu stricto	CN10294	3	BFD2	-	23.995	57.089	0	PQ653642	-		yes	-
*P*. *rupestris* sensu stricto	CN10288	3	BFD2	-	23.853	57.358	0	PQ653643	-		**yes**	-
*P*. *rupestris* sensu stricto	S1762	3	BFD2	-	23.512	58.645	18	KY023534	-		yes	-
*P*. *rupestris* sensu stricto	S7456	3	BFD2	-	23.086	59.047	7	KY023576	-		yes	-
*P*. *rupestris* sensu stricto	S7803	3	BFD2	-	23.451	57.865	256	KY023605	-		yes	-
*P*. *rupestris* sensu stricto	S7256	3	BFD2	-	23.589	58.165	56	KY023561	-		yes	-
*P*. *rupestris* sensu stricto	S7818	3	BFD2	-	23.340	58.625	235	KY023606	-		yes	-
*P*. *rupestris* sensu stricto	S7310	3	BFD2	-	22.539	59.368	136	KY023563	-	*cmos*,*mc1r*,*rag1*,*rag2*	yes	-
*P*. *rupestris* sensu stricto	S7545	3	BFD2	-	22.791	59.229	142	KY023588	-		yes	-
*P*. *rupestris* sensu stricto	S7527	3	BFD2	-	22.824	59.008	1365	KY023583	-		yes	-
*P*. *rupestris* sensu stricto	S7525	3	BFD2	-	23.165	58.385	655	KY023582	yes		yes	M849606–M849610
*P*. *rupestris* sensu stricto	S7787	3	BFD2	-	23.340	58.625	235	KY023604	-		yes	-
*P*. *rupestris* sensu stricto	S9016	3	BFD2	-	22.896	59.160	372	KY023610	yes		yes	M849586–M849589
*P*. *rupestris* sensu stricto	S9000	3	BFD2	-	23.133	58.652	1688	KY023609	-		**yes**	-
*P*. *rupestris* sensu stricto	S7675	3	BFD2	-	23.589	58.165	56	KY023598	-		yes	M849601–M849604
*P*. *rupestris* sensu stricto	S6040	3	BFD2	-	23.589	58.165	56	KY023542	-		yes	M849591–M849593
*P*. *rupestris* sensu stricto	S7544	3	BFD2	-	22.896	59.144	511	KY023587	yes		yes	-
*P*. *rupestris* sensu stricto	CN68	3	-	-	23.664	57.901	17	-	-		-	-
*P*. *rupestris* sensu stricto	CN108	3	-	-	23.700	57.776	15	-	-		-	-
*P*. *rupestris* sensu stricto	CN646	3	-	-	23.085	58.941	217	-	-		-	-
*P*. *rupestris* sensu stricto	CN685	3	-	-	23.034	58.912	818	-	-		-	-
*P*. *rupestris* sensu stricto	CN728	3	-	-	23.069	59.012	24	-	-		-	-
*P*. *rupestris* sensu stricto	CN5820	3	-	-	23.085	58.867	151	-	-		-	-
*P*. *rupestris* sensu stricto	CN3600	3	-	-	23.085	58.867	151	-	-		-	-
*P*. *rupestris* sensu stricto	CN3735	3	-	-	23.056	58.960	144	-	-		-	-
*P*. *rupestris* sensu stricto	CN3427	3	-	-	23.067	59.013	24	-	-		-	-
*P*. *rupestris* sensu stricto	CN3708	3	-	-	22.954	59.168	33	-	-		-	-
*P*. *rupestris* sensu stricto	CN5848	3	-	-	22.957	59.169	33	-	-		-	-
*P*. *rupestris* sensu stricto	CN5825	3	-	-	22.539	59.364	190	-	-		-	-
*P*. *rupestris* sensu stricto	CN178	3	-	-	22.539	59.370	126	-	-		-	-
*P*. *rupestris* sensu stricto	CN3465	3	-	-	22.658	59.223	878	-	-		-	-
*P*. *rupestris* sensu stricto	CN4035	3	-	-	22.657	59.224	878	-	-		-	-
*P*. *rupestris* sensu stricto	CN4379	3	-	-	22.647	59.271	529	-	-		-	-
*P*. *rupestris* sensu stricto	CN153	3	-	-	22.610	59.290	430	-	-		-	-
*P*. *rupestris* sensu stricto	CN2599	3	-	-	23.481	58.504	134	-	-		-	-
*P*. *rupestris* sensu stricto	CN2607	3	-	-	23.481	58.504	134	-	-		-	-
*P*. *rupestris* sensu stricto	CN2821	3	-	-	23.855	58.070	12	-	-		-	-
*P*. *rupestris* sensu stricto	CN4373	3	-	-	23.855	58.070	12	-	-		-	-
*P*. *rupestris* sensu stricto	CN2806	3	-	-	23.855	58.070	12	-	-		-	-
*P*. *rupestris* sensu stricto	CN87	3	-	-	23.788	57.795	12	-	-		-	-
*P*. *rupestris* sensu stricto	CN5841	3	-	-	23.786	57.796	12	-	-		-	-
*P*. *rupestris* sensu stricto	CN186	3	-	-	23.359	58.666	244	-	-		-	-
*P*. *rupestris* sensu stricto	CN2820	3	-	-	23.220	58.886	26	-	-		-	-
*P*. *rupestris* sensu stricto	CN15	3	-	-	23.220	58.886	26	-	-		-	-
*P*. *rupestris* sensu stricto	CN4280	3	-	-	22.850	58.954	740	-	-		-	-
*P*. *rupestris* sensu stricto	CN4002	3	-	-	22.850	58.954	740	-	-		-	-
*P*. *rupestris* sensu stricto	CN7147	3	-	-	23.132	58.619	1682	-	yes		-	-
*P*. *rupestris* sensu stricto	CN3997	3	-	-	22.917	59.191	56	-	yes		-	-
*P*. *rupestris* sensu stricto	CN8218	3	-	-	22.907	59.177	102	-	-		-	-
*P*. *rupestris* sensu stricto	CN3705	3	-	-	22.909	59.171	128	-	yes		-	-
*P*. *rupestris* sensu stricto	CN3590	3	-	-	22.896	59.160	363	-	-		-	-
*P*. *rupestris* sensu stricto	CN3720	3	-	-	22.895	59.140	719	-	yes		-	-
*P*. *rupestris* sensu stricto	CN179	3	-	-	22.847	59.138	1325	-	yes		-	-
*P*. *rupestris* sensu stricto	CN4004	3	-	-	22.690	59.353	351	-	-		-	-
*P*. *rupestris* sensu stricto	CN7208	3	-	-	23.132	58.619	1682	-	-		-	-
*P*. *rupestris* sensu stricto	CN7282	3	-	-	23.132	58.619	1682	-	-		-	-
*P*. *rupestris* sensu stricto	CN6997	3	-	-	23.132	58.619	1682	-	-		-	-
*P*. *rupestris* sensu stricto	CN4052	3	-	-	23.132	58.619	1682	-	-		-	-
*P*. *rupestris* sensu stricto	CN8972	3	-	-	23.132	58.619	1682	-	yes		-	-
*P*. *rupestris* sensu stricto	CN7171	3	-	-	23.132	58.619	1682	-	-		-	-
*P*. *rupestris* sensu stricto	CN4175	3	-	-	23.132	58.619	1682	-	yes		-	-
*P*. *rupestris* sensu stricto	CN8956	3	-	-	23.132	58.619	1682	-	-		-	-
*P*. *rupestris* sensu stricto	CN176	3	-	-	23.132	58.619	1682	-	-		-	-
*P*. *rupestris* sensu stricto	CN98	3	-	-	23.132	58.619	1682	-	-		-	-
*P*. *rupestris* sensu stricto	CN212	3	-	-	23.132	58.619	1682	-	-		-	-
*P*. *rupestris* sensu stricto	CN8653	3	-	-	23.132	58.619	1682	-	-		-	-
*P*. *rupestris* sensu stricto	CN169	3	-	-	23.132	58.619	1682	-	-		-	-
*P*. *rupestris* sensu stricto	CN4044	3	-	-	23.132	58.619	1682	-	-		-	-
*P*. *rupestris* sensu stricto	CN4048	3	-	-	23.132	58.619	1682	-	-		-	-
*P*. *rupestris* sensu stricto	CN8046	3	-	-	23.132	58.619	1682	-	-		-	-
*P*. *rupestris* sensu stricto	AO5	3	-	-	23.605	58.360	23	-	-		-	-
*P*. *rupestris* sensu stricto	UAE54	3	-	-	22.916	58.877	444	-	-		-	-
*P*. *rupestris* sensu stricto	UAE55	3	-	-	22.916	58.877	444	-	-		-	-
*P*. *rupestris* sensu stricto	UAE58	3	-	-	23.108	58.563	405	-	yes		-	-
*P*. *rupestris* sensu stricto	UAE59	3	-	-	23.108	58.563	405	-	-		-	-
*P*. *rupestris* sensu stricto	S1673	3	-	-	23.460	58.587	69	-	yes		-	-
*P*. *rupestris* sensu stricto	S586	3	-	-	23.512	58.645	18	-	-		-	-
*P*. *rupestris* sensu stricto	S1773	3	-	-	23.512	58.645	18	-	yes		-	-
*P*. *rupestris* sensu stricto	S1799	3	-	-	23.512	58.645	18	-	yes		-	-
*P*. *rupestris* sensu stricto	S1689	3	-	-	22.811	59.250	24	-	yes		-	-
*P*. *rupestris* sensu stricto	S1797	3	-	-	22.811	59.250	24	-	-		-	-
*P*. *rupestris* sensu stricto	S1754	3	-	-	22.811	59.250	24	-	-		-	-
*P*. *rupestris* sensu stricto	S1691	3	-	-	22.811	59.250	24	-	-		-	-
*P*. *rupestris* sensu stricto	S7414	3	-	-	23.293	57.977	384	-	-		-	-
*P*. *rupestris* sensu stricto	S7446	3	-	-	23.786	57.796	12	-	-		-	-
*P*. *rupestris* sensu stricto	S6100	3	-	-	22.497	59.397	71	-	yes	*cmos*,*mc1r*,*rag1*,*rag2*	-	-
*P*. *rupestris* sensu stricto	S7667	3	-	-	22.512	59.341	224	-	-		-	-
*P*. *rupestris* sensu stricto	S7729	3	-	-	23.072	58.187	676	-	-		-	-
*P*. *rupestris* sensu stricto	S7422	3	-	-	23.514	57.853	161	-	-		-	-
*P*. *rupestris* sensu stricto	S7458	3	-	-	23.549	57.476	190	-	-		-	-
*P*. *rupestris* sensu stricto	S6115	3	-	-	23.745	57.732	7	-	-		-	-
*P*. *rupestris* sensu stricto	S7472	3	-	-	23.468	58.194	173	-	-		-	-
*P*. *rupestris* sensu stricto	S7214	3	-	-	22.692	59.356	273	-	-		-	-
*P*. *rupestris* sensu stricto	S7864	3	-	-	23.691	58.039	12	-	-		-	-
*P*. *rupestris* sensu stricto	S7451	3	-	-	23.788	57.783	12	-	-		-	-
*P*. *rupestris* sensu stricto	S7232	3	-	-	22.700	59.295	872	-	-		-	-
*P*. *rupestris* sensu stricto	S7418	3	-	-	23.310	57.996	395	-	-		-	-
*P*. *rupestris* sensu stricto	S6108	3	-	-	23.409	58.100	236	-	-		-	-
*P*. *rupestris* sensu stricto	S7709	3	-	-	23.616	58.585	15	-	yes		-	-
*P*. *rupestris* sensu stricto	S7680	3	-	-	23.590	58.408	21	-	-		-	-
*P*. *rupestris* sensu stricto	S7738	3	-	-	23.452	58.505	152	-	-		-	-
*P*. *rupestris* sensu stricto	S7430	3	-	-	23.755	57.581	28	-	-		-	-
*P*. *rupestris* sensu stricto	S7417	3	-	-	23.587	58.163	69	-	-		-	-
*P*. *rupestris* sensu stricto	S7579	3	-	-	23.555	58.187	60	-	-		-	-
*P*. *rupestris* sensu stricto	S7065	3	-	-	22.870	59.183	876	-	-	*cmos*,*mc1r*,*rag1*,*rag2*	-	-
*P*. *rupestris* sensu stricto	S7247	3	-	-	22.870	59.183	876	-	yes		-	-
*P*. *rupestris* sensu stricto	S7269	3	-	-	22.873	59.172	1083	-	-		-	-
*P*. *rupestris* sensu stricto	S7543	3	-	-	22.873	59.172	1083	-	-	*cmos*,*mc1r*,*rag1*,*rag2*	-	-
*P*. *rupestris* sensu stricto	S7569	3	-	-	22.885	59.131	1137	-	-		-	-
*P*. *rupestris* sensu stricto	S7473b	3	-	-	22.895	59.138	861	-	-		-	-
*P*. *rupestris* sensu stricto	S7634	3	-	-	22.896	59.144	511	-	-		-	-
*P*. *rupestris* sensu stricto	S7561	3	-	-	22.896	59.160	372	-	yes		-	-
*P*. *rupestris* sensu stricto	S9018	3	-	-	22.833	58.988	1005	-	yes	*cmos*,*mc1r*,*rag1*,*rag2*	-	-
*P*. *rupestris* sensu stricto	S7565	3	-	-	22.763	58.853	591	-	-	*cmos*,*mc1r*,*rag1*,*rag2*	-	-
*P*. *rupestris* sensu stricto	S7049	3	-	-	23.106	58.644	1667	-	-		-	-
*P*. *rupestris* sensu stricto	S7082	3	-	-	23.106	58.644	1667	-	-		-	-
*P*. *rupestris* sensu stricto	S7083	3	-	-	23.133	58.652	1688	-	-		-	-
*P*. *rupestris* sensu stricto	S7197	3	-	-	23.133	58.652	1688	-	-		-	-
*P*. *rupestris* sensu stricto	S7534	3	-	-	23.132	58.619	1683	-	yes	*cmos*,*mc1r*,*rag1*,*rag2*	-	M849595–M849599
*P*. *rupestris* sensu stricto	S7070	3	-	-	23.077	58.648	1199	-	-		-	-
*P*. *rupestris* sensu stricto	S7481	3	-	-	23.077	58.648	1199	-	-		-	-
*P*. *rupestris* sensu stricto	S7177	3	-	-	23.165	58.385	655	-	-		-	-
*P*. *rupestris* sensu stricto	S7351	3	-	-	22.757	59.094	1196	-	-		-	-
*P*. *rupestris* sensu stricto	S7749	3	-	-	23.755	57.581	28	-	-		-	-
*P*. *rupestris* sensu stricto	S7218	3	-	-	22.700	59.295	872	-	-		-	-
*P*. *rupestris* sensu stricto	S6135	3	-	-	23.086	59.047	7	-	-		-	-
*P*. *rupestris* sensu stricto	S6140	3	-	-	23.589	58.165	56	-	-		-	-
*P*. *rupestris* sensu stricto	S6131	3	-	-	23.589	58.165	56	-	-		-	-
*P*. *rupestris* sensu stricto	S7575	3	-	-	22.885	59.131	1137	-	-		-	-
*P*. *rupestris* sensu stricto	S7553	3	-	-	23.132	58.619	1683	-	yes	*cmos*,*mc1r*,*rag1*,*rag2*	-	-
*P*. *rupestris* sensu stricto	S7535	3	-	-	23.106	58.644	1667	-	-		-	-
*P*. *rupestris* sensu stricto	S7548	3	-	-	23.106	58.644	1667	-	-		-	-
*P*. *rupestris* sensu stricto	S7493	3	-	-	23.077	58.648	1199	-	-		-	-
*P*. *rupestris* sensu stricto	S7068	3	-	-	23.555	58.187	60	-	-		-	-
*P*. *rupestris* sensu stricto	S6039	3	-	-	23.589	58.165	56	-	-		-	-
*P*. *rupestris* sensu stricto	S5719	3	-	-	23.589	58.165	0	-	-		-	-
*P*. *rupestris* sensu stricto	S6038	3	-	-	23.589	58.165	56	-	-		-	-
*P*. *rupestris* sensu stricto	S7409	3	-	-	23.786	57.796	12	-	yes		-	-
*P*. *rupestris* sensu stricto	S7874	3	-	-	23.310	57.996	395	-	-		-	-
*P*. *rupestris* sensu stricto	S7416	3	-	-	23.786	57.796	12	-	-		-	-
*P*. *rupestris* sensu stricto	S7439	3	-	-	23.786	57.796	12	-	-		-	-
*P*. *rupestris* sensu stricto	S7398	3	-	-	23.293	57.977	384	-	-		-	-
*P*. *rupestris* sensu stricto	S6132	4.5	-	-	23.872	56.907	160	-	yes	*cmos*,*mc1r*,*rag1*,*rag2*	-	-
*P*. *rupestris* sensu stricto	S7462	4.5	-	-	23.497	56.899	785	-	yes	*cmos*,*mc1r*,*rag1*	-	-
*P*. *rupestris* sensu stricto	S7182	4.5	-	-	23.176	57.652	932	-	-	*cmos*,*mc1r*,*rag1*,*rag2*	-	-
*P*. *rupestris* sensu stricto	S7158	4.5	-	-	23.952	56.702	370	-	yes	*cmos*,*mc1r*,*rag1*,*rag2*	-	M849636–M849639
*P*. *rupestris* sensu stricto	S7742	4.5	-	-	23.771	56.393	785	-	-	*cmos*,*mc1r*,*rag1*,*rag2*	-	-
*P*. *rupestris* sensu stricto	S7249	4.5	-	-	23.199	57.364	1328	-	yes	*cmos*,*mc1r*,*rag1*,*rag2*	-	-
*P*. *rupestris* sensu stricto	S7831	4.5	-	-	23.213	57.204	1952	-	-		-	-
*P*. *rupestris* sensu stricto	CN375	4.5	BFD3	-	23.379	57.450	414	KY023619	-		yes	-
*P*. *rupestris* sensu stricto	CN7210	4.5	BFD3	-	23.276	57.200	2115	PQ653644	-		yes	-
*P*. *rupestris* sensu stricto	CN63	4.5	BFD3	-	23.192	57.199	1887	PQ653646	-		yes	M849656–M849658
*P*. *rupestris* sensu stricto	CN7121	4.5	BFD3	-	24.621	56.340	238	PQ653645	-		yes	-
*P*. *rupestris* sensu stricto	CN5829	4.5	BFD3	-	23.390	57.218	524	KY023620	yes		yes	-
*P*. *rupestris* sensu stricto	CN3424	4.5	BFD3	-	23.401	57.429	340	KY023617	yes		yes	-
*P*. *rupestris* sensu stricto	S7181	4.5	BFD3	-	23.242	57.542	1095	KY023625	-		yes	-
*P*. *rupestris* sensu stricto	S7861	4.5	BFD3	-	23.385	57.836	364	KY023661	-		yes	-
*P*. *rupestris* sensu stricto	S7450	4.5	BFD3	-	23.196	57.198	1887	KY023646	-		yes	-
*P*. *rupestris* sensu stricto	S7251	4.5	BFD3	-	23.177	57.410	1308	KY023633	yes	*cmos*,*mc1r*,*rag1*,*rag2*	yes	-
*P*. *rupestris* sensu stricto	S7266	4.5	BFD3	-	23.199	57.364	1328	KY023635	-		yes	-
*P*. *rupestris* sensu stricto	S7255	4.5	BFD3	-	23.244	57.369	727	KY023634	yes	*cmos*,*mc1r*,*rag1*,*rag2*	**yes**	-
*P*. *rupestris* sensu stricto	S7432	4.5	BFD3	-	23.307	57.696	486	KY023643	-		yes	-
*P*. *rupestris* sensu stricto	S7406	4.5	BFD3	-	23.381	57.823	329	KY023639	-		yes	M849652–M849654
*P*. *rupestris* sensu stricto	S7768	4.5	BFD3	-	23.307	57.696	486	KY023653	-		**yes**	-
*P*. *rupestris* sensu stricto	S9021	4.5	BFD3	-	23.342	57.313	422	KY023668	-		yes	-
*P*. *rupestris* sensu stricto	CN3999	4.5	-	-	23.444	57.879	295	-	-		-	-
*P*. *rupestris* sensu stricto	CN2630	4.5	-	-	23.446	57.881	295	-	-		-	-
*P*. *rupestris* sensu stricto	CN105	4.5	-	-	23.281	57.163	2098	-	-		-	-
*P*. *rupestris* sensu stricto	CN83	4.5	-	-	23.896	57.021	82	-	-		-	-
*P*. *rupestris* sensu stricto	CN41	4.5	-	-	23.898	57.023	82	-	-		-	-
*P*. *rupestris* sensu stricto	CN16	4.5	-	-	23.190	57.199	1731	-	-		-	-
*P*. *rupestris* sensu stricto	CN26	4.5	-	-	23.655	56.927	474	-	-		-	-
*P*. *rupestris* sensu stricto	CN71	4.5	-	-	23.233	57.173	1749	-	-		-	-
*P*. *rupestris* sensu stricto	CN38	4.5	-	-	23.232	57.175	1749	-	-		-	-
*P*. *rupestris* sensu stricto	CN102	4.5	-	-	23.232	57.175	1774	-	-		-	-
*P*. *rupestris* sensu stricto	CN174	4.5	-	-	23.423	57.167	600	-	-		-	-
*P*. *rupestris* sensu stricto	CN46	4.5	-	-	23.200	57.202	1967	-	-		-	-
*P*. *rupestris* sensu stricto	CN6994	4.5	-	-	23.259	57.218	2100	-	-		-	-
*P*. *rupestris* sensu stricto	CN32	4.5	-	-	23.401	57.429	340	-	yes		-	-
*P*. *rupestris* sensu stricto	CN3709	4.5	-	-	23.401	57.429	340	-	yes		-	-
*P*. *rupestris* sensu stricto	CN60	4.5	-	-	23.191	57.199	1731	-	-		-	-
*P*. *rupestris* sensu stricto	S7433	4.5	-	-	23.869	56.402	745	-	yes		-	-
*P*. *rupestris* sensu stricto	S7421	4.5	-	-	23.213	57.204	1952	-	-		-	-
*P*. *rupestris* sensu stricto	S7455	4.5	-	-	23.238	57.150	1451	-	-		-	-
*P*. *rupestris* sensu stricto	S6116	4.5	-	-	23.762	56.894	309	-	yes		-	-
*P*. *rupestris* sensu stricto	S7889	4.5	-	-	23.307	57.696	486	-	-		-	-
*P*. *rupestris* sensu stricto	S7403	4.5	-	-	23.381	57.823	329	-	-		-	-
*P*. *rupestris* sensu stricto	S7461	4.5	-	-	23.533	57.334	241	-	yes		-	-
*P*. *rupestris* sensu stricto	S7412	4.5	-	-	23.385	57.239	530	-	-		-	-
*P*. *rupestris* sensu stricto	S7838	4.5	-	-	23.592	56.555	562	-	-		-	-
*P*. *rupestris* sensu stricto	S7466	4.5	-	-	23.408	57.424	323	-	-		-	-
*P*. *rupestris* sensu stricto	S7447	4.5	-	-	23.435	57.328	634	-	-		-	-
*P*. *rupestris* sensu stricto	S7822	4.5	-	-	23.441	57.119	646	-	-		-	-
*P*. *rupestris* sensu stricto	S7209	4.5	-	-	23.147	57.746	1112	-	yes		-	-
*P*. *rupestris* sensu stricto	S7470	4.5	-	-	23.390	57.832	313	-	-		-	-
*P*. *rupestris* sensu stricto	S7212	4.5	-	-	23.183	57.416	1124	-	-		-	-
*P*. *rupestris* sensu stricto	S7296	4.5	-	-	23.194	57.395	953	-	-		-	-
*P*. *rupestris* sensu stricto	S7240	4.5	-	-	23.218	57.378	1130	-	-		-	-
*P*. *rupestris* sensu stricto	S6046	4.5	-	-	23.223	57.375	1034	-	-		-	-
*P*. *rupestris* sensu stricto	S7873	4.5	-	-	23.342	57.313	422	-	-		-	-
*P*. *rupestris* sensu stricto	S7425	4.5	-	-	23.446	57.880	295	-	-		-	-
*P*. *rupestris* sensu stricto	S7892	4.5	-	-	23.495	57.035	729	-	-		-	-
*P*. *rupestris* sensu stricto	S7400	4.5	-	-	23.408	57.424	323	-	-		-	-
*P*. *rupestris* sensu stricto	S7894	4.5	-	-	23.771	56.393	785	-	-		-	-
*P*. *rupestris* sensu stricto	S7828	4.5	-	-	23.408	57.424	323	-	-		-	-
*P*. *rupestris* sensu stricto	S7202	4.5	-	-	23.176	57.652	932	-	-		-	-
*P*. *rupestris* sensu stricto	S7900	4.5	-	-	23.390	57.832	313	-	-		-	-
*P*. *rupestris* sensu stricto	S7841	4.5	-	-	23.307	57.696	486	-	-		-	-
*P*. *rupestris* sensu stricto	S7842	4.5	-	-	23.446	57.880	295	-	-		-	M849641–M849644
*P*. *rupestris* sensu stricto	S7237	4.5	-	-	23.177	57.410	1308	-	-		-	-
*P*. *rupestris* sensu stricto	S7880	4.5	-	-	23.497	56.899	785	-	-		-	-
*P*. *rupestris* sensu stricto	S7856	4.5	-	-	23.497	56.899	785	-	-		-	-
*P*. *rupestris* sensu stricto	S7241	4.5	-	-	23.183	57.416	1124	-	-		-	-
*P*. *rupestris* sensu stricto	S7196	4.5	-	-	23.178	57.762	867	-	yes	*cmos*,*mc1r*,*rag1*,*rag2*	-	-
*P*. *rupestris* sensu stricto	S7161b	4.5	-	-	23.148	57.735	1293	-	-	*cmos*,*mc1r*,*rag1*,*rag2*	-	-
*P*. *rupestris* sensu stricto	S7191	4.5	-	-	23.147	57.746	1112	-	-	*cmos*,*mc1r*,*rag1*,*rag2*	-	-
*P*. *rupestris* sensu stricto	S7159b	4.5	-	-	23.148	57.735	1293	-	yes	*cmos*,*mc1r*,*rag1*,*rag2*	-	M849646–M849650
*P*. *ali* **sp. nov.**	CN3737	6	BFD4	-	22.706	59.249	1489	KY023676	yes		yes	-
*P*. *ali* **sp. nov.**	S7198	6	BFD4	-	22.703	59.262	1374	KY023689	yes		yes	-
*P*. *ali* **sp. nov.**	S7507	6	BFD4	-	22.718	59.216	1593	KY023695	yes		**yes**	-
*P*. *ali* **sp. nov.**	S7813	6	BFD4	-	22.717	59.236	1551	KY023697	-	*cmos*,*mc1r*,*rag1*,*rag2*	yes	-
*P*. *ali* **sp. nov.**	S7231	6	BFD4	-	22.711	59.141	1956	KY023693	yes	*cmos*,*mc1r*,*rag1*,*rag2*	yes	M849671–M849674
*P*. *ali* **sp. nov.**	CN7011	6	-	-	22.706	59.249	1489	-	-		-	-
*P*. *ali* **sp. nov.**	CN6980	6	-	-	22.706	59.249	1489	-	yes		-	-
*P*. *ali* **sp. nov.**	CN147	6	-	-	22.717	59.233	1136	-	-		-	-
*P*. *ali* **sp. nov.**	CN7215	6	-	-	22.717	59.233	1136	-	yes		-	-
*P*. *ali* **sp. nov.**	CN8060	6	-	-	22.717	59.233	1136	-	-		-	-
*P*. *ali* **sp. nov.**	CN7168	6	-	-	22.717	59.233	1136	-	yes		-	M849676–M849680
*P*. *ali* **sp. nov.**	CN7112	6	-	-	22.717	59.233	1136	-	yes		-	-
*P*. *ali* **sp. nov.**	CN7198	6	-	-	22.717	59.233	1136	-	-		-	-
*P*. *ali* **sp. nov.**	CN3580	6	-	-	22.711	59.157	1863	-	-		-	-
*P*. *ali* **sp. nov.**	CN7960	6	-	-	22.711	59.157	1863	-	yes		-	-
*P*. *ali* **sp. nov.**	CN7096	6	-	-	22.711	59.157	1863	-	yes		-	-
*P*. *ali* **sp. nov.**	CN7181	6	-	-	22.711	59.157	1863	-	yes		-	-
*P*. *ali* **sp. nov.**	S7201b	6	-	-	22.717	59.236	1551	-	yes		-	-
*P*. *ali* **sp. nov.**	S7259	6	-	-	22.718	59.216	1593	-	yes		-	-
*P*. *ali* **sp. nov.**	S7186b	6	-	-	22.711	59.141	1956	-	-		-	-
*P*. *ali* **sp. nov.**	S7203	6	-	-	22.717	59.236	1551	-	yes	*cmos*,*mc1r*,*rag1*,*rag2*	-	M849660–M849664
*P*. *ali* **sp. nov.**	S7216	6	-	-	22.703	59.262	1374	-	yes		-	-
*P*. *ali* **sp. nov.**	S7185	6	-	-	22.703	59.262	1374	-	yes	*cmos*,*mc1r*,*rag1*,*rag2*	-	M849666–M849669
*P*. *ali* **sp. nov.**	S7570	6	-	-	22.718	59.216	1593	-	yes		-	-
*P*. *ali* **sp. nov.**	CN4240	7	BFD4	P—IBECN4240	22.832	59.020	1691	PQ653628	-		yes	M849687–M849689
*P*. *ali* **sp. nov.**	CN3480	7	BFD4	-	22.866	59.104	1361	KY023700	yes		yes	-
*P*. *ali* **sp. nov.**	CN8286	7	BFD4	P—IBECN8286	22.710	59.140	2046	KY023715	yes		yes	M849715–M849719
*P*. *ali* **sp. nov.**	CN175	7	BFD4	-	22.710	59.140	2046	KY023699	yes		yes	-
*P*. *ali* **sp. nov.**	CN3701	7	BFD4	-	22.740	59.106	1369	KY023705	-		yes	-
*P*. *ali* **sp. nov.**	S7200	7	BFD4	-	22.867	59.176	919	KY023720	-	*cmos*,*mc1r*,*rag1*,*rag2*	yes	-
*P*. *ali* **sp. nov.**	S7264	7	BFD4	-	22.845	59.138	1335	KY023727	yes		yes	-
*P*. *ali* **sp. nov.**	S7563	7	BFD4	P—MZB 2024–0984	22.841	59.098	1559	KY023746	-		**yes**	M849695–M849698
*P*. *ali* **sp. nov.**	S7509	7	BFD4	P—IBES7509	22.826	59.086	1717	KY023732	-		yes	M849691–M849693
*P*. *ali* **sp. nov.**	S7510	7	BFD4	-	22.826	59.086	1717	KY023733	yes		yes	-
*P*. *ali* **sp. nov.**	S7539	7	BFD4	P—IBES7539	22.706	59.142	2045	KY023740	yes	*cmos*,*mc1r*,*rag1*,*rag2*	yes	M849700–M849703
*P*. *ali* **sp. nov.**	S7226	7	BFD4	P—NHMOK2653	22.711	59.141	1956	KY023724	-		**yes**	-
*P*. *ali* **sp. nov.**	S7193	7	BFD4	P—IBES7193	22.825	59.112	1533	KY023719	-		yes	M849683–M849685
*P*. *ali* **sp. nov.**	S7229	7	BFD4	-	22.711	59.141	1956	KY023726	-		yes	-
*P*. *ali* **sp. nov.**	S7228	7	BFD4	-	22.848	59.139	1307	KY023725	-		yes	-
*P*. *ali* **sp. nov.**	S7211	7	BFD4	P—IBES7211	22.711	59.141	1956	KY023721	-		yes	M849710–M849713
*P*. *ali* **sp. nov.**	S7547	7	BFD4	H—ZFMK104093	22.766	59.034	1327	KY023742	yes	*cmos*,*mc1r*,*rag1*,*rag2*	yes	-
*P*. *ali* **sp. nov.**	CN4299	7	-	-	22.832	59.019	1691	-	-		-	-
*P*. *ali* **sp. nov.**	CN4157	7	-	-	22.822	59.044	1710	-	-		-	-
*P*. *ali* **sp. nov.**	CN12	7	-	-	22.822	59.044	1710	-	-		-	-
*P*. *ali* **sp. nov.**	CN3698	7	-	-	22.816	59.073	1755	-	-		-	-
*P*. *ali* **sp. nov.**	CN4010	7	-	-	22.816	59.073	1755	-	-		-	-
*P*. *ali* **sp. nov.**	CN3587	7	-	-	22.866	59.104	1361	-	-		-	-
*P*. *ali* **sp. nov.**	CN7269	7	-	-	22.866	59.104	1361	-	yes		-	-
*P*. *ali* **sp. nov.**	CN8628	7	-	-	22.866	59.104	1361	-	-		-	-
*P*. *ali* **sp. nov.**	CN3520	7	-	-	22.866	59.104	1361	-	-		-	-
*P*. *ali* **sp. nov.**	CN127	7	-	-	22.847	59.138	1325	-	yes		-	-
*P*. *ali* **sp. nov.**	CN4057	7	-	-	22.710	59.140	2046	-	-		-	-
*P*. *ali* **sp. nov.**	CN3491	7	-	-	22.710	59.140	2046	-	yes		-	-
*P*. *ali* **sp. nov.**	CN3992	7	-	-	22.710	59.140	2046	-	-		-	-
*P*. *ali* **sp. nov.**	CN3507	7	-	-	22.710	59.140	2046	-	yes		-	-
*P*. *ali* **sp. nov.**	CN4161	7	-	-	22.710	59.140	2046	-	-		-	-
*P*. *ali* **sp. nov.**	CN4178	7	-	-	22.710	59.140	2046	-	-		-	-
*P*. *ali* **sp. nov.**	CN4188	7	-	-	22.710	59.140	2046	-	yes		-	-
*P*. *ali* **sp. nov.**	CN7212	7	-	-	22.710	59.140	2046	-	yes		-	-
*P*. *ali* **sp. nov.**	CN4165	7	-	-	22.710	59.140	2046	-	yes		-	-
*P*. *ali* **sp. nov.**	CN4241	7	-	-	22.710	59.140	2046	-	-		-	-
*P*. *ali* **sp. nov.**	CN8960	7	-	-	22.710	59.140	2046	-	-		-	-
*P*. *ali* **sp. nov.**	S7225	7	-	P—MZB 2024–0983	22.825	59.112	1533	-	-		-	M849705–M849708
*P*. *ali* **sp. nov.**	S7219	7	-	-	22.848	59.139	1307	-	-		-	-
*P*. *ali* **sp. nov.**	S7490	7	-	-	22.883	59.124	1372	-	-		-	-
*P*. *ali* **sp. nov.**	S7501	7	-	-	22.883	59.124	1372	-	-		-	-
*P*. *ali* **sp. nov.**	S7531	7	-	-	22.841	59.098	1559	-	yes		-	-
*P*. *ali* **sp. nov.**	S9017	7	-	-	22.820	59.064	1775	-	-		-	-
*P*. *ali* **sp. nov.**	S7511	7	-	-	22.766	59.034	1327	-	yes		-	-
*P*. *ali* **sp. nov.**	S7560	7	-	-	22.770	59.076	1440	-	yes	*cmos*,*mc1r*,*rag1*,*rag2*	-	-
*P*. *ali* **sp. nov.**	S7556	7	-	-	22.740	59.106	1364	-	yes		-	-
*P*. *ali* **sp. nov.**	S7673b	7	-	-	22.740	59.106	1364	-	-		-	-
*P*. *ali* **sp. nov.**	S7529	7	-	-	22.718	59.121	1608	-	yes		-	-
*P*. *ali* **sp. nov.**	S7476	7	-	-	22.706	59.142	2045	-	-		-	-
*P*. *ali* **sp. nov.**	S7756	7	-	-	22.711	59.141	1956	-	-		-	-
*P*. *ali* **sp. nov.**	S7549	7	-	-	22.820	59.064	1775	-	-		-	-
*P*. *ali* **sp. nov.**	S7572	7	-	-	22.826	59.086	1717	-	-		-	-
*P*. *ali* **sp. nov.**	S7538	7	-	-	22.820	59.064	1775	-	-		-	-
*P*. *ali* **sp. nov.**	S7479	7	-	-	22.718	59.121	1608	-	-		-	-
*P*. *ali* **sp. nov.**	S7566	7	-	-	22.883	59.124	1372	-	-		-	-
*P*. *ali* **sp. nov.**	S7532	7	-	-	22.706	59.142	2045	-	-		-	-
*P*. *ali* **sp. nov.**	S7188	7	-	-	22.711	59.141	1956	-	-		-	-
*P*. *ali* **sp. nov.**	S7542	7	-	-	22.826	59.086	1717	-	-		-	-
*P*. *ali* **sp. nov.**	S7537	7	-	-	22.740	59.106	1364	-	-		-	-
*P*. *assareen* **sp. nov.**	CN192	16	BFD12	P—MZB 2024–0989	23.132	58.619	1682	KY023451	-		**yes**	M849930–M849932
*P*. *assareen* **sp. nov.**	S7257	16	BFD12	P—IBES7257	23.132	58.619	1683	KY023453	yes	*cmos*,*mc1r*,*rag1*,*rag2*	**yes**	M849924–M849928
*P*. *assareen* **sp. nov.**	CN3704	16	-	H—ZFMK104095	23.132	58.619	1682	-	yes		-	-
*P*. *feulneri* **sp. nov.**	CN10298	10.11	BFD7	-	24.171	56.892	12	PQ653660	-		yes	-
*P*. *feulneri* **sp. nov.**	CN10289	10.11	BFD7	-	24.171	56.892	12	PQ653661	-		yes	-
*P*. *feulneri* **sp. nov.**	CN56	10.11	BFD7	-	23.318	57.592	437	KY023387	-		**yes**	-
*P*. *feulneri* **sp. nov.**	CN133	10.11	BFD7	-	23.441	57.661	301	KY023386	-	*cmos*,*mc1r*,*rag1*,*rag2*	yes	-
*P*. *feulneri* **sp. nov.**	CN3476	10.11	BFD7	-	23.546	56.284	366	PQ653655	-		yes	-
*P*. *feulneri* **sp. nov.**	CN151	10.11	BFD7	-	23.711	56.443	676	PQ653663	-		yes	-
*P*. *feulneri* **sp. nov.**	CN225	10.11	BFD7	-	24.122	56.619	192	PQ653662	-		yes	-
*P*. *feulneri* **sp. nov.**	CN5823	10.11	BFD7	-	24.635	56.293	424	PQ653656	-		**yes**	-
*P*. *feulneri* **sp. nov.**	CN4060	10.11	BFD7	-	24.636	56.294	424	PQ653658	-		yes	-
*P*. *feulneri* **sp. nov.**	CN3420	10.11	BFD7	-	24.620	56.340	238	PQ653659	-		yes	M849730–M849732
*P*. *feulneri* **sp. nov.**	CN4244	10.11	BFD7	-	23.506	56.492	465	KY023359	yes		**yes**	-
*P*. *feulneri* **sp. nov.**	CN8380	10.11	BFD7	-	25.797	56.241	0	PQ653657	-		yes	-
*P*. *feulneri* **sp. nov.**	UAE34	10.11	BFD7	-	24.451	56.289	490	KY023382	yes		yes	-
*P*. *feulneri* **sp. nov.**	S7659	10.11	BFD7	-	24.822	56.070	369	KY023375	-		yes	-
*P*. *feulneri* **sp. nov.**	S7207	10.11	BFD7	-	24.227	56.325	405	KY023366	-		**yes**	-
*P*. *feulneri* **sp. nov.**	S7220	10.11	BFD7	-	24.408	55.997	450	KY023368	-		yes	-
*P*. *feulneri* **sp. nov.**	S7167b	10.11	BFD7	-	24.667	55.965	402	KY023364	yes	*cmos*,*mc1r*,*rag1*,*rag2*	yes	M849725–M849728
*P*. *feulneri* **sp. nov.**	S7424	10.11	BFD7	-	23.542	56.302	370	KY023372	-	*cmos*,*mc1r*,*rag1*,*rag2*	yes	-
*P*. *feulneri* **sp. nov.**	S7882	10.11	BFD7	-	23.388	57.659	331	KY023393	yes	*cmos*,*mc1r*,*rag1*,*rag2*	yes	M849721–M849723
*P*. *feulneri* **sp. nov.**	S7233	10.11	BFD7	-	24.302	56.127	652	KY023369	yes		yes	-
*P*. *feulneri* **sp. nov.**	S7166	10.11	-	-	24.302	56.127	652	-	yes	*mc1r*,*rag1*	-	-
*P*. *feulneri* **sp. nov.**	S7415	10.11	-	-	24.361	56.747	12	-	yes	*cmos*,*mc1r*,*rag1*,*rag2*	-	-
*P*. *feulneri* **sp. nov.**	CN69	10.11	-	-	23.373	57.666	339	-	-		-	-
*P*. *feulneri* **sp. nov.**	CN116	10.11	-	-	23.386	57.661	331	-	-		-	-
*P*. *feulneri* **sp. nov.**	CN99	10.11	-	-	23.466	57.672	201	-	-		-	-
*P*. *feulneri* **sp. nov.**	CN58	10.11	-	-	23.448	57.661	232	-	-		-	-
*P*. *feulneri* **sp. nov.**	CN111	10.11	-	-	23.417	57.669	323	-	-		-	-
*P*. *feulneri* **sp. nov.**	CN3503	10.11	-	-	23.390	57.660	331	-	-		-	-
*P*. *feulneri* **sp. nov.**	CN148	10.11	-	-	24.449	56.316	442	-	-		-	-
*P*. *feulneri* **sp. nov.**	CN3192	10.11	-	-	24.449	56.316	442	-	-		-	-
*P*. *feulneri* **sp. nov.**	CN8693	10.11	-	-	25.672	56.214	0	-	-		-	-
*P*. *feulneri* **sp. nov.**	CN74	10.11	-	-	23.541	56.301	359	-	-		-	-
*P*. *feulneri* **sp. nov.**	CN8298	10.11	-	-	23.533	56.318	375	-	yes		-	-
*P*. *feulneri* **sp. nov.**	SPM002894	10.11	-	-	25.281	56.186	460	-	-		-	-
*P*. *feulneri* **sp. nov.**	SPM002883	10.11	-	-	24.686	56.158	24	-	-		-	-
*P*. *feulneri* **sp. nov.**	SPM001302	10.11	-	-	25.208	56.327	0	-	-		-	-
*P*. *feulneri* **sp. nov.**	UAE31	10.11	-	-	24.515	56.462	104	-	yes		-	-
*P*. *feulneri* **sp. nov.**	UAE40	10.11	-	-	25.226	56.316	127	-	yes		-	-
*P*. *feulneri* **sp. nov.**	UAE23	10.11	-	-	25.417	56.168	428	-	-		-	-
*P*. *feulneri* **sp. nov.**	UAE24	10.11	-	-	25.417	56.168	428	-	-		-	-
*P*. *feulneri* **sp. nov.**	S7184b	10.11	-	-	24.287	56.098	577	-	-		-	-
*P*. *feulneri* **sp. nov.**	S7215	10.11	-	-	24.580	56.050	546	-	-		-	-
*P*. *feulneri* **sp. nov.**	S7426	10.11	-	-	23.506	56.464	468	-	-		-	-
*P*. *feulneri* **sp. nov.**	S7420	10.11	-	-	23.542	56.302	370	-	-		-	-
*P*. *feulneri* **sp. nov.**	S7155b	10.11	-	-	24.100	56.476	454	-	-		-	-
*P*. *feulneri* **sp. nov.**	S7443	10.11	-	-	24.361	56.747	12	-	-		-	-
*P*. *feulneri* **sp. nov.**	S7401	10.11	-	-	23.388	57.659	331	-	-	*mc1r*,*rag1*	-	-
*P*. *feulneri* **sp. nov.**	S7407	10.11	-	-	23.388	57.659	331	-	yes	*mc1r*,*rag1*,*rag2*	-	-
*P*. *feulneri* **sp. nov.**	CN4293	12	BFD8	-	23.231	57.900	460	PQ653652	-		yes	-
*P*. *feulneri* **sp. nov.**	CN114	12	BFD8	-	23.232	57.900	460	PQ653654	-		**yes**	M849765–M849767
*P*. *feulneri* **sp. nov.**	CN3562	12	BFD8	-	23.233	57.903	462	PQ653653	-		yes	M849744–M849748
*P*. *feulneri* **sp. nov.**	CN39	12	BFD8	-	23.175	57.853	565	KY023394	yes		yes	M849734–M849738
*P*. *feulneri* **sp. nov.**	S6060	12	BFD8	-	23.140	57.844	576	KY023396	yes	*cmos*,*mc1r*,*rag1*,*rag2*	**yes**	M849740–M849742
*P*. *feulneri* **sp. nov.**	CN4329	12	-	-	23.232	57.900	460	-	-		-	M849769–M849772
*P*. *feulneri* **sp. nov.**	CN7151	12	-	-	23.233	57.903	462	-	-		-	-
*P*. *feulneri* **sp. nov.**	CN4171	12	-	-	23.175	57.853	565	-	yes		-	M849756–M849759
*P*. *feulneri* **sp. nov.**	S7452	12	-	-	23.140	57.844	576	-	yes	*cmos*,*mc1r*,*rag1*	-	M849774–M849776
*P*. *feulneri* **sp. nov.**	S7730	12	-	-	23.175	57.853	543	-	yes	*cmos*,*mc1r*,*rag1*,*rag2*	-	M849750–M849754
*P*. *feulneri* **sp. nov.**	S7120	12	-	-	23.170	57.850	668	-	yes	*cmos*,*mc1r*,*rag1*,*rag2*	-	-
*P*. *feulneri* **sp. nov.**	S7475	12	-	-	23.140	57.844	576	-	yes		-	M849761–M849763
*P*. *feulneri* **sp. nov.**	S6102	13	BFD9	H—ZFMK104094	22.718	58.150	489	KY023407	yes	*cmos*,*mc1r*,*rag1*,*rag2*	yes	-
*P*. *feulneri* **sp. nov.**	S6123	13	BFD9	-	22.969	58.293	784	KY023408	yes		yes	-
*P*. *feulneri* **sp. nov.**	S7533	13	BFD9	P—IBES7533	23.143	58.424	555	KY023410	yes	*cmos*,*mc1r*,*rag1*,*rag2*	**yes**	M849793–M849796
*P*. *feulneri* **sp. nov.**	S7551	13	BFD9	P—NHMOK2655	23.143	58.424	555	KY023411	yes	*cmos*,*mc1r*,*rag1*,*rag2*	**yes**	-
*P*. *feulneri* **sp. nov.**	S7526	13	BFD9	-	23.143	58.424	555	KY023409	yes	*cmos*,*mc1r*,*rag1*,*rag2*	yes	-
*P*. *feulneri* **sp. nov.**	CN4040	13	-	-	23.143	58.424	549	-	-		-	-
*P*. *feulneri* **sp. nov.**	CN7213	13	-	P—MZB 2024–0985	23.143	58.424	549	-	-		-	M849789–M849791
*P*. *feulneri* **sp. nov.**	CN7239	13	-	P—IBECN7239	23.143	58.424	549	-	yes		-	M849778–M849783
*P*. *feulneri* **sp. nov.**	CN7939	13	-	P—MZB 2024–0986	23.143	58.424	549	-	-		-	M849785–M849787
*P*. *feulneri* **sp. nov.**	CN3489	13	-	-	23.143	58.424	549	-	yes		-	-
*P*. *feulneri* **sp. nov.**	CN3699	13	-	P—IBECN3699	23.143	58.424	549	-	yes		-	M849798–M849800
*P*. *feulneri* **sp. nov.**	S7599	13	-	-	23.143	58.424	555	-	-		-	-
*P*. *feulneri* **sp. nov.**	S7528	14	BFD10	-	23.149	57.463	2222	KY023429	yes	*cmos*,*mc1r*,*rag1*,*rag2*	yes	M849802–M849804
*P*. *feulneri* **sp. nov.**	S7736	14	BFD10	-	23.080	57.614	1920	KY023431	yes	*cmos*,*mc1r*,*rag1*,*rag2*	yes	M849806–M849810
*P*. *feulneri* **sp. nov.**	S6083	14	BFD10	-	23.069	57.641	1966	KY023420	-		**yes**	-
*P*. *feulneri* **sp. nov.**	CN4298	14	-	-	23.080	57.060	1580	-	-		-	-
*P*. *feulneri* **sp. nov.**	CN7091	14	-	-	23.072	57.634	1966	-	-		-	-
*P*. *feulneri* **sp. nov.**	CN4217	14	-	-	23.087	57.676	2011	-	-		-	M849818–M849820
*P*. *feulneri* **sp. nov.**	CN4174	14	-	-	23.079	57.670	1987	-	yes		-	-
*P*. *feulneri* **sp. nov.**	CN7200	14	-	-	23.079	57.670	1987	-	yes		-	-
*P*. *feulneri* **sp. nov.**	AO30	14	-	-	23.078	57.606	1925	-	-		-	-
*P*. *feulneri* **sp. nov.**	AO48	14	-	-	23.123	57.616	2298	-	-		-	-
*P*. *feulneri* **sp. nov.**	AO55	14	-	-	23.078	57.606	1925	-	-		-	-
*P*. *feulneri* **sp. nov.**	S7718	14	-	-	23.089	57.682	2011	-	-		-	-
*P*. *feulneri* **sp. nov.**	S6093	14	-	-	23.030	57.702	1988	-	-		-	-
*P*. *feulneri* **sp. nov.**	S6089	14	-	-	23.069	57.641	1966	-	-		-	M849812–M849816
*P*. *feulneri* **sp. nov.**	S7903	14	-	-	23.089	57.682	2011	-	-		-	-
*P*. *feulneri* **sp. nov.**	S6079	14	-	-	23.074	57.667	1992	-	-		-	-
*P*. *feulneri* **sp. nov.**	S7312	14	-	-	23.113	57.660	2258	-	yes		-	-
*P*. *feulneri* **sp. nov.**	S7284	14	-	-	22.982	57.701	1164	-	-		-	-
*P*. *feulneri* **sp. nov.**	S6087	14	-	-	23.069	57.641	1966	-	-		-	-
*P*. *feulneri* **sp. nov.**	S7288	14	-	-	23.149	57.463	2222	-	-		-	-
*P*. *feulneri* **sp. nov.**	S7294	14	-	-	23.149	57.463	2222	-	-		-	-
*P*. *feulneri* **sp. nov.**	S6036	14	-	-	23.080	57.613	1920	-	-	*cmos*,*mc1r*,*rag1*,*rag2*	-	-
*P*. *feulneri* **sp. nov.**	S6088	14	-	-	23.080	57.614	1920	-	-	*cmos*,*mc1r*,*rag1*,*rag2*	-	-
*P*. *feulneri* **sp. nov.**	CN4219	15	BFD11	-	22.437	59.263	386	PQ653647	-		yes	-
*P*. *feulneri* **sp. nov.**	CN4287	15	BFD11	-	22.480	59.283	565	PQ653649	-		yes	M849851–M849853
*P*. *feulneri* **sp. nov.**	CN4290	15	BFD11	-	22.479	59.284	425	PQ653648	-		**yes**	M849837–M849839
*P*. *feulneri* **sp. nov.**	UAE45	15	BFD11	-	22.620	59.093	674	KY023449	yes	*cmos*,*mc1r*	yes	-
*P*. *feulneri* **sp. nov.**	S7833	15	BFD11	-	22.572	59.094	600	KY023445	-		yes	-
*P*. *feulneri* **sp. nov.**	S7258	15	BFD11	-	22.766	59.034	1327	KY023441	yes	*cmos*,*mc1r*,*rag1*,*rag2*	yes	M849846–M849849
*P*. *feulneri* **sp. nov.**	S7546	15	BFD11	-	22.770	59.076	1440	KY023442	yes	*cmos*,*mc1r*,*rag1*,*rag2*	yes	-
*P*. *feulneri* **sp. nov.**	S7739	15	BFD11	-	22.602	59.083	617	KY023444	-		yes	-
*P*. *feulneri* **sp. nov.**	S7989	15	BFD11	-	22.602	59.083	617	KY023447	yes		yes	M849841–M849844
*P*. *feulneri* **sp. nov.**	S7714	15	BFD11	-	22.602	59.083	617	KY023443	-		**yes**	-
*P*. *feulneri* **sp. nov.**	CN4177	15	-	-	22.440	59.265	0	-	-		-	M849859–M849862
*P*. *feulneri* **sp. nov.**	CN4252	15	-	-	22.481	59.283	565	-	-		-	M849855–M849857
*P*. *feulneri* **sp. nov.**	CN8289	15	-	-	22.619	59.094	640	-	yes		-	-
*P*. *feulneri* **sp. nov.**	CN8031	15	-	-	22.619	59.094	640	-	yes		-	-
*P*. *feulneri* **sp. nov.**	CN3717	15	-	-	22.619	59.094	640	-	yes		-	-
*P*. *feulneri* **sp. nov.**	CN8662	15	-	-	22.619	59.094	640	-	yes		-	-
*P*. *feulneri* **sp. nov.**	UAE44	15	-	-	22.620	59.093	674	-	yes		-	-
*P*. *feulneri* **sp. nov.**	UAE46	15	-	-	22.620	59.093	674	-	-		-	-
*P*. *feulneri* **sp. nov.**	S7967	15	-	-	22.602	59.083	617	-	-		-	M849832–M849835
*P*. *feulneri* **sp. nov.**	S6122	15	-	-	22.509	59.126	567	-	yes	*cmos*,*mc1r*,*rag1*,*rag2*	-	-
*P*. *feulneri* **sp. nov.**	S6109	15	-	-	22.602	59.083	617	-	-		-	-
*P*. *feulneri* **sp. nov.**	S6097	15	-	-	22.602	59.083	617	-	-		-	-
*P*. *feulneri* **sp. nov.**	S7175	15	-	-	22.602	59.083	617	-	-		-	-
*P*. *feulneri* **sp. nov.**	CN7125	17	BFD10	-	23.146	57.033	2317	PQ653650	-		**yes**	M849822–M849825
*P*. *feulneri* **sp. nov.**	CN5855	17	BFD10	-	23.281	57.164	2098	PQ653651	-		yes	M849827–M849830
*P*. *feulneri* **sp. nov.**	CN137	17	BFD10	-	23.260	57.217	2100	-	-		yes	-
*P*. *feulneri* **sp. nov.**	CN2792	17	-	-	23.259	57.216	2205	-	-		-	-
*P*. *omanensis* **sp. nov.**	CN2635	8	BFD5	-	23.268	58.917	4	PQ653619	-		yes	-
*P*. *omanensis* **sp. nov.**	S7072	8	BFD5	-	22.845	59.242	28	KY023757	-	*cmos*,*mc1r*,*rag1*,*rag2*	yes	-
*P*. *omanensis* **sp. nov.**	S7187	8	BFD5	-	22.909	59.172	138	KY023758	-	*cmos*,*mc1r*,*rag1*,*rag2*	yes	-
*P*. *omanensis* **sp. nov.**	CN2804	8	-	-	22.972	59.179	6	-	-		-	-
*P*. *omanensis* **sp. nov.**	CN4214	8	-	-	22.956	59.197	8	-	-		-	-
*P*. *omanensis* **sp. nov.**	CN4365	8	-	-	22.954	59.198	8	-	-		-	-
*P*. *omanensis* **sp. nov.**	CN159	8	-	-	22.845	59.241	40	-	yes		-	-
*P*. *omanensis* **sp. nov.**	CN8965	8	-	-	22.845	59.241	40	-	yes		-	-
*P*. *omanensis* **sp. nov.**	CN3588	8	-	-	22.845	59.241	40	-	yes		-	-
*P*. *omanensis* **sp. nov.**	CN7971	8	-	-	22.845	59.241	40	-	yes		-	-
*P*. *omanensis* **sp. nov.**	CN8037	8	-	-	22.845	59.241	40	-	yes		-	-
*P*. *omanensis* **sp. nov.**	S8000	8	-	H—ZFMK104096	22.844	59.242	12	-	yes	*cmos*,*mc1r*,*rag1*,*rag2*	-	-
*P*. *omanensis* **sp. nov.**	S7536	8	-	-	22.909	59.172	138	-	-		-	-
*P*. *omanensis* **sp. nov.**	S7508	8	-	P—IBES7508	22.845	59.242	28	-	yes	*cmos*,*mc1r*,*rag1*,*rag2*	-	M849878–M849882
*P*. *omanensis* **sp. nov.**	S7577	8	-	-	22.845	59.242	28	-	yes		-	-
*P*. *omanensis* **sp. nov.**	CN4374	9	BFD5	-	22.417	59.208	279	PQ653621	-		yes	-
*P*. *omanensis* **sp. nov.**	CN4277	9	BFD5	-	22.107	59.357	195	PQ653627	-		yes	-
*P*. *omanensis* **sp. nov.**	CN4297	9	BFD5	P—IBECN4297	22.709	58.776	457	PQ653623	-		yes	M849873–M849876
*P*. *omanensis* **sp. nov.**	CN170	9	BFD5	-	22.795	58.867	635	PQ653626	-		yes	-
*P*. *omanensis* **sp. nov.**	CN145	9	BFD5	-	22.236	59.226	187	KY023764	yes		**yes**	-
*P*. *omanensis* **sp. nov.**	CN7266	9	BFD5	-	22.236	59.226	187	KY023768	-		yes	-
*P*. *omanensis* **sp. nov.**	S7204	9	BFD5	-	22.683	59.326	550	KY023785	-		**yes**	-
*P*. *omanensis* **sp. nov.**	S7276	9	BFD5	P—IBES7276	22.338	59.311	212	KY023787	yes	*cmos*,*mc1r*,*rag1*,*rag2*	yes	M849893–M849896
*P*. *omanensis* **sp. nov.**	S7246	9	BFD5	-	22.428	59.356	132	KY023786	-	*cmos*,*mc1r*,*rag1*,*rag2*	yes	-
*P*. *omanensis* **sp. nov.**	S9013	9	BFD5	P—IBES9013	22.539	59.368	136	KY023821	yes	*cmos*,*mc1r*,*rag1*,*rag2*	yes	M849888–M849891
*P*. *omanensis* **sp. nov.**	S7797	9	BFD5	-	22.532	59.762	7	KY023810	-		yes	-
*P*. *omanensis* **sp. nov.**	S7778	9	BFD5	-	22.532	59.762	7	KY023809	-		yes	-
*P*. *omanensis* **sp. nov.**	S7717	9	BFD5	-	21.952	59.608	42	KY023801	-		yes	-
*P*. *omanensis* **sp. nov.**	S7497	9	BFD5	-	22.751	59.309	54	KY023792	-		yes	-
*P*. *omanensis* **sp. nov.**	CN3733	9	BFD6	-	22.750	56.597	274	PQ653625	-		yes	M849908–M849911
*P*. *omanensis* **sp. nov.**	CN4304	9	BFD6	-	23.059	56.944	565	PQ653622	-		yes	-
*P*. *omanensis* **sp. nov.**	CN7179	9	BFD6	-	23.150	56.894	552	PQ653620	-		yes	-
*P*. *omanensis* **sp. nov.**	CN4050	9	BFD6	-	23.220	56.974	683	PQ653624	-		yes	M849898–M849901
*P*. *omanensis* **sp. nov.**	CN3597	9	BFD6	-	22.720	58.150	493	KY023765	-		yes	-
*P*. *omanensis* **sp. nov.**	AO20	9	BFD6	-	22.787	57.594	423	KY023763	-		yes	-
*P*. *omanensis* **sp. nov.**	S2137	9	BFD6	-	22.790	57.600	421	KY023776	-		yes	-
*P*. *omanensis* **sp. nov.**	S2126	9	BFD6	-	22.790	57.600	421	KY023775	-		yes	-
*P*. *omanensis* **sp. nov.**	S7460	9	BFD6	-	23.140	57.844	576	KY023791	-	*cmos*,*mc1r*,*rag1*,*rag2*	**yes**	-
*P*. *omanensis* **sp. nov.**	S7744	9	BFD6	-	23.118	56.778	476	KY023806	-		**yes**	-
*P*. *omanensis* **sp. nov.**	S7716	9	BFD6	-	22.378	57.514	271	KY023800	-		yes	-
*P*. *omanensis* **sp. nov.**	S7457	9	BFD6	-	23.096	57.310	654	KY023790	yes		yes	M849903–M849906
*P*. *omanensis* **sp. nov.**	S7541	9	-	P—NHMOK2657	22.833	58.988	1005	KY023793	yes	*cmos*,*mc1r*,*rag1*,*rag2*	yes	-
*P*. *omanensis* **sp. nov.**	CN4707	9	-	-	22.795	58.867	635	-	-		-	-
*P*. *omanensis* **sp. nov.**	CN4036	9	-	-	22.417	59.208	279	-	-		-	-
*P*. *omanensis* **sp. nov.**	CN4333	9	-	-	22.408	59.224	252	-	-		-	-
*P*. *omanensis* **sp. nov.**	CN4326	9	-	-	22.172	59.417	109	-	-		-	-
*P*. *omanensis* **sp. nov.**	CN3477	9	-	-	22.750	56.600	274	-	-		-	-
*P*. *omanensis* **sp. nov.**	CN4275	9	-	-	22.750	56.601	276	-	-		-	-
*P*. *omanensis* **sp. nov.**	CN7281	9	-	-	23.059	56.944	565	-	-		-	-
*P*. *omanensis* **sp. nov.**	CN4327	9	-	-	23.150	56.894	552	-	-		-	-
*P*. *omanensis* **sp. nov.**	CN7265	9	-	-	23.221	56.974	683	-	-		-	-
*P*. *omanensis* **sp. nov.**	CN228	9	-	-	22.709	58.776	457	-	-		-	-
*P*. *omanensis* **sp. nov.**	CN2812	9	-	-	22.451	59.826	12	-	-		-	-
*P*. *omanensis* **sp. nov.**	CN8142	9	-	-	22.495	58.681	0	-	-		-	-
*P*. *omanensis* **sp. nov.**	CN7006	9	-	-	22.496	58.684	0	-	-		-	-
*P*. *omanensis* **sp. nov.**	CN4039	9	-	-	22.214	58.092	0	-	-		-	-
*P*. *omanensis* **sp. nov.**	CN8966	9	-	-	22.220	58.092	0	-	-		-	-
*P*. *omanensis* **sp. nov.**	CN3601	9	-	-	22.090	58.002	0	-	-		-	-
*P*. *omanensis* **sp. nov.**	CN8295	9	-	-	22.090	58.002	0	-	-		-	-
*P*. *omanensis* **sp. nov.**	CN7251	9	-	-	22.571	59.100	584	-	-		-	-
*P*. *omanensis* **sp. nov.**	CN4023	9	-	-	22.236	59.226	187	-	yes		-	-
*P*. *omanensis* **sp. nov.**	S1540	9	-	-	22.369	58.059	318	-	-		-	-
*P*. *omanensis* **sp. nov.**	S1891	9	-	-	22.790	57.600	421	-	yes		-	-
*P*. *omanensis* **sp. nov.**	S1881	9	-	-	23.062	57.130	583	-	-		-	-
*P*. *omanensis* **sp. nov.**	S1645	9	-	-	23.062	57.130	583	-	yes		-	-
*P*. *omanensis* **sp. nov.**	S1757	9	-	-	22.811	57.602	418	-	-		-	-
*P*. *omanensis* **sp. nov.**	S1796	9	-	-	22.811	57.602	418	-	-		-	-
*P*. *omanensis* **sp. nov.**	S8002	9	-	-	22.040	59.323	102	-	-		-	-
*P*. *omanensis* **sp. nov.**	S6111	9	-	-	22.650	58.863	465	-	-		-	-
*P*. *omanensis* **sp. nov.**	S6113	9	-	-	22.782	57.851	459	-	-		-	-
*P*. *omanensis* **sp. nov.**	S7437	9	-	-	23.096	57.310	654	-	yes		-	-
*P*. *omanensis* **sp. nov.**	S7934	9	-	-	22.439	58.776	296	-	-		-	-
*P*. *omanensis* **sp. nov.**	S7706	9	-	-	22.464	59.052	322	-	-		-	-
*P*. *omanensis* **sp. nov.**	S7849	9	-	-	22.625	58.657	362	-	-		-	-
*P*. *omanensis* **sp. nov.**	S6125	9	-	-	23.013	56.980	582	-	-		-	M849918–M849922
*P*. *omanensis* **sp. nov.**	S7727	9	-	-	22.842	58.391	567	-	-		-	-
*P*. *omanensis* **sp. nov.**	S7865	9	-	-	23.377	56.682	491	-	yes	*cmos*,*mc1r*,*rag1*,*rag2*	-	-
*P*. *omanensis* **sp. nov.**	S6114	9	-	-	22.264	59.258	175	-	-		-	-
*P*. *omanensis* **sp. nov.**	S6146	9	-	-	22.505	58.748	318	-	-		-	-
*P*. *omanensis* **sp. nov.**	S6117	9	-	-	22.596	58.824	400	-	-		-	-
*P*. *omanensis* **sp. nov.**	S7165	9	-	-	22.228	59.185	167	-	-		-	-
*P*. *omanensis* **sp. nov.**	S7954	9	-	-	22.588	58.684	348	-	-		-	-
*P*. *omanensis* **sp. nov.**	S7719	9	-	-	21.842	59.567	13	-	-	*cmos*,*mc1r*,*rag1*,*rag2*	-	-
*P*. *omanensis* **sp. nov.**	S7911	9	-	-	22.378	57.514	271	-	-	*cmos*,*mc1r*,*rag1*,*rag2*	-	-
*P*. *omanensis* **sp. nov.**	S7669	9	-	-	22.532	59.762	7	-	-		-	-
*P*. *omanensis* **sp. nov.**	S7853	9	-	-	23.061	57.131	591	-	-		-	-
*P*. *omanensis* **sp. nov.**	S7737	9	-	P—MZB 2024–0987	21.952	59.608	42	-	-		-	M849864–M849867
*P*. *omanensis* **sp. nov.**	S9012	9	-	P—MZB 2024–0988	22.428	59.356	132	-	yes	*cmos*,*mc1r*,*rag1*,*rag2*	-	M849884–M849886
*P*. *omanensis* **sp. nov.**	S9014	9	-	-	22.523	59.414	50	-	-		-	-
*P*. *omanensis* **sp. nov.**	S9015	9	-	-	22.751	59.309	54	-	-		-	-
*P*. *omanensis* **sp. nov.**	S9019	9	-	-	22.874	58.925	545	-	-		-	-
*P*. *omanensis* **sp. nov.**	S7552	9	-	-	22.763	58.853	591	-	-	*cmos*,*mc1r*,*rag1*,*rag2*	-	-
*P*. *omanensis* **sp. nov.**	S7434	9	-	-	23.118	56.778	476	-	-		-	-
*P*. *omanensis* **sp. nov.**	S7886	9	-	-	22.532	59.762	7	-	-		-	-
*P*. *omanensis* **sp. nov.**	S7763	9	-	-	21.842	59.567	13	-	-		-	-
*P*. *omanensis* **sp. nov.**	S7753	9	-	-	21.952	59.608	42	-	-		-	-
*P*. *omanensis* **sp. nov.**	S7708	9	-	P—IBES7708	21.842	59.567	13	-	-		-	M849869–M849871
*P*. *omanensis* **sp. nov.**	S7721	9	-	-	21.842	59.567	13	-	-		-	-
*P*. *omanensis* **sp. nov.**	S7564	9	-	-	22.428	59.356	132	-	-		-	-
*P*. *omanensis* **sp. nov.**	S9011	9	-	-	22.338	59.311	212	-	-		-	-
*P*. *omanensis* **sp. nov.**	S7171	9	-	-	22.782	57.851	459	-	-		-	M849913–M849916
*P*. *omanensis* **sp. nov.**	S7590	9	-	-	22.428	59.356	132	-	-		-	-
*P*. *flavipunctatus*	CN12676	*-*	*-*	-	9.136	40.163	817	PQ653665	-		yes	-
*P*. *flavipunctatus*	CN12693	*-*	*-*	-	10.300	47.183	1758	PQ653664	-		yes	-
*P*. *flavipunctatus*	JEM422	*-*	*-*	-	13.017	44.550	297	PQ653676	-		yes	-
*P*. *flavipunctatus*	JEM442	*-*	*-*	-	13.350	43.950	1182	PQ653674	-		yes	-
*P*. *flavipunctatus*	JEM227	*-*	*-*	-	13.517	43.950	1253	PQ653669	-		yes	-
*P*. *flavipunctatus*	JEM238	*-*	*-*	-	13.867	45.800	1151	PQ653671	-		yes	-
*P*. *flavipunctatus*	JEM239	*-*	*-*	-	13.867	45.800	1151	PQ653670	-		yes	-
*P*. *flavipunctatus*	JEM125	*-*	*-*	-	14.217	47.067	970	PQ653672	-		yes	-
*P*. *flavipunctatus*	JEM170	*-*	*-*	-	14.650	44.200	1846	PQ653673	-		yes	-
*P*. *flavipunctatus*	JEM169	*-*	*-*	-	14.900	43.467	0	PQ653675	-		yes	-
*P*. *flavipunctatus*	CN517	*-*	*-*	-	15.610	38.957	800	PQ653667	-		yes	-
*P*. *flavipunctatus*	CN518	*-*	*-*	-	15.610	38.957	800	PQ653668	-		**yes**	-

Additionally, we implemented independent network analyses with four nuclear markers including specimens from all candidate species of the *P*. *rupestris* species complex. Sequences were downloaded from GenBank (see accession numbers within [27]), and corresponded to genes *mc1r* (73 sequences; 669 bp), *cmos* (72 sequences; 411 bp), *rag1* (73 sequences; 279 bp), and *rag2* (67 sequences; 405 bp). SEQPHASE [52] was used to convert input files and haplotypes were phased with PHASE [53]. Then, genealogical relationships for each gene were assessed with TCS v.1.21 [54] constructing haplotype networks with statistical parsimony and applying a connection limit of 95%.

### Genomic data

#### ddRADseq data analyses of genome-wide SNPs

Several datasets of genome-wide SNP data inferred from Burriel-Carranza et al. (2024) [28] were used to perform population genomic, phylogenomic and species delimitation analyses. The following datasets were retrieved or assembled: i) *dataset1*: Dataset composed of 163 specimens from the *P*. *rupestris* species complex including all known lineages with 491 unlinked SNPs (retaining one SNP per locus) and a dataset completeness of 77% (dataset retrieved from [28], corresponding to dataset 16 in the former study); ii) *dataset2*: Dataset composed of the same 163 specimens produced *de novo* to obtain in addition invariant sites. Raw illumina reads were downloaded from Burriel-Carranza et al. (2024) [28] and processed with iPyRAD [55]. We discarded reads with ≥ 3 missing sites, consensus sequences with low coverage (<10 reads), excessive undetermined or heterozygous sites (>3), or too many haplotypes (>2). Consensus sequences were clustered across samples using the within-sample clustering threshold (89%). The resulting dataset contained 104,277 loci and 346,583 SNPs. When this dataset was used, further filtering options were applied for each analysis; iii) *dataset3*: Dataset composed of 173 *Pristurus* specimens including 10 individuals of *P*. *flavipunctatus* and 163 specimens from the *P*. *rupestris* species complex, with a total length of 137,800 bp including invariant sites (20,845 SNPs), and a dataset completeness of 70.87% (dataset retrieved from [28], corresponding to dataset 31 in the former study); iv) *dataset4*: Multispecies Coalescent (MSC) species tree reconstructions and species delimitation methods were implemented to a reduced dataset of 31 specimens, containing the specimens with the least missingness and with no signs of admixture from at least two representatives from each deep lineage identified by Burriel-Carranza et al. (2024) [28], and two samples from *P*. *flavipunctatus* used as outgroups. The dataset was obtained following the same procedure as in *dataset2* but including a hard filtering of missing genotype call rate of 40% (i.e. all loci were present in at least 60% of the samples).

#### Population genomics

Burriel-Carranza et al. (2024) [28] performed a set of several hierarchical admixture analyses to discover the most probable number of populations within the *Pristurus rupestris* species complex. This showed that deep lineages of the *P*. *rupestris* species complex are highly structured following the local topography of the mountain range with no signs of current admixture between the deep lineages of the *Pristurus rupestris* species complex (see S23, S38–S43 Figs from Burriel-Carranza et al 2024) [28]. Here, we further complement this data by implementing two other population genomic analyses. First, we performed a Principal Component Analysis (PCA) on *dataset1* using Plink v2.00a2.3 [56]. Then, a fineRADstructure [57] analysis was implemented on *dataset2* applying a hard filtering of missing genotype call rate of 40%. This analysis unravels different levels of structure within and between populations and its robustness to missing data is optimal for non-model organisms [57].

#### Genetic divergence

We assessed the levels of intra- and interspecific genetic divergence across all lineages of the *Pristurus rupestris* species complex. We used *dataset2* with a SNP data assembly allowing a maximum of 40% missing data at each locus, with a total of 2,712 SNPs. We clustered the samples into groups representing the 12 *P*. *rupestris* independent lineages identified in Burriel-Carranza et al. (2024) [28]. Then, we used all variant sites to calculate pairwise F_ST_ and d_xy_ across all lineage-pairs. Measures were calculated with the package PopGenome [58] implemented in R v.4.2.1 [59].

#### Phylogenomic reconstructions

We used *dataset3* to generate a ML phylogenomic reconstruction as a means to compare the nuclear evolutionary history of the *P*. *rupestris* species complex to the mitochondrial evolutionary history of this group (see above). The phylogenomic reconstruction was inferred through RAxML-ng v.1.0.2 [50], with a GTR+G model, a total of 100 starting trees (50 random and 50 parsimony) and 1,000 bootstrap replicates. Then, we further investigated the phylogenomic relationships within the *P*. *rupestris* species complex by generating a time-calibrated species tree inferred with SNAPP v.1.5.2 [60] under the multispecies coalescent model. We used *dataset4* excluding invariant sites, keeping one biallelic SNP per locus, applying a minor allele frequency filter of 0.05, and discarding SNPs that were not present in at least 80% of the samples. We converted the resulting vcf file following Stange et al. (2018) [61] script *snapp_prep*.*rb* (available at: https://github.com/mmatschiner/snapp_prep), summarizing 2,759 SNPs. The deepest node of the phylogeny (i.e., the split between *P*. *flavipunctatus* and all representatives of the *P*. *rupestris* species complex) was constrained to be monophyletic and set to a normal distribution with a mean of 20.92 mya and *σ* = 1.6, following the latest calibration [28]. Samples were then assigned to their corresponding lineage (as inferred in Burriel-Carranza et al. 2024) [28] and three independent runs of 3x10^6^ generations were carried out sampling every 50 generations. Convergence between runs and stationarity was checked with Tracer v.1.7 [62]. Posterior distributions were combined with LogCombiner v.2.6.3, discarding 10% of the posterior trees as burnin and a maximum clade credibility tree was obtained calculating median heights in TreeAnnotator v.2.6.3 (both programs implemented in BEAST2 v.2.6.4; [63]).

### Species delimitation

Using several mitochondrial and nuclear markers, Garcia-Porta et al. (2017) [27] found evidence of up to 14 putative species within the *P*. *rupestris* species complex. Using genome-wide SNP data, Burriel-Carranza et al. (2024) [28] found that 12 of such putative species were still recovered as distinct species when using Bayes Factor Delimitation* (*with genomic data; BFD; [64]). In this study, we further investigate these findings by implementing guided species delimitation methods by means of BPP v.4.4.1 [65, 66]). We followed Huang (2018) [67] pipeline (*https*:*//github*.*com/airbugs/Dynastes_delimitation*) to estimate *θ* and τ priors (a = 3, b = 0.06 and a = 3, b = 0.15 respectively) on *dataset4*, selecting those loci present in at least one specimen from each putative species. The species tree topology was fixed to match a Multispecies Coalescent (MSC) species tree estimated above. Then, three independent runs of BPP A10 species delimitation [65] were implemented, with 1,000,000 generations, sampling every 10 generations after a burn-in of 100,000.

Then, we calculated the *gdi* index for each putative species using the equation *gdi = 1-e^(-2τ/θ)^* [37]. Posterior distributions for *θ* and τ were estimated with BPP analysis, A00 [65]. We used *dataset3* with the same prior specification and species tree topology as above. Three independent runs of BPP A00 [65] analyses were performed for 100,000 generations, sampling every five generations, and applying a burnin of 10,000. Although the *gdi* can account for both genetic isolation and gene flow, it was calculated assuming no gene flow, as this procedure has been shown to provide more accurate species delimitations than the original estimation with PHRAPL [68], even in the presence of gene flow [39, 69]. The *gdi* is a continuous index between 0 (panmixia) and 1 (strong divergence between two sister taxa) and, while there is not a specific “species delimitation threshold”, Jackson et al. (2017) [68] proposed that *gdi* > 0.7 supports a species status of two sister taxa and *gdi* < 0.2 supports a single species hypothesis. However, there is a wide range of uncertainty between 0.2–0.7 which is considered as an ambiguous result, lacking the support necessary to be classified as distinct species.

Using the same posterior distributions of *θ* and τ obtained to estimate the *gdi*, we calculated another measure of genetic divergence between populations of the *P*. *rupestris* species complex. This was obtained by calculating divergence times in coalescent units (2τ/*θ*) for each species and population using all samples from the combined posterior distributions following a similar approach as in Leaché et al. (2021) [39].

### Sympatry analyses

We assessed the geographic overlap of species within the *P*. *rupestris* species complex by evaluating the number of sympatric localities between species. We generated a 50 m buffer to 604 localities of *P*. *rupestris* species complex specimens (extracted from [33, 34]) and determined the number of samples that were found within each buffer. A buffer of only 50 m was chosen due to the complex topography and dramatic altitudinal shifts of the Hajar Mountains. The analysis was performed within R v.4.2.1 [59] using packages ‘terra’ [70] and ‘sf’ [71].

### Morphological description and designation of type specimens of the *Pristurus rupestris* species complex

We chose and examined a total of 29 alcohol-preserved adult specimens of the *P*. *rupestris* species complex from the Hajar Mountains. All specimens were photographed using a Nikon D750 camera with a 60 mm macro-lens. Voucher specimens are deposited at the Institute of Evolutionary Biology (IBE), Barcelona, Spain, the Zoologisches Forschungsmuseum Alexander Koenig (ZFMK), Bonn, Germany, the Naturhistorisk Museum (NHMO), Oslo, Norway, and the Oman Natural History Museum (ONHM), Muscat, Oman. Variables for the morphological analyses were selected based on previous taxonomic studies of *Pristurus* [18, 19, 23, 25, 27, 72]. Measurements were taken by the same author (T.K.) using a digital calliper (to the nearest 0.1 mm). We measured the following morphometric characters (see section “Morphological analyses” above for explanation of the different measurements): snout–vent length (SVL); trunk length (TrL); head length (HL); head height (HH); humerus length (LHu); ulna length (LUn); femur length (LFe); and tibia length (LTb). Tail length was not measured because many individuals had a regenerated tail or had lost it. In addition to those morphometric variables, we examined two pholidotic characters, counting from both the right and left sides: the number of upper labial scales (ULS) and the number of lower labial scales (LLS; anterior large scales only; S1 Table).

### Nomenclatural Acts

The electronic edition of this article conforms to the requirements of the amended International Code of Zoological Nomenclature, and hence the new names contained herein are available under that Code from the electronic edition of this article. This published work and the nomenclatural acts it contains have been registered in ZooBank, the online registration system for the ICZN. The ZooBank LSIDs (Life Science Identifiers) can be resolved and the associated information viewed through any standard web browser by appending the LSID to the prefix “http://zoobank.org/”. The LSID for this publication is: urn:lsid:zoobank.org:pub:EB6D62A2-E43E-43C5-A8DA-94E52277C067. The electronic edition of this work was published in a journal with an ISSN, and has been archived and is available from the following digital repositories: PubMed Central, LOCKSS.

## Results

**Fig 2:**
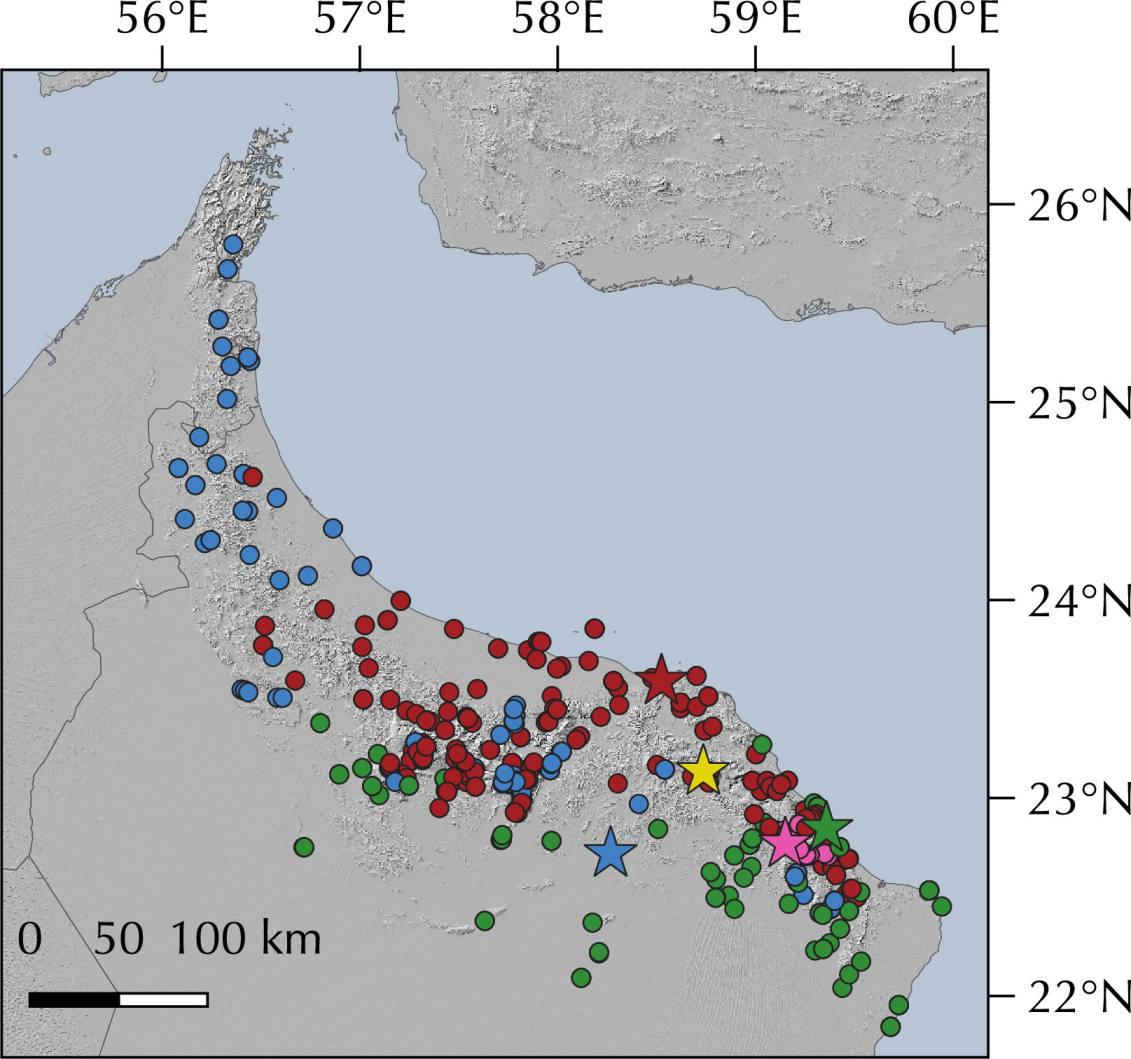
Geographic map showing 605 genetically identified specimens of the four new species of *Pristurus* described in this study and *Pristurus rupestris* sensu stricto, making apparent the great deal of geographic overlap between these cryptic species. Red: *Pristurus rupestris* sensu stricto; Green: *Pristurus omanensis* **sp. nov.**; Pink: *Pristurus ali* **sp. nov.**; Blue *Pristurus feulneri* **sp. nov.**; Yellow: *Pristurus assareen* **sp. nov.**. Type localities for each species are represented with a star. Further detailed maps for each species can be consulted in Figs 8, 12, 16, 20 and 24 in order of appearance above.

**Fig 3:**
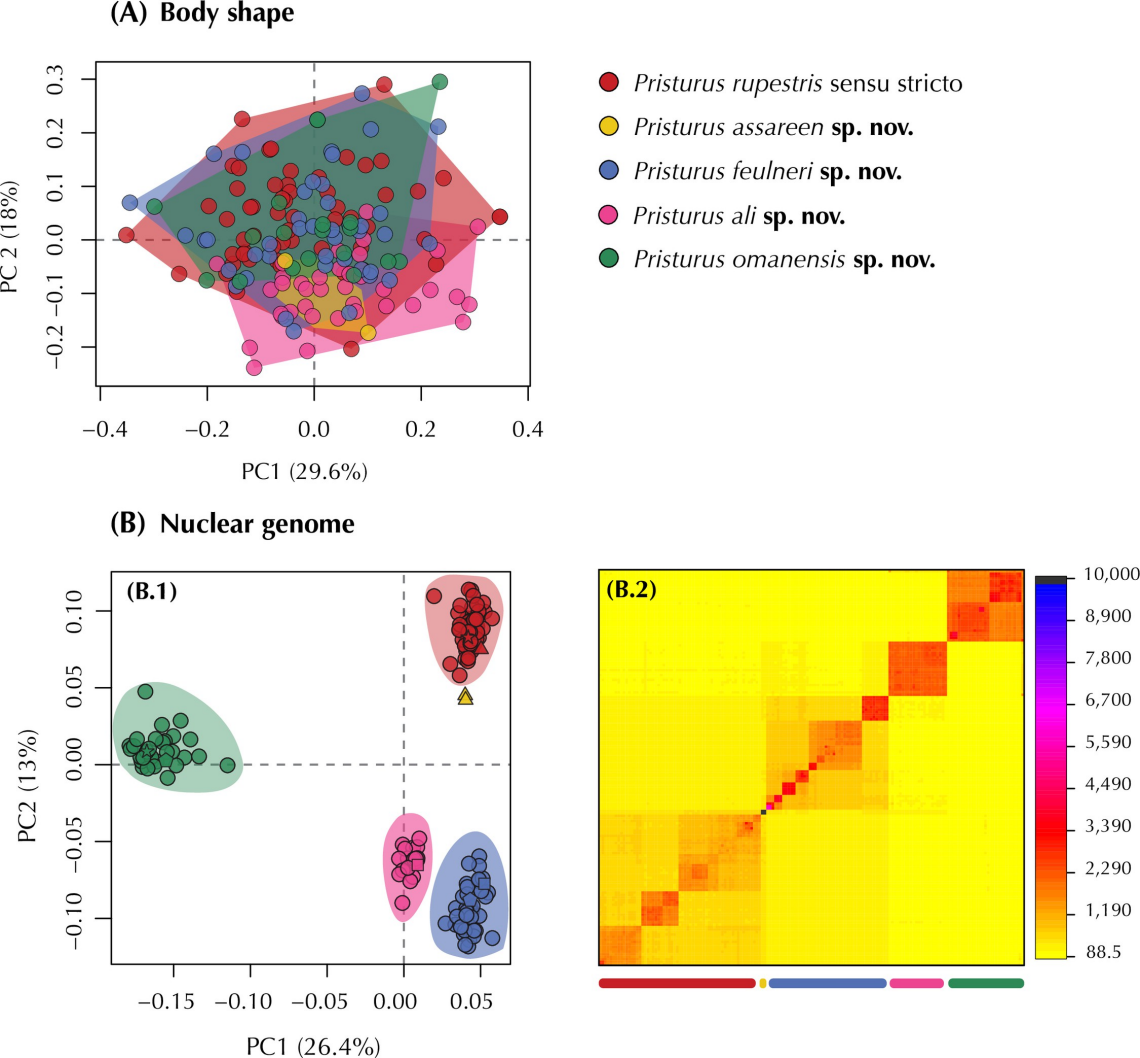
**(A)** Reconstructed morphospace of the *Pristurus rupestris* species complex represented by the first two Principal Components of the PCA (explaining almost 50% of all shape variance). Morphospace was constructed based on 10 independent measurements of 155 specimens from which the body size effect was removed (Table 1; see see Materials and Methods). The PCA highlights the great shape overlap across these *Pristurus* species. **(B)** Population structure analyses with genomic data. **(B.1)** PCA representing 163 specimens of the *Pristurus rupestris* species complex inferred with genomic *dataset1* (Table 1; see Materials and Methods) summarizing 39.4% of the genomic space. Both first and second principal components split species-level diversity, showing up to four well-defined genomic clusters, and *Pristurus assareen* **sp. nov.** (yellow) being only slightly differentiated from *Pristurus rupestris* sensu stricto (red); **(B.2)** Co-ancestry matrix generated through fineRADstructure implemented on the same 163 specimens using *dataset2* (Table 1; see Materials and Methods). This analysis indicates pairwise genomic similarity across all specimens, unraveling different levels of structure within and between species. Legend to the right shows the number of shared haplotypes between individuals. Darker colors represent higher inter-specimen co-ancestry.

**Fig 4:**
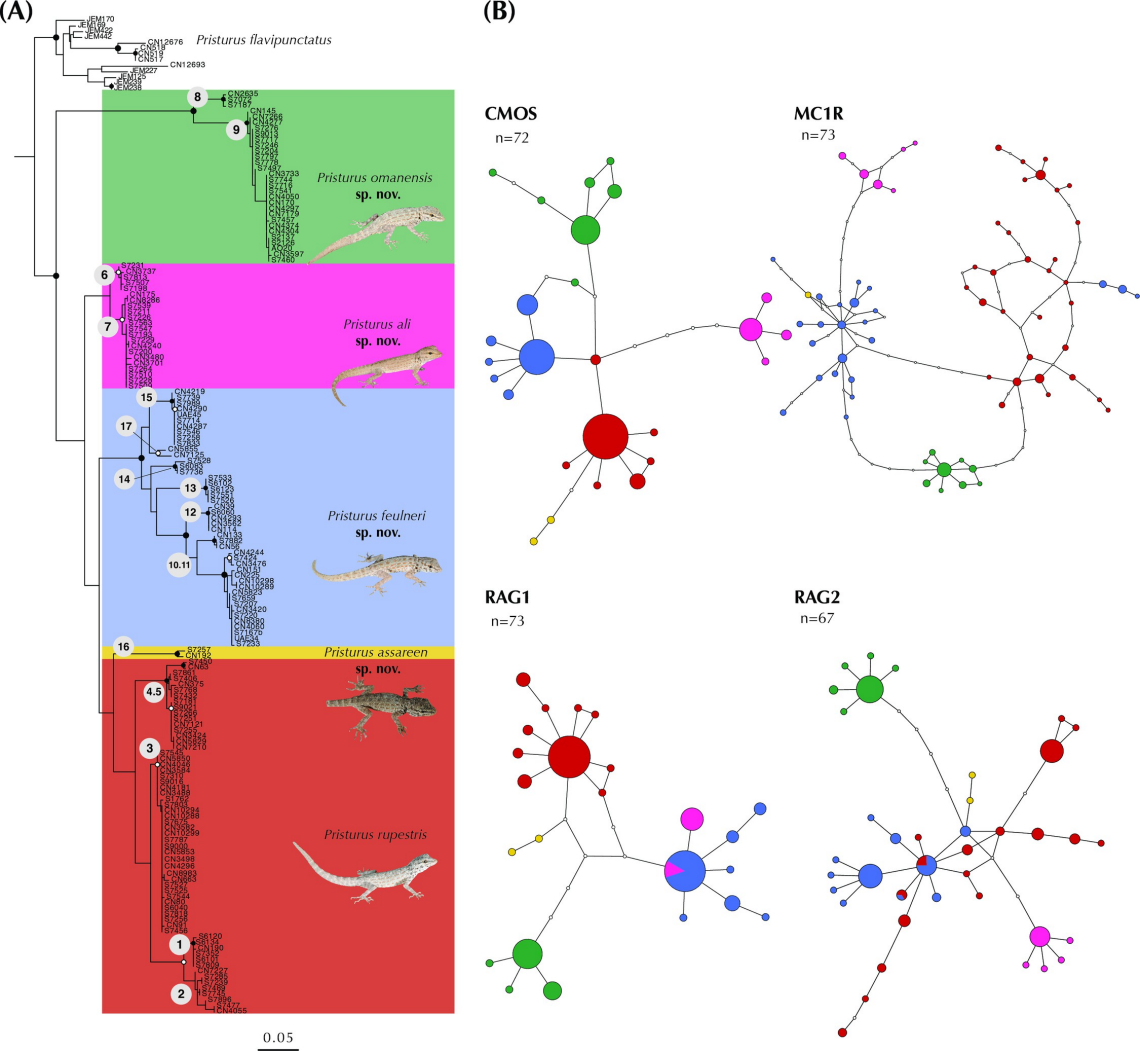
Genetic analyses. **(A)** Maximum Likelihood phylogenetic reconstruction. A RAxML-NG mitochondrial phylogeny of the *Pristurus rupestris* species complex was inferred using the mitochondrial marker 12S on 163 *Pristurus* specimens from the *P*. *rupestris* species complex, and 10 *P*. *flavipunctatus* used as outgroups (Table 1). Numbers in grey circles represent the lineages considered as putative species in Garcia-Porta et al. (2017), including a newly discovered deep lineage (lineage 17). Black dots: bootstrap support (bs) > 0.95; White dots: 0.75 < bs > 0.95. Different background colors represent the newly described species. **(B)** Statistical parsimony allele networks of *cmos*, *mc1r*, *rag1* and *rag2* nuclear loci, with colors corresponding to species in the phylogenetic tree. Circle sizes are proportional to the number of alleles. White circles represent mutational steps.

**Fig 5:**
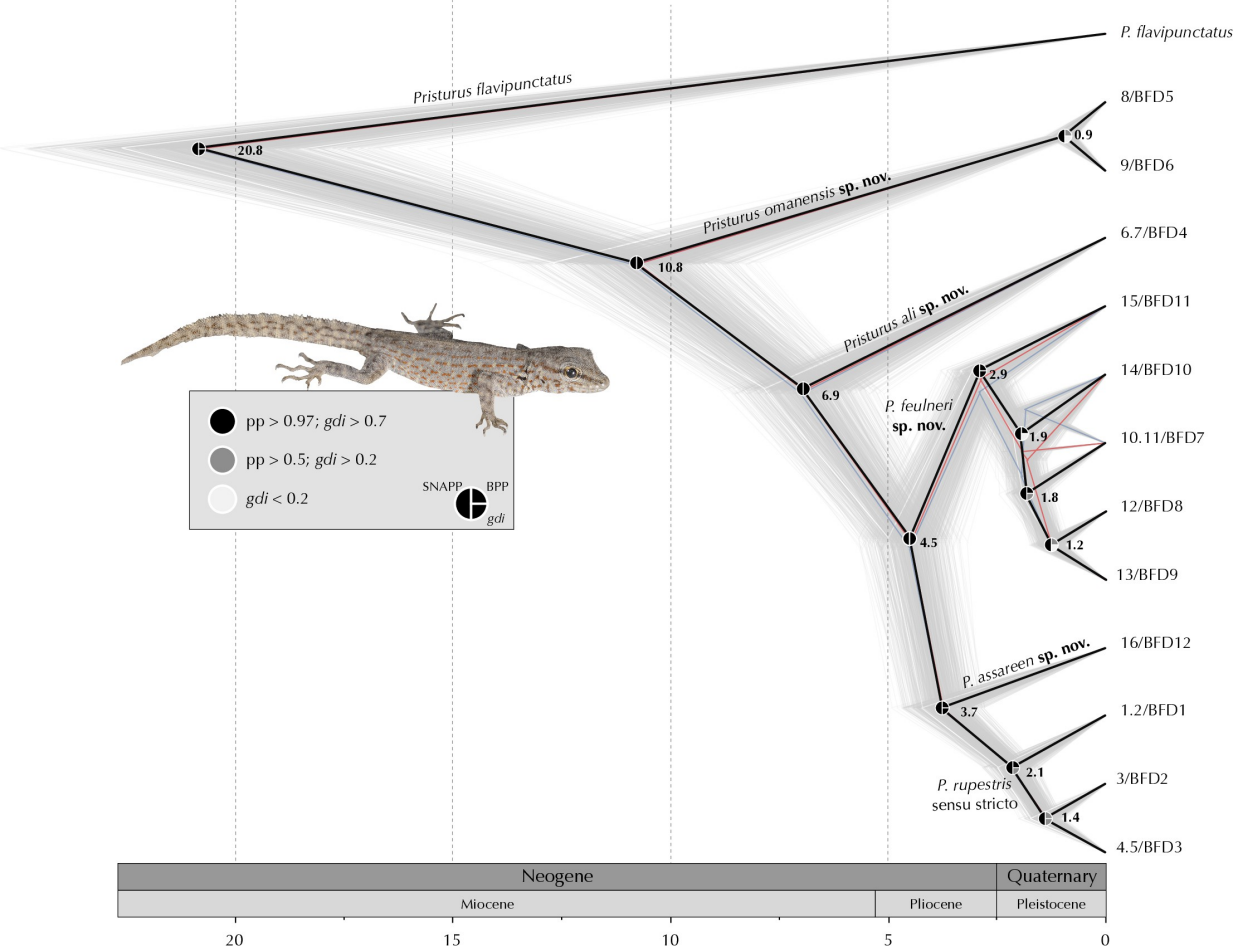
Genomic phylogeny of the *Pristurus rupestris* species complex. Time-calibrated Multispecies Coalescent species tree inferred with 29 specimens from the *Pristurus rupestris* species complex and two *P*. *flavipunctatus* used as outgroups, summarizing 2,759 SNPs (*dataset4*; Table 1). Three consensus trees were found and are shown in black (53% of all tree topologies), red (31%), and blue (16%) respectively. Mean divergence times are shown at each node with a cloudgram (light grey lines in the background) representing the uncertainty within divergence time estimates. Tip labels encompass genetic lineages described in Garcia-Porta et al. (2017) and genomic lineages identified in Burriel-Carranza et al. (2023b) separated by a slash. At each node, posterior probabilities (pp) for presence of nodes represented, as well as results from two species delimitation methods: Posterior probabilities for presence of nodes in BPP A10 analysis and the mean value for the posterior distribution of the genealogical divergence index (*gdi*). For specific ranges of *gdi* posterior distributions see S2 Fig.

**Fig 6:**
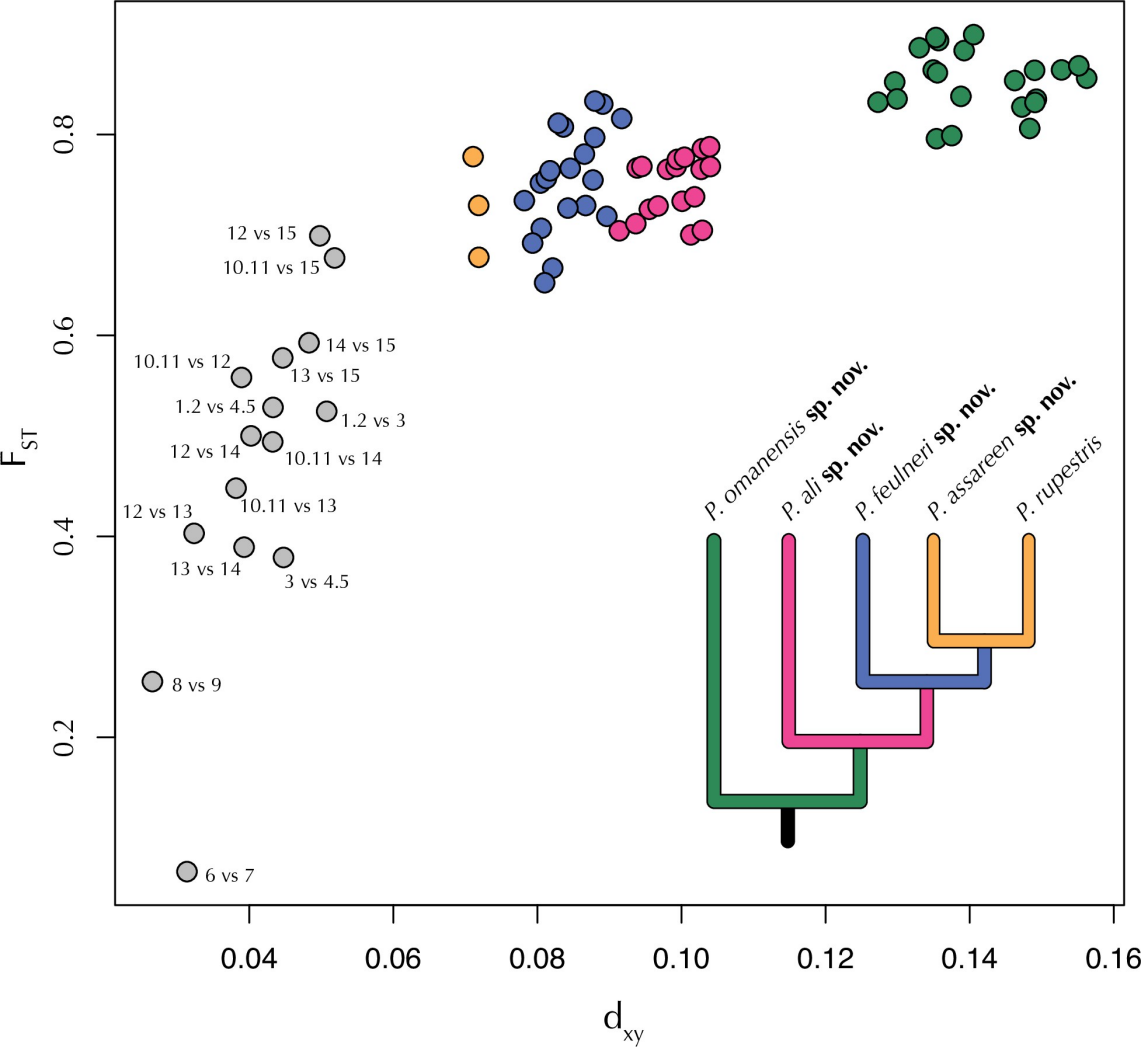
F_ST_ and d_xy_ pairwise comparisons among all *Pristurus rupestris* species complex genetic lineages. Colored dots correspond to interspecific pairwise comparisons between lineages from one species (represented by the coloured line in the phylogeny) and all lineages within its sister clade. Grey dots show intraspecific pairwise comparisons. Numbers at each grey dot correspond to genetic lineages *sensu* Garcia-Porta et al. (2017).

The combination of morphological (Fig 3A and S1–S4 Tables), genetic (Fig 4 and Tables 1 and S5), genomic (Figs 3B, 3C, 5, 6, S1, S2 and S6 Table) and geographic (S7 Table) analyses support the existence of at least five species within the *Pristurus rupestris* species complex (Fig 2). The comparisons of the cranial osteology for the single holotype specimens (Figs 10B, 10C, 14B, 14C, 18B, 18C, 22B and 22C) of each species did not recover strong qualitative osteological differences between species of the *P*. *rupestris* species complex. Minor qualitative osteological differences and differences in the extent of ossification are described for the holotypes only in the species account section. Although these species share striking morphological similarities and may therefore be considered as phenotypically cryptic (Fig 3A and S2–S4 Tables), each one exhibits unique nuclear haplotypes, is recovered in both mitochondrial and nuclear phylogenetic and phylogenomic reconstructions as monophyletic, is considered a distinct species in all species delimitation analyses, and has an ancient origin of at least three mya. Notably, these species coexist within the same geographical areas (Fig 2), often in sympatry or syntopy with at least one other species of the species complex (S7 Table). However, no signs of gene flow occurring among any of such taxa were detected (Figs 3B, S23 and S38–S43 from Burriel-Carranza et al 2024) [28], hence supporting the existence of pre- or postzygotic barriers and stable species boundaries.

### Systematics

Family **Sphaerodactylidae** Underwood, 1954
Genus ***Pristurus*** Rüppell, 1835
***Pristurus rupestris* Blanford, 1874**

(Figs 2–9, S1, S2 and Tables 1 and S1–S7; Alignment of phased haplotypes for the molecular diagnostic characters based on the *mc1r*, *cmos*, *rag1* and *rag2* genes can be found on S1 Appendix).

**Fig 7:**
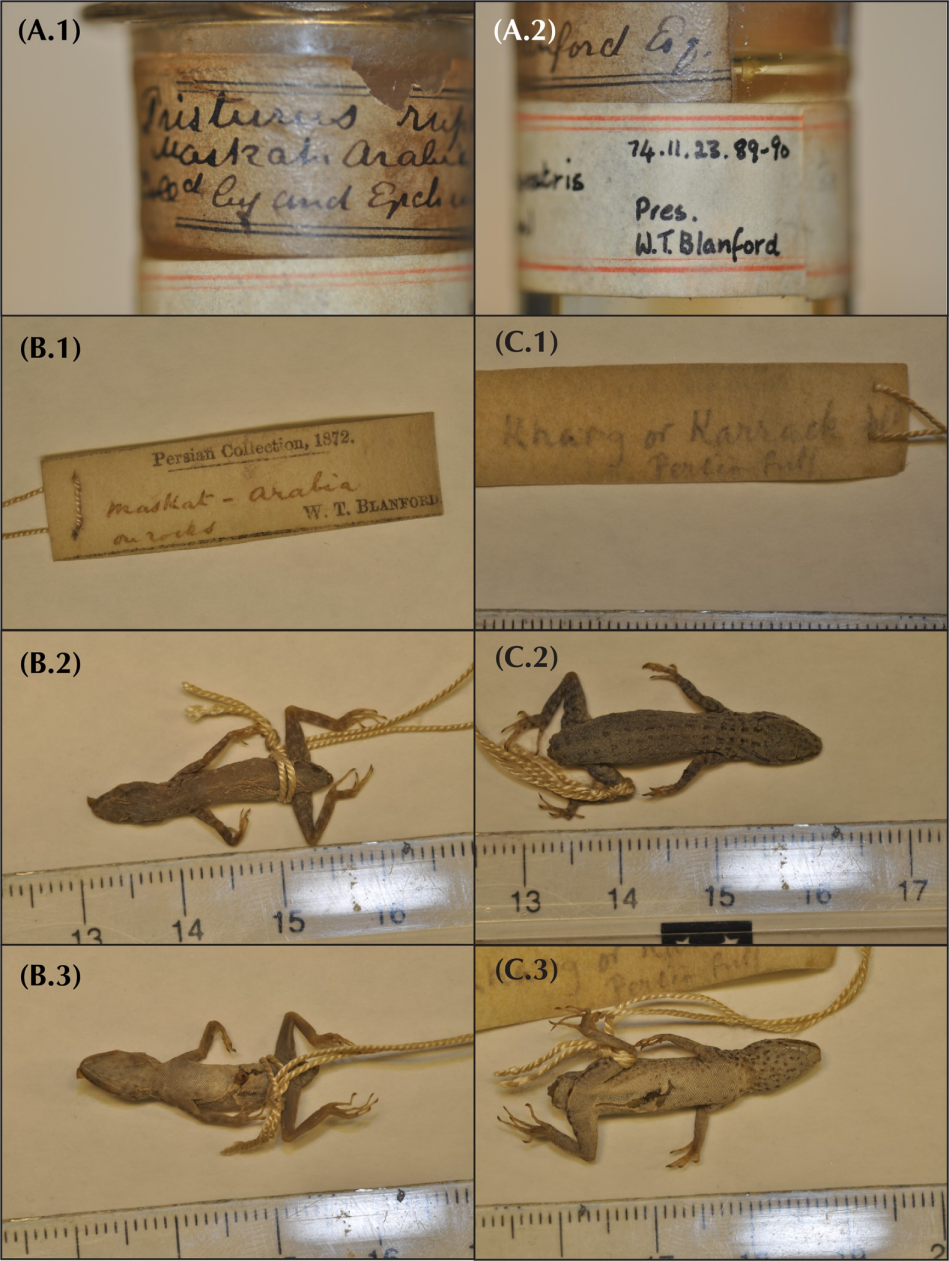
*Pristurus rupestris* type material deposited by W.T. Blanford in 1874, in the Natural History Museum of London (NHMUK) with catalog number 1874.11.23.89–90. **(A.1-2)** Jar where both *P*. *rupestris* specimens shown below are stored; **(B)** Tag **(B.1)**, dorsal view **(B.2)**, and ventral view **(B.3)** of the *Pristurus rupestris* syntype from the type locality (Muscat, Oman), described as a lectotype in the present study; **(C)** Tag **(C.1)**, dorsal view **(C.2)**, and ventral view **(C.3)** of the *Pristurus rupestris* syntype from Kharg island, Iran find in the same jar as the lectotype. All pictures taken by JG-P.

**Fig 8:**
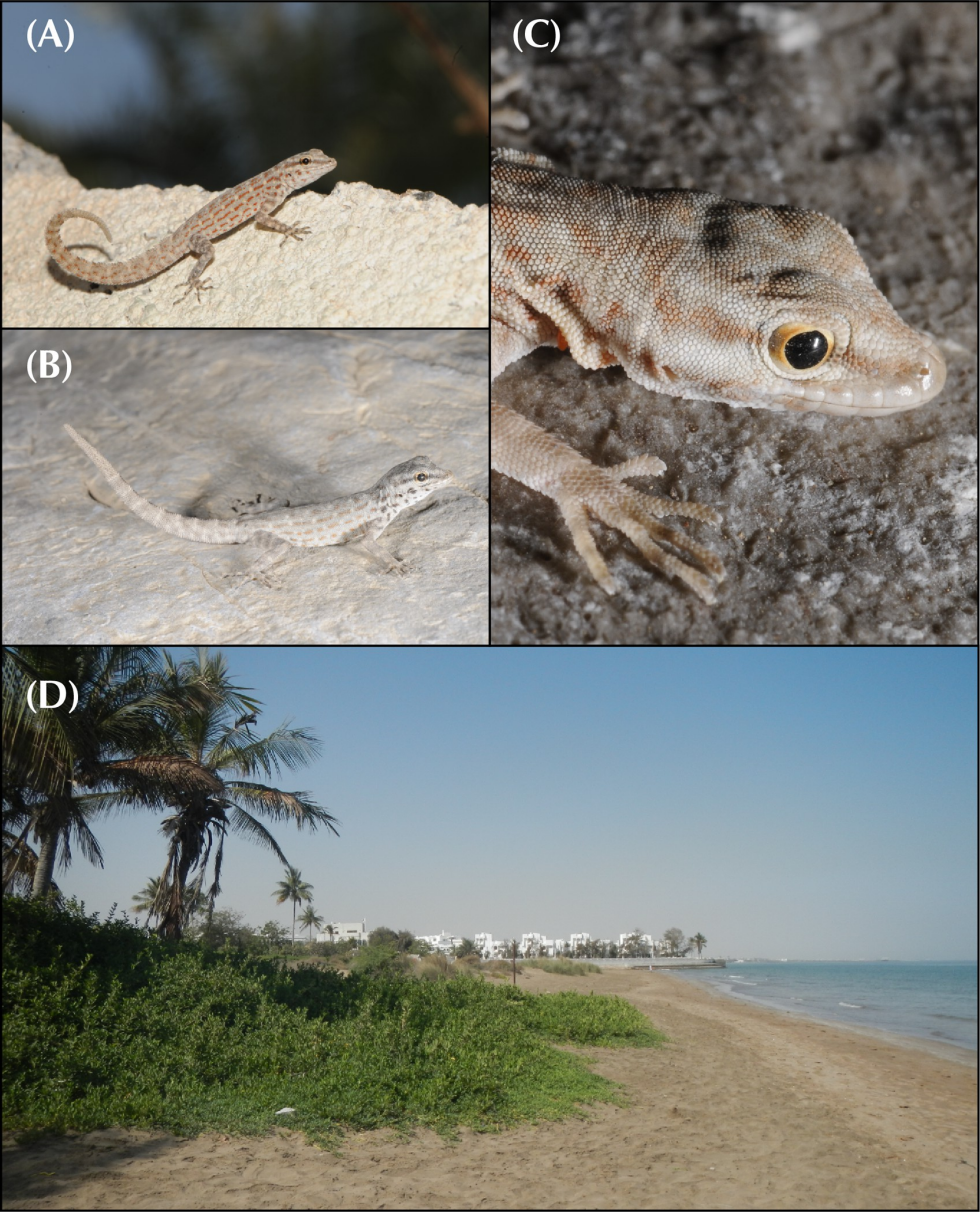
**(A)** *Pristurus rupestris* sensu stricto male specimen signaling by curling its tail (picture code: *DSC7726)* **(B)** Male specimen with paler coloration and less apparent upper and lower crest in tail from lineage 3 *sensu* Garcia-Porta; **(C)** Head close-up on a *P*. *rupestris* sensu stricto specimen from lineage 3 *sensu* Garcia-Porta et al., (2017); **(D)** Picture of Muscat beach, type locality of *P*. *rupestris* sensu stricto. All pictures taken by S.C.

**Fig 9:**
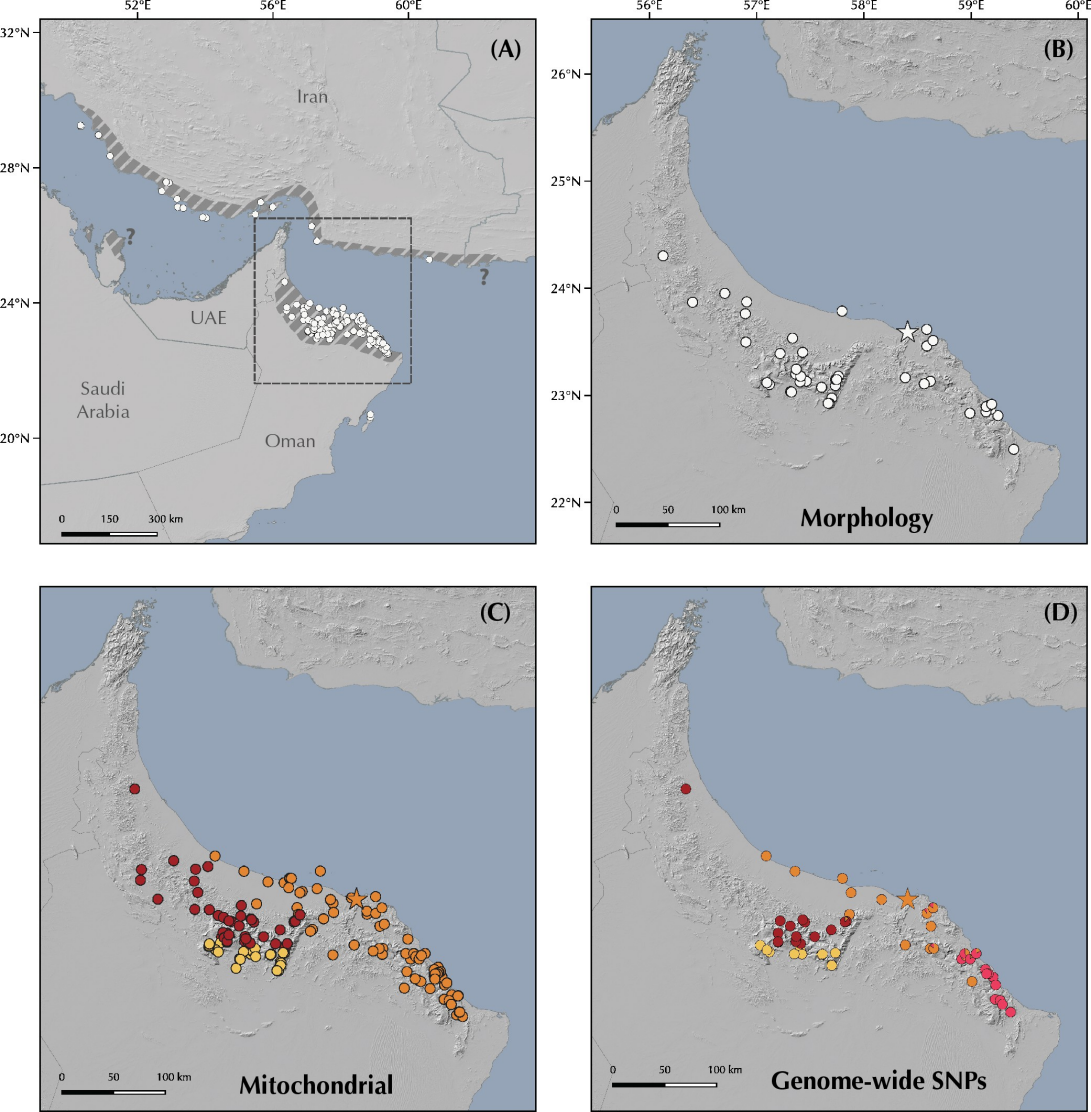
Distribution of *P*. *rupestris* sensu stricto, including **(A)** global distribution of the species including regions with introduced populations. *Pristurus rupestris* sensu lato in Qatar and its uncertain presence in West Pakistan have been highlighted as question marks; **(B)** Arabian specimens used for morphological multivariate analyses; **(C)** Arabian specimens used for sympatry analysis identified as *P*. *rupestris* by Garcia-Porta et al. (2017) using the 12S mitochondrial genetic marker; **(D)** Arabian specimens used for genome-wide SNP analyses, colored by the most supported ADMIXTURE configuration in Burriel-Carranza et al. (2024). Type locality for the species is represented with an orange star. Colors refer to the following populations: Yellow: candidate species 1 and 2 from Garcia-Porta et al. (2017) and *P*. *rupestris* BFD1 from Burriel-Carranza et al. (2024); Orange and pink: candidate species 3 from Garcia-Porta et al. (2017) and *P*. *rupestris* BFD2 from Burriel-Carranza et al. (2024); Dark red: candidate species 4.5 from Garcia-Porta et al. (2017) and *P*. *rupestris* BFD3 from Burriel-Carranza et al. (2024).

#### Pristurus rupestris

Arnold, 1977: 106 (part.); Arnold, 1986: 421 (part.); Arnold, 2009: 3 (part.); van deer Kooij, 2000: 116 (part.); Sindaco and Jeremčenko, 2008: 122 (part.); Gardner, 2013: 178 (part.); Badiane et al., 2014: 34 (part.); Šmíd et al., 2014:48; Šmíd et al., 2021: 1189; Tejero-Cicuendez et al., 2021: S1 Fig; Tejero-Cicuéndez et al., 2022: S1 Fig; Burriel-Carranza et al., 2022: 9; Burriel-Carranza et al., 2024: S1 Fig; Carranza et al., 2021: 119.

#### Pristurus rupestris rupestris

Schmidt 1952: 2 (part.); Badiane et al., 2014: 33 (part.); Saberi-Pirooz et al., 2019:1; Carranza et al., 2018: S2 Appendix, 61.

#### Pristurus rupestris iranicus

Schmidt 1952: 1; Wermuth 1965: 151; Badiane et al. 2014: 35.

*Pristurus rupestris rupestris* candidate species 1,2,3 and 4.5. Garcia-Porta et al., 2017: 5; Saberi-Pirooz et al., 2019: 11.

*Pristurus rupestris rupestris* Clade 4. Garcia-Porta et al., 2017: 5

*Pristurus rupestris* BFD1, BFD2 and BFD3. Burriel-Carranza et al., 2024: S24 Fig.

#### Lectotype

The taxonomic description of *Pristurus rupestris* Blanford, 1874 was based on two female syntype specimens collected by W.T. Blanford in 1872 and deposited inside the same jar at the collections of the NHMUK with catalogue numbers 1874.11.23.89–90 (NHMUK:ecatalogue:1885855). The two specimens can be differentiated based on their attached labels that indicate their different origin: Muscat, Oman and Kharg island, near Bushehr, Iran. Schmidt (1952) [73] restricted the type locality of *Pristurus rupestris* to Muscat, Oman and described the subspecies *Pristurus rupestris iranicus* Schmidt, 1952 restricted to the southern coast of Iran. This included some offshore islands based on a specimen from Bushehr, Iran collected in March 10, 1937 by E.W. Kaiser and deposited at the Natural History Museum of Denmark, University of Copenhagen with catalogue number NHMD876583. More recently, Saberi-Pirooz et al. (2019) [74] analyzed specimens across the distribution range of *P*. *r*. *iranicus* and showed that the Iranian subspecies branched within the deep lineage composed of specimens from the type locality of *P*. *r*. *rupestris* (Muscat, Oman), making *P*. *r*. *rupestris* paraphyletic. Considering these molecular results and the lack of morphological evidence, *P*. *r*. *iranicus* was synonymized with *P*. *rupestris* [74]. In order to avoid any future taxonomic confusion, and based on the terra typica restricta by Schmidt (1952) [73] to Muscat, Oman, we here formally designate the specimen from Muscat, Oman from the type series deposited at the NHMUK 1874.11.23.89 as the lectotype of *Pristurus rupestris*.

#### Other material examined

Additional specimens assigned to *Pristurus rupestris* sensu stricto used for morphological, genetic, genomic, and geographical analyses can be consulted in Table 1.

#### Diagnosis

*Pristurus rupestris* corresponds to a genetically highly distinct lineage of the *P*. *rupestris* species complex. Together with *Pristurus assareen* **sp. nov.** (*P*. *r*. *rupestris* candidate species 16 from Garcia-Porta et al. 2017; *P*. *rupestris* BFD12 in Burriel-Carranza et al. 2024; described below) [27, 28], it constitutes the youngest divergence within the species complex with an approximate date of origin of 3.6 mya (95% HPD: 2.9–4.4 mya). *Pristurus rupestris* is hereafter restricted to candidate species 1,2,3 and 4.5 from Garcia-Porta et al. (2017) [27] corresponding to putative species BFD1–BFD3 from Burriel-Carranza et al. (2024) [28], and by all Iranian *P*. *rupestris*, formerly classified as *P*. *r*. *iranicus* but later synonymized with *P*. *rupestris* [74]. This small species of *Pristurus* is characterized by the combination of the following characters: (1) a maximum recorded SVL of 27.4 mm; (2) 6–7 upper labial scales; (3) 3–4 lower labial scales; (4) 3–5 small postmental scales; (5) absence of mid-dorsal enlarged scales (crest) along the body; (6) laterally compressed tail usually with conspicuous dorsal and smaller ventral enlarged scales (crest) in males, and less conspicuous (sometimes absent) in females; (7) one unique mutation in the *rag2* alignment: a T instead of a C in position 276 (S1 Appendix).

#### Morphological, genetic and phylogeographic remarks

Multivariate morphological analyses show that *P*. *rupestris* sensu stricto shows a broad overlap in shape morphospace with every representative of the species complex. Procrustes ANOVA and subsequent pairwise comparisons on the Principal Components of the PCA (explaining almost 50% of all shape variance; Fig 3A and S2–S4 Tables) showed significant differences between *P*. *rupestris* sensu stricto and two species described herein that only occur at mid to high elevations (*P*. *ali* **sp. nov.**: corresponding to *P*. *r*. *rupestris* candidate species 6 and 7; *P*. *assareen* **sp. nov.**: corresponding to *P*. *r*. *rupestris* candidate species 16 in Garcia-Porta et al. 2017) [27]. However, no unique diagnostic morphological characters were found between *P*. *rupestris* sensu stricto and any other *Pristurus* species described herein. In addition, *Pristurus rupestris* sensu stricto is found in sympatry and even syntopy with every species of *Pristurus* described in this study (S7 Table), thus identification of this species relies on molecular methods.

All mitochondrial (Fig 4A), nuclear loci (Fig 4B), and genome-wide SNP analyses (Figs 3A, 3B, 5, S1 and S2) are fully concordant in the differentiation of *P*. *rupestris* sensu stricto as a distinct species from all other species described below. In all phylogenetic reconstructions, *P*. *rupestris* sensu stricto forms a clade together with *P*. *assareen* **sp. nov.** (corresponding to *P*. *r*. *rupestris* candidate species 16 sensu Garcia-Porta et al., 2017 [27] and *P*. *rupestris* BFD12 in Burriel-Carranza et al. 2024; described below) [28] representing the youngest split within the species complex, originating about 3.6 mya (Fig 5). The results of the nuclear networks for the *cmos*, *mc1r*, *rag1 and rag2* loci shown in Fig 4B, indicate that *P*. *rupestris* sensu stricto bears private alleles for some of these genes (not shared with any other species included in the present analysis). Although occurring in sympatry with all the species described herein in several localities, no signs of admixture were detected between *P*. *rupestris* sensu stricto and any other species *Pristurus* described below. Uncorrected *p*-distances with the mitochondrial *12S* gene as well as F_ST_ estimates with genome-wide SNP data show a strong interspecific genetic differentiation (*12S* estimates ranging from 9–22%; F_ST_ values range from 0.52–0.8; S5 and S6 Tables, respectively).

*Pristurus rupestris* sensu stricto (Fig 8) contains high levels of intraspecific diversity (*12S*: 4.7%; S5 Table; F_ST_ values: 0.37–0.53; Fig 6). Three well-defined populations were identified in both mitochondrial and genome-wide SNP analyses. Across the southern slope of the Central Hajars we find lineage 1.2 (comprised by lineages 1 and 2 *sensu* Garcia-Porta et al. 2017; *P*. *rupestris* BFD1 *sensu* Burriel-Carranza et al. 2024) [27, 28], representing the first split within the species about 2.1 mya (95% HPD: 1.7–2.6 mya); across the northern slope of the Central Hajars and extending West until almost reaching the border with the UAE it inhabits lineage 4 (comprised by lineage 4.5 *sensu* Garcia-Porta et al. 2017; *P*. *rupestris* BFD3 *sensu* Burriel-Carranza et al. 2024) [27, 28]; finally, in the Eastern Hajars and extending West through the coastal area of the Batinah Plain lineage 3 occurs. This population is conformed by a single mitochondrial lineage, but genome-wide SNPs support the existence of two populations within this lineage, with a contact zone of admixed specimens in the western region of the Eastern Hajars (Fig 9D).

**Lectotype description.** As outlined above, Blanford (1874) [75] based his original description of *P*. *rupestris* on material originating from Muscat, Oman (Fig 7B.1–7B.3) as well as Kharg Island (northwest off Bushehr) in Iran (Fig 7C.1–7C.3). In his designation of the type material no information on where and which type specimens had been finally deposited was stated by the author. Although Boulenger (1885) [76] provided a thorough description of Blanford’s type material, the description of the lectotype from Muscat, Oman is still needed since he offered a general description for several syntypes only, including material from Muscat, Oman, Kharg island, Iran, and Socotra, Yemen (corresponding to *Pristurus sokotranus* Parker, 1938), together with measurements from a single specimen that was not identified therein [76]. After examining the available type material, we confirmed that the measurements provided by Boulenger (1885) [76] are from the female lectotype specimen from Muscat, Oman and therefore, we will use these metrics in the following description of the lectotype of *Pristurus rupestris* sensu stricto based on NHMUK1874.11.23.89–90 (NHMUK:ecatalogue:1885855), an adult female specimen with attached locality tag referring to Muscat, Oman. Specimen ventrally damaged with internal tissue apparent in the abdomen near the pelvic girdle and with dorsal skin partially wrinkled; tail missing. Body length 23 mm, head length 9 mm, and head width 5.5 mm. Moderately built, round body; limbs slender. Head robust and does not rise very steeply in profile, snout slightly blunt. Supranasals are separated into two scales. Scales on snout granular, polygonal and slightly pointed upwards. Scales on snout, anterior to eyes, are larger than those on top of head. Palpebral fold edged anteriorly with large scales and with pointed (ciliate) scales. Ear opening ellipsoidal. Upper labials (right/left) 6/6. Lower labials (right/left) 4/4, the fourth decreasing in size from the three large anterior scales. Four enlarged scales running backwards along the proximal borders of the third lower labials to end at about the level of the angle of the mouth. Mental large, wedge-shaped, truncated posteriorly by three postmental scales. Gulars small, rather pointed posteriorly, becoming slightly imbricate and bigger in size on throat. Dorsal scales on body small, polygonal and homogeneous, slightly pointed posteriorly. Ventrals flat and distinctly imbricate. Limbs slender. Scales on dorsal and anterior surface of the forelimbs slightly larger than dorsal body scales, flat and imbricate, fairly pointed. Scales beneath the upper forelimbs granular and smaller than ventral body scales; those beneath lower forelimbs large, flat, imbricate, and polygonal, larger than ventral body scales. Claws relatively shallow and weakly recurved. Scales on dorsal and anterior surface of hindlimbs large, flat and imbricate, fairly pointed. Scales beneath the upper hindlimbs granular and smaller than ventral body scales, those beneath lower hindlimbs large, flat, imbricate and polygonal, larger than ventral body scales.

Coloration in alcohol is grayish brown dorsally; faint thin dorsal midline pale grayish. Dorsally and both left and right of the midline irregularly-shaped whitish blotches present. Head presents the same color as the body with a broad dark brownish stripe running from the nostril through the eye and then dorsolaterally until above the ear opening. Faint large dark blotches are located from the gular region to the insertion of the hindlimbs ventrally, decreasing in number and brightness posteriorly. Ventral background coloration is grayish-whitish. Limbs banded dorsally with dark gray, light gray bands reaching until toes. Ventral coloration of limbs is grayish, darker than the abdomen and not banded.

#### Distribution

In the Arabian Peninsula, *P*. *rupestris* sensu stricto is now restricted to the Omani Hajar Mountains, spanning from Ra’s al Hadd to 40 km North of the Wadi Jizzi gap (almost reaching the UAE border; Fig 9). It has been recorded from sea level up to 2,115 m asl in Jabal Shams, Central Hajars, Oman. Some specimens from lineage 3 (candidate species 3 in Garcia-Porta et al. 2017; *P*. *rupestris* BFD2 in Burriel-Carranza et al., 2024) [27, 28] and lineage 4 (candidate species 4.5 in Garcia-Porta et al. 2017; *P*. *rupestris* BFD3 in Burriel-Carranza et al., 2024) [27, 28] have been recently discovered in Masirah Island and most probably are the result of human introduction (*work in progress*). The designation of morphologically typical *P*. *rupestris* sensu lato introduced populations from Qatar, Bahrain, eastern Saudi Arabia and the northern UAE coast, from Sharjah to Abu Dhabi and offshore islands, will need to be genetically reassessed in the light of the here presented taxonomic revision to corroborate their identity. Since it was shown that the Iranian *P*. rupestris genetically fall within a clade of specimens close to the type locality in Oman [74], they are here considered as *P*. *rupestris* sensu stricto. Therefore, outside the Arabian Peninsula, *P*. *rupestris* sensu stricto is found throughout the coast of Iran, reaching North to Bushehr, in Kharg island, and spreading south until almost reaching Pakistan [77, 78]. *Pristurus rupestris* sensu stricto has been reported marginally from southwestern coastal Pakistan adjacent to the Iranian border [19], but its presence in Pakistan is considered uncertain by Kamali (2020) [78], and it is not included in a recent checklist provided for Pakistani reptiles by Khan (2016) [77].

#### Habits

They are usually found on rocks, boulders, tree trunks, and human-made structures. Females lay single hard-shelled eggs throughout the year. They are mainly diurnal (although they can be active after sunset) and perform complex signaling including curling their tail up over the back, push-ups and inflating and laterally compressing their body and throat.

#### Conservation

*Pristurus rupestris* sensu stricto should be listed as Least Concern in view of its large distribution within Oman, its abundance, and because it is unlikely to be declining fast enough to qualify for listing in a threatened category.

### *Pristurus omanensis* Burriel-Carranza, Koppetsch, Garcia-Porta, Carranza–sp. nov. urn:lsid:zoobank.org:act:512E2CD2-6F46-445E-AA3E-D1288709AD74

(Figs 2–6, 10–13, S1, S2, S3 and Tables 1 and S1–S7; Alignment of phased haplotypes for the molecular diagnostic characters based on the *mc1r*, *cmos*, *rag1* and *rag2* genes can be found on S1 Appendix).

English name: Oman Semaphore Gecko

**Fig 10:**
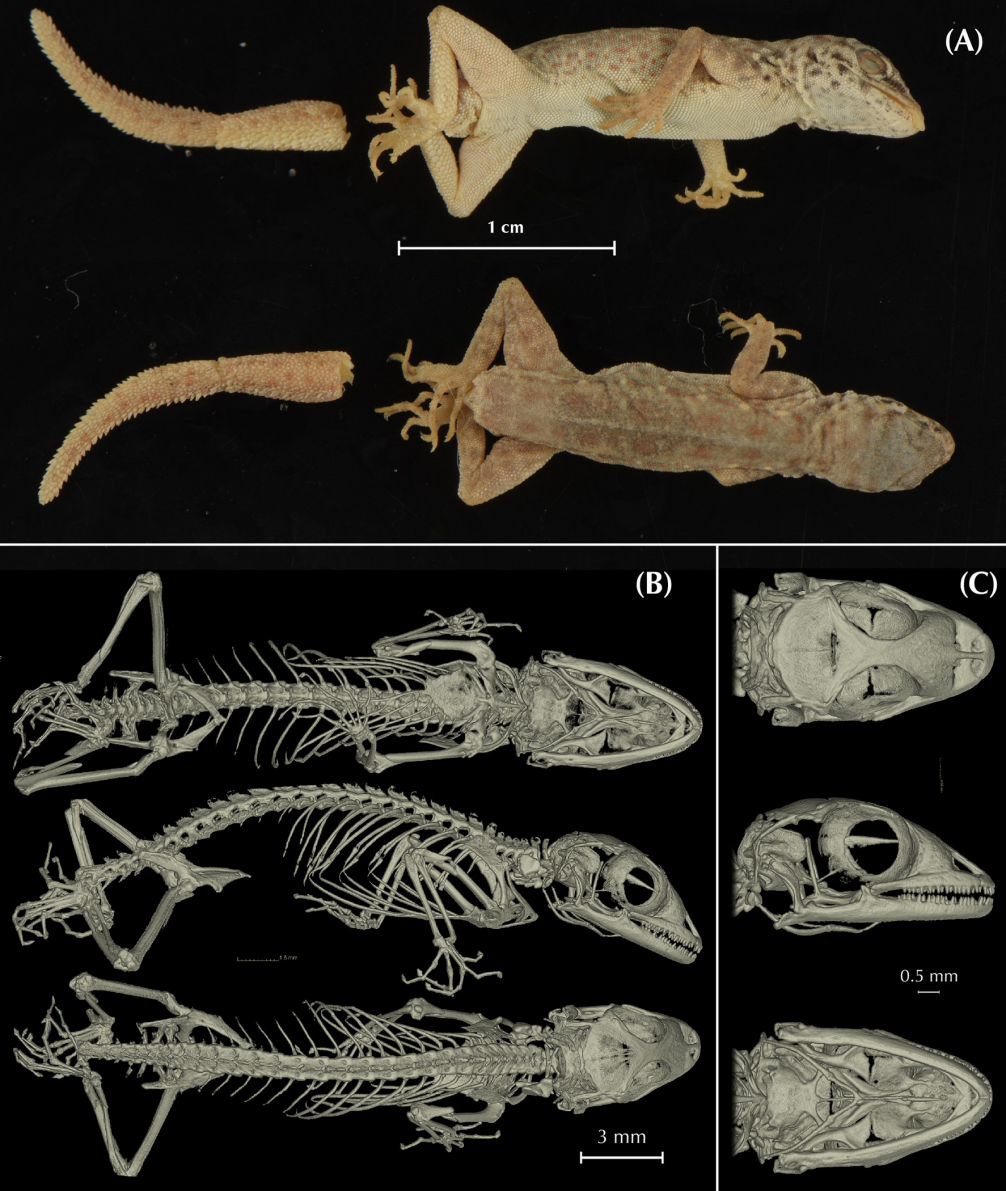
External and internal morphology of the *Pristurus omanensis* sp. nov. holotype specimen ZFMK104096 (sample code S8000). **(A)** Ventral-lateral and dorsal external view of the specimen; **(B)** Ventral, lateral and dorsal internal view obtained through μ-CT scanning; **(C)** close up of head bone structures of the holotype specimen ZFMK104096 (sample code S8000).

**Fig 11:**
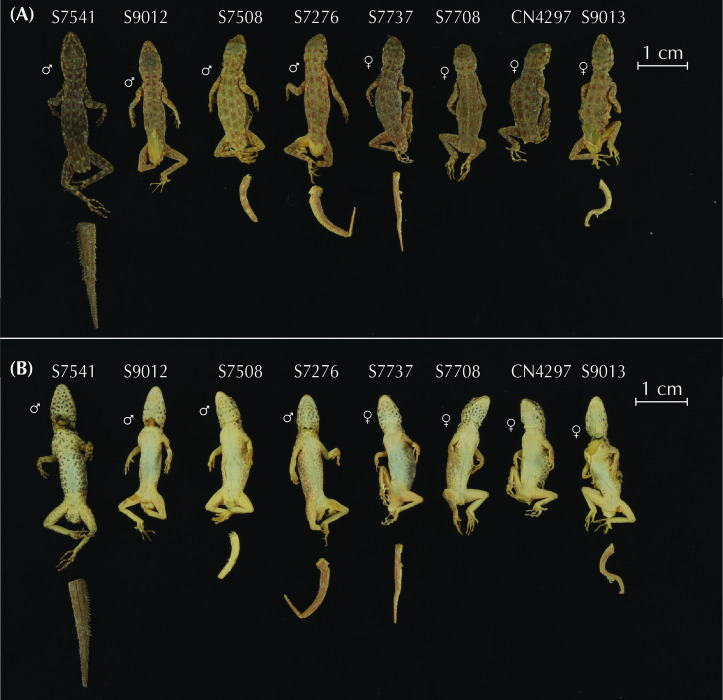
**(A)** Dorsal and **(B)** ventral view of *Pristurus omanensis* **sp. nov.** paratype specimens showing color variation. All specimens correspond to specimens from the type locality or the Eastern Hajars assigned to genomic lineage BFD5. Further variation in specimens of *P*. *omanensis* **sp. nov.** lineage BFD6 is shown in S3 Fig.

**Fig 12:**
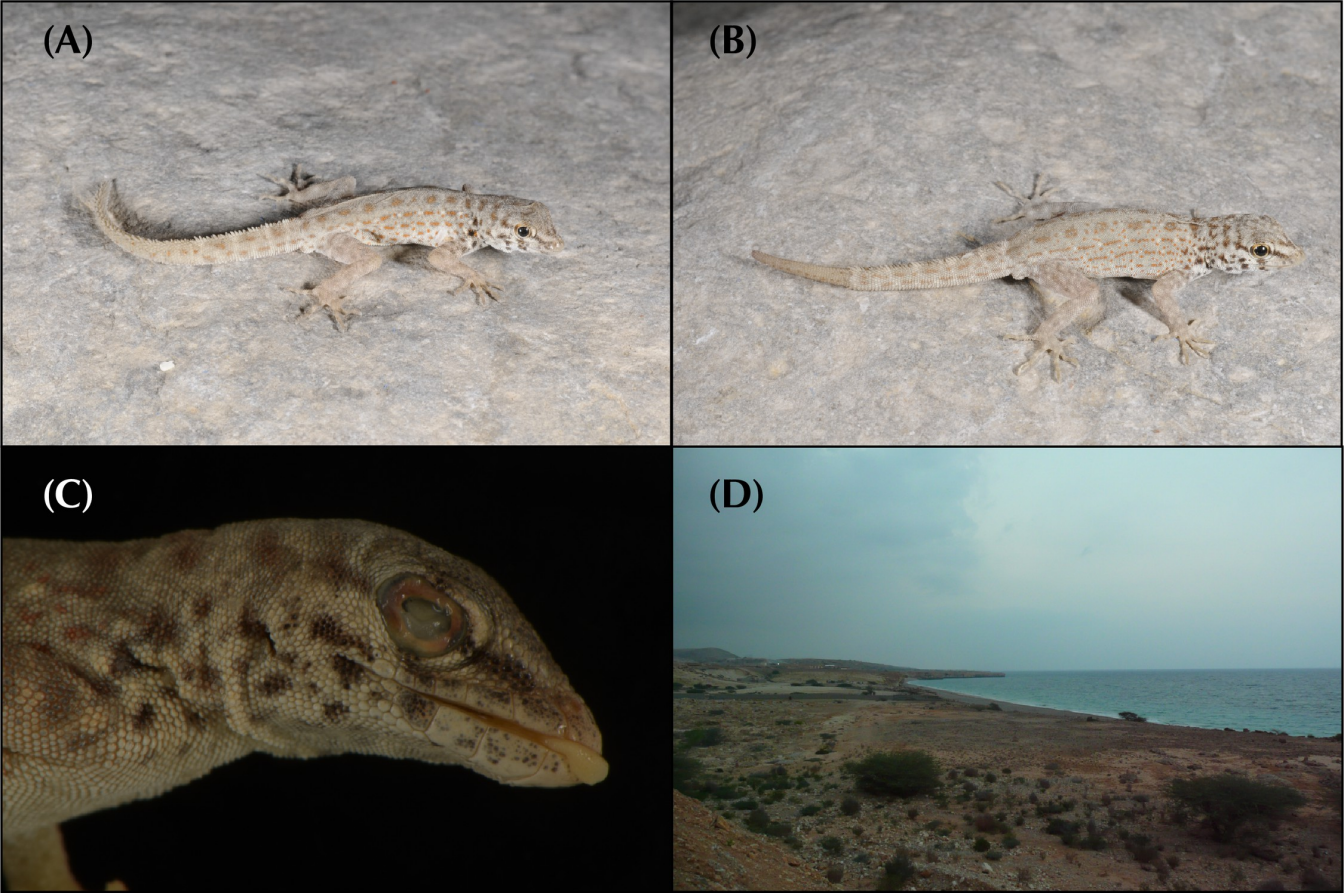
**(A)** *P*. *omanensis* **sp. nov.** male specimen from lineage 8 sensu GarciaPorta et al. (2017) (lineage BFD5 in Burriel-Carranza et al. 2024) with pale coloration, and a conspicuous dorsal crest in tail (picture code: *clade8_SC42471*); **(B)** *P*. *omanensis* **sp. nov.** male specimen from lineage 9 sensu GarciaPorta et al. (2017) (lineage BFD6 in Burriel-Carranza et al. 2024) from the Central Hajars; **(C)** Head close-up of the *P*. *omanensis* **sp. nov.** holotype (ZFMK104096; sample code S8000); **(D)** Habitat of *P*. *omanensis* **sp. nov.** in Tiwi beach, Oman, (type locality of the species). Pictures taken by S.C. (A,B,D) and T.K (C).

**Fig 13:**
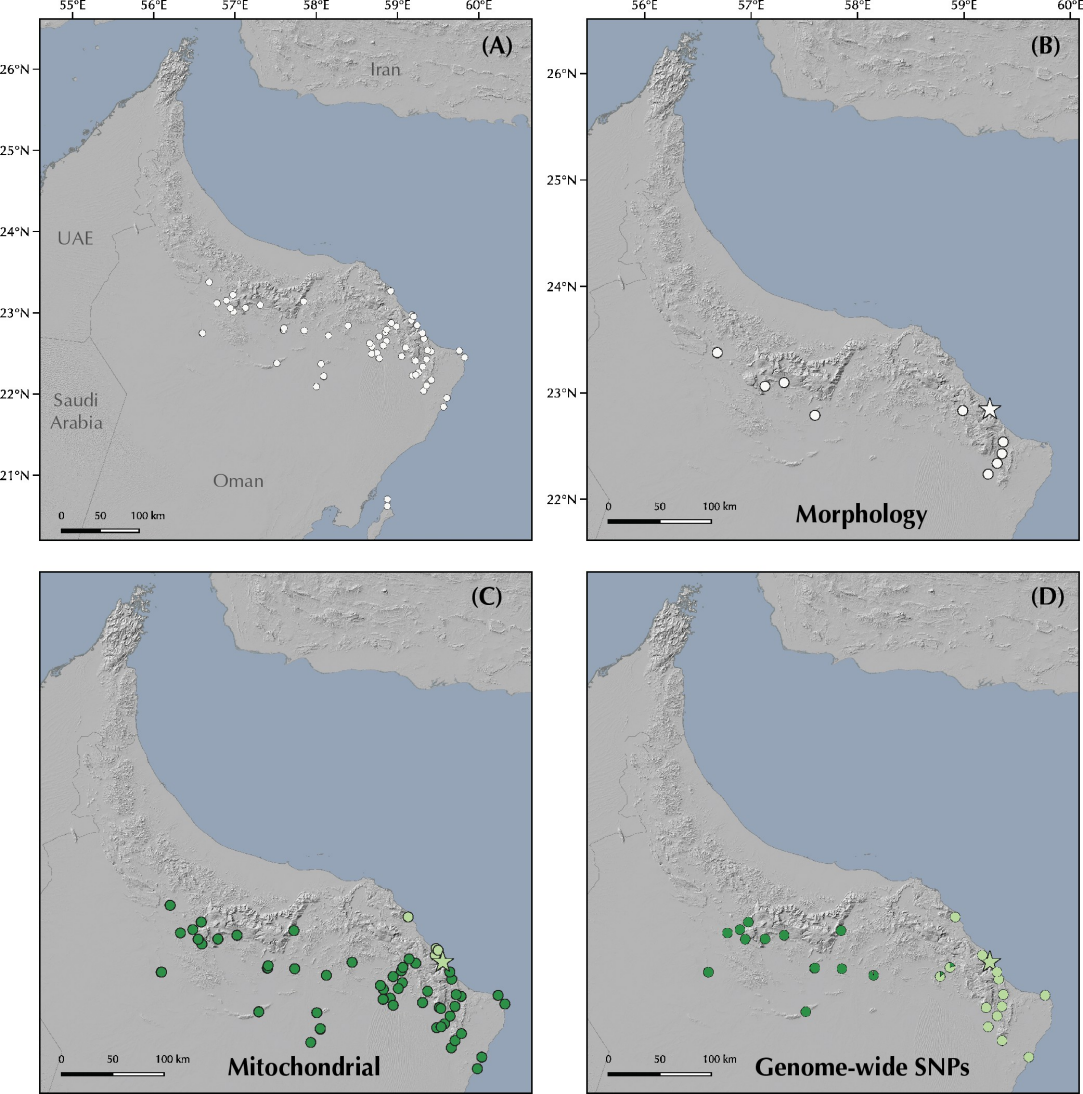
Distribution of *P*. *omanensis* **sp. nov.** in Arabia, including **(A)** all known populations (including an introduction to Masirah island); **(B)** specimens used for morphological multivariate analyses; **(C)** specimens used for sympatry analysis identified as *P*. *rupestris* candidate species 8 and 9 by Garcia-Porta et al. (2017) using the 12S mitochondrial genetic marker; **(D)** specimens used for genome-wide SNP analyses colored by the most supported ADMIXTURE configuration in Burriel-Carranza et al. (2024). Type locality for the species is represented with a light green or white star. Colors refer to the following populations: Light green: candidate species 8 from Garcia-Porta et al. (2017) and *P*. *rupestris* BFD5 from Burriel-Carranza et al. (2024); Dark green: candidate species 9 from Garcia-Porta et al. (2017) and *P*. *rupestris* BFD6 from Burriel-Carranza et al. (2024).

#### Pristurus rupestris

Arnold, 1977: 106 (part.); Arnold, 1986: 421 (part.); Arnold, 2009: 3 (part.); van deer Kooij, 2000: 116 (part.); Sindaco and Jeremčenko, 2008: 122 (part.); Gardner, 2013: 178 (part.); Badiane et al., 2014: 34 (part.).

*Pristurus rupestris rupestris* candidate species 8 and 9. Garcia-Porta et al., 2017: 5.

*Pristurus rupestris rupestris* Clade 1. Garcia-Porta et al., 2017: 5; Saberi-Pirooz et al., 2019:11.

*Pristurus* sp5. Šmíd et al., 2021: 1189; Tejero-Cicuendez et al., 2021: S1 Fig; Tejero-Cicuéndez et al., 2022: S1 Fig; Burriel-Carranza et al., 2022: 9; Burriel-Carranza et al., 2024: S1 Fig; Carranza et al., 2018: S2 Appendix, 66; Carranza et al., 2021: 119.

*Pristurus rupestris* SP8 and SP9. Saberi-Pirooz et al., 2019:11.

*Pristurus rupestris* BFD5 and BFD6. Burriel-Carranza et al., 2024: S24 Fig.

#### Holotype

ZFMK 104096 (sample code S8000), adult male from Tiwi, 12 m asl, Eastern Hajars (North Oman), 22.84441’N, 59.24189’E, WGS84, collected in November 2010 by J. Garcia-Porta (assigned to *Pristurus rupestris rupestris* candidate species 8 from Garcia-Porta et al. 2017 and to *Pristurus rupestris* BFD5 from Burriel-Carranza et al. 2024; see Table 1).

#### Paratypes

NHMOK 2657 (sample code S7541), adult male from Sumayyan road, 1,005 m asl, Eastern Hajars (North Oman), 22.83326’N, 58.98821’E, WGS84, collected in May 2011 by S. Carranza, F. Amat and E. Gómez-Díaz (assigned to *P*. *r*. *rupestris* candidate species 9 from Garcia-Porta et al. 2017 and to *Pristurus rupestris* BFD5 from Burriel-Carranza et al. 2024; see Table 1) [27, 28]; MZB 2024–0988 (sample code S9012; MorphoBank M849884–M849886), adult male from Fulayj, 132 m asl, Eastern Hajars (North Oman), 22.42828’N, 59.35618’E, WGS84, collected in May 2011 by S. Carranza, F. Amat and E. Gómez-Díaz (assigned to *P*. *r*. *rupestris* candidate species 9 from Garcia-Porta et al. 2017 and to *Pristurus rupestris* BFD5 from Burriel-Carranza et al. 2024; see Table 1) [27, 28]; IBES7508 (sample code S7508; MorphoBank M849878–M849882), adult male from Tiwi, 28 m asl, Eastern Hajars (North Oman), 22.8448’N, 59.24156’E, WGS84, collected in May 2011 by S. Carranza, F. Amat and E. Gómez-Díaz (assigned to *P*. *r*. *rupestris* candidate species 8 from Garcia-Porta et al. 2017 and to *Pristurus rupestris* BFD5 from Burriel-Carranza et al. 2024; see Table 1) [27, 28]; IBES7276 (sample code S7276; MorphoBank M849893–M849896), adult male from Fulayj, 212 m asl, Eastern Hajars (North Oman), 22.33769’N, 59.31128’E, WGS84, collected in May 2011 by S. Carranza, F. Amat and E. Gómez-Díaz (assigned to *P*. *r*. *rupestris* candidate species 9 from Garcia-Porta et al. 2017 and to *Pristurus rupestris* BFD5 from Burriel-Carranza et al. 2024; see Table 1) [27, 28]; MZB 2024–0987 (sample code S7737; MorphoBank M849864–M849867), adult female from Ashila, 42 m asl, Eastern Hajars (North Oman), 21.95181’N, 59.6082’E, WGS84, collected in October 2010 by S. Carranza and F. Amat (assigned to *P*. *r*. *rupestris* candidate species 9 from Garcia-Porta et al. 2017 and to *Pristurus rupestris* BFD5 from Burriel-Carranza et al. 2024; see Table 1) [27, 28]; IBES7708 (sample code S7708; MorphoBank M849869–M849871), adult female from Al Ashkharah, 13 m asl, Eastern Hajars (North Oman), 21.84242’N, 59.56673’E, WGS84, collected in October 2010 by S. Carranza and F. Amat (assigned *P*. *r*. *rupestris* candidate species 9 from Garcia-Porta et al. 2017 and to *Pristurus rupestris* BFD5 from Burriel-Carranza et al. 2024; see Table 1) [27, 28]; IBECN4297 (sample code CN4297; MorphoBank M849873–M849876), adult female near Wadi Naam, 457 m asl, Eastern Hajars (North Oman), 22.70851’N, 58.77569’E, WGS84, collected in May 2013 by S. Carranza, F. Amat, M. Metallinou, M. Simó-Riudalbas and P. de Pous (assigned to *P*. *r*. *rupestris* candidate species 9 from Garcia-Porta et al. 2017 and to *Pristurus rupestris* BFD5 from Burriel-Carranza et al. 2024; see Table 1) [27, 28]; IBES9013 (sample code S9013; MorphoBank M849888–M849891), adult female from Wadd, 136 m asl, Eastern Hajars (North Oman), 22.53899’N, 59.36823’E, WGS84, collected in May 2011 by S. Carranza, F. Amat and E. Gómez-Díaz (assigned to *P*. *r*. *rupestris* candidate species 9 from Garcia-Porta et al. 2017 and to *Pristurus rupestris* BFD5 from Burriel-Carranza et al. 2024; see Table 1) [27, 28].

#### Other material examined

Additional *Pristurus omanensis* **sp. nov.** specimens formerly assigned to *P*. *r*. *rupestris* candidate species 8 and 9 from Garcia-Porta et al. (2017) [27] and *Pristurus rupestris* BFD5 and BFD6 from Burriel-Carranza et al. (2024) [28] used for morphological, genetic, genomic, and geographical analyses are found in Table 1.

#### Etymology

The species epithet ‘*omanensis*’ is an adjective that refers to the distribution range of the species, restricted to the Omani side of the Al Hajar Mountain range.

#### Diagnosis

*Pristurus omanensis* **sp. nov.** corresponds to a genetically highly distinct lineage of the *Pristurus rupestris* species complex *sensu* Garcia-Porta et al. (2017) [27] from the Central and Eastern Hajar Mountains (North Oman). It represents the oldest split within the *Pristurus rupestris* species complex, having diverged from all other species within the group around 10.8 mya (95% HPD: 8.7–12.6 mya; Fig 5). This small species of *Pristurus* is characterized by the combination of the following characters: (1) a maximum recorded SVL of 25.8 mm; (2) 6–8 upper labial scales; (3) 4–5 lower labial scales; (4) 3–5 small postmental scales; (5) absence of mid-dorsal enlarged scales (crest) on the body; (6) laterally compressed tail usually with conspicuous dorsal and smaller ventral enlarged scales (crest) in males, and less conspicuous (sometimes absent) in females; (7) four unique mutations in the *mc1r* alignment: A instead of G in position 45; T instead of C in position 438; A instead of G in position 443; T instead of G in position 510; (8) one unique mutation in the *cmos* alignment: A instead of G in position 260; (9) two unique mutations in the *rag1* alignment: T instead of C in position 205; G instead of A in position 242; (10) four unique mutations in the *rag2* alignment: A instead of G in position 17; G instead of A in position 61; G instead of A in position 67; A instead of C in position 348 (see S1 Appendix).

#### Morphological, genetic and phylogeographic remarks

Multivariate morphological analyses show that *P*. *omanensis* **sp. nov.** presents some degree of morphospace overlap with every representative from the species complex (Fig 3A). However, while it is not significantly distinct from *P*. *rupestris* sensu stricto and a *Pristurus feulneri* **sp. nov.** (corresponding to Clade 3 in Garcia-Porta et al., 2017 and described below) [27], it is significantly distinct from the other two species that only occur at mid to high elevations (*P*. *ali* **sp. nov.** and *P*. *assareen* **sp. nov.** which correspond to Clade 2 and candidate species 16 in Garcia-Porta et al. 2017; S2–S4 Tables, respectively; species described below) [27]. *Pristurus omanensis* **sp. nov.** is monophyletic in both mitochondrial and nuclear phylogenies (Figs 4A, 5 and S1). According to the analysis of divergence time estimation inferred with *dataset4*, this species diverged from all other relatives within the *Pristurus rupestris* species complex about 10.3 mya (95% HPD: 8.7–12.41 mya; Fig 5). The results of the nuclear networks shown in Fig 4B indicate that all alleles of *P*. *omanensis* **sp. nov.** for all independent loci analyzed (*mc1r*, *cmos*, *rag1* and *rag2*) are private (not shared with any other species included in the present study). *Pristurus omanensis* **sp. nov.** is the species with the greatest genetic differentiation within the *Pristurus rupestris* species complex, with 21–24.5% interspecific distance in the mitochondrial *12S* gene (S5 Table) and a genome-wide fixation index above 0.8 with all other *Pristurus* species (Fig 6). Moreover, while *Pristurus rupestris* sensu stricto and the other species described herein are usually found throughout the entire elevation gradient (or from mid to high elevations), *P*. *omanensis* **sp. nov.** seems to be restricted to low elevations, rarely exceeding 1,000 m asl (only one record from a total of 102 records; Table 1). Even though *Pristurus omanensis* **sp. nov.** is found in sympatry at less than 100 m with *Pristurus rupestris* sensu stricto and with another *Pristurus* species described below (S7 Table), no evidence of gene flow has been found between these species (Fig 3B and 3C; Burriel-Carranza et al. 2023: S38 Fig) [28].

*Pristurus omanensis* **sp. nov.** (Fig 12) presents some intraspecific diversity with 2.9% of genetic distance in the mitochondrial gene *12S* (S5 Table) and an F_ST_ value of 0.25 between the populations that comprise this species (Fig 6). Phylogenetic (Fig 4A), phylogenomic (Figs 5 and S1) and population genomic analyses (Fig 3B and 3C) support the presence of two independent parapatric lineages within *P*. *omanenis* **sp. nov.**. However, mitochondrial and genome-wide nuclear SNPs disagree in the topology and composition of such lineages. Mitochondrial data recover an isolated clade, restricted to the northeasternmost distribution of *P*. *omanensis* **sp. nov.** (referred to *Pristurus rupestris rupestris* candidate species 8 in Garcia-Porta et al. 2017) [27], while the other lineage is comprised by specimens from both the Eastern and Central Hajars (referred to *Pristurus rupestris rupestris* candidate species 9 in Garcia-Porta et al. 2017; Figs 4A and 13C) [27]. On the other hand, genome-wide nuclear data recover all specimens from the Eastern Hajars as a unique evolutionary unit, sister to the specimens inhabiting the Central Hajars, with a hybrid contact zone with admixed specimens where both populations overlap in distribution (referred to *P*. *rupestris* putative species BFD5 and BFD6 in Burriel-Carranza et al. 2024, Figs 3C, 13D and S1) [28]. Such mito-nuclear discordance between specimens inhabiting the Central and Eastern Hajars is not uncommon in geckos (see Burriel-Carranza et al. 2023c for a similar example in *Trachydactylus hajarensis*) [40], and since the mitochondrial genome is maternally inherited, it could be representative of dispersal differences between males and females. Given that gene flow exists, that admixed specimens are observed in nature and, together with the lack of support for species status in the MSC species delimitation approach implemented by means of BPP and *gdi* (Figs 5 and S1), we believe that the two lineages within *P*. *omanensis* **sp. nov.** reflect intraspecific diversity and should be considered as distinct populations of the same species.

#### Description of the holotype

ZFMK 104096 (sample code S8000) (Fig 10). Adult male. Complete specimen, with the tail detached. Data on nine morphometric and two pholidotic variables (see Materials and Methods) are provided in S1 Table. SVL 22.2 mm, head length 28.8% of SVL, and head width 59% of head length. Moderately built, round body, habitus comparable to *P*. *rupestris* sensu stricto; limbs and tail not especially slender. Head robust and does not rise very steeply in profile, snout slightly blunt. Nostril separated from the rostral and situated between three scales: a wide supranasal extending to the midline margin, a small upper postnasal and a large lower postnasal extending laterally along the lower edge. Supranasals are separated into two scales. Scales on snout granular, polygonal and slightly pointed upwards. About nine scales in a straight line from the lower postnasal to the anterior edge of the orbit. Scales on snout, anterior to eyes, are larger than those on top of head. Around 24 scales across mid-orbital region. Palpebral fold edged anteriorly with large scales and not edged with pointed (ciliate) scales. Ear opening ellipsoidal. Upper labials (right/left) 7/7. Lower labials (right/left) 4/4, the fourth decreasing in size from the three large anterior scales. Four enlarged scales running backwards along the proximal border of the third lower labial to end at about the level of the angle of the mouth. Mental large, wedge-shaped, truncated posteriorly by five gular scales. Gulars small, rather pointed posteriorly, becoming slightly imbricate on throat. Dorsal scales on body small, polygonal and homogeneous, slightly pointed posteriorly. Ventrals flat and distinctly imbricate. Scales on dorsal and anterior surface of the forelimbs slightly larger than dorsal body scales, flat and imbricate, fairly pointed. Scales beneath the upper forelimbs granular and smaller than ventral body scales; those beneath lower forelimbs large, flat, imbricate and polygonal, larger than ventral body scales. Claws relatively shallow and weakly recurved. Scales on dorsal and anterior surface of hindlimbs large, flat and imbricate, fairly pointed. Scales beneath the upper hindlimbs granular and smaller than ventral body scales, those beneath lower hindlimbs large, flat, imbricate and polygonal, larger than ventral body scales. Regenerated tail relatively short and robust, anteriorly more or less cylindrical, posteriorly slightly compressed. Ridges of strongly elongated scales present, low crest on midline and on lower midline of tail is made up of a longitudinal row of enlarged scales, more enlarged dorsally. Tail scales large, flat and imbricate, pointed posteriorly; dorsals and ventrals considerably larger than those on the body.

Coloration in alcohol is grayish brown dorsally; broad dorsal midline pale grayish. Dorsally and both left and right of the midline irregularly shaped reddish blotches present. Dorsolaterally thin discontinuous pale reddish lines that dissolve into irregularly distributed spots on the flanks. Whitish spots are distributed in transversal rows laterally, and double the diameter of the reddish lines. Head is the same color as the body with a broad dark brownish stripe running from the nostril through the eye and then dorsolaterally until above the ear opening. Single large dark blotches are located from beneath the eye to the entire gular region reaching posteriorly to the insertion of the forelimbs. Larger and more prominent blotches are distributed laterally on the throat decreasing in brightness ventrally. Ventral background coloration is creamish-whitish. Limbs pale beige dorsally, toes not banded. Tail similarly colored like dorsals, with pale reddish spots scattered.

The figured μ-CT-scanned specimen (Fig 10B) has a maximal skull length of 6.7 mm, maximal height of 3.2 mm and maximal width of 4.0 mm. 25 presacral vertebrae. Noteworthy minor qualitative cranial osteological differences (Fig 10C) to the other holotype specimens of the *Pristurus rupestris* species complex are: Vomer posteriorly rounded and bordered by a heart-shaped opening; osseous naris edge sharp and almost right angled; premaxilla and nasal steep to frontal region.

#### Variation

Data on nine morphometric and two pholidotic variables (see Materials and Methods) for all eight paratypes (see above) are provided in S1 Table. Largest male 25.4 mm SVL; largest female 22.6 mm SVL. All paratypes have broken or incomplete tails; four of eight have the throat sliced, three of eight individuals show damaged skin. Paratype NHMOK 2657 (sample code S7541) has a regenerated tail. Dorsal coloration ranges from pale beige to dark gray, with thin or absent pale grayish dorsal midlines. Dorsal pattern consisting of incomplete thin reddish lines to reddish blotches, bordered by similar-sized pale whitish spots. Laterally small reddish spots present in all paratypes, in some individuals also present ventrolaterally. Few to multiple large gular blotches present in all paratypes (Fig 11).

#### Distribution

*Pristurus omanensis* **sp. nov.** is endemic to Oman. It is only found at low elevations surrounding the southern areas of the Central Hajars, and surrounding the southeastern region of the Eastern Hajars up until Ras al Hadd and then entering North until Qurayyat, Oman. Despite several extensive surveys, it has never been found in the Western Hajars or in the northern slopes of the Central Hajars. It has been recorded from 0 m asl up to 1,005 m asl, although it is rarely found above 700 m asl (only one record out of 102).

#### Habits

*P*. *omanensis* **sp. nov.** is a diurnal species, although it can also be seen active after sunset. It is abundant on rocks, boulders, tree trunks, and human-made structures such as walls, houses, and gardens. It performs complex signaling including push-ups, inflating and laterally compressing the body and throat, and curling and waving the tail up over the back. It preys on ants and other arthropods using a sit-and-wait strategy. Females lay single hard-shelled eggs throughout the year.

#### Conservation

This species is endemic to the Hajar Mountain range in Oman. Whilst the area of occupancy (AOO) is restricted, the species should be assessed as Least Concern in view of its presumed large and stable population, occurrence in more than ten locations, and absence of continuing decline in AOO, extent of occurrence (EOO), or in habitat area or quality and extent.

### *Pristurus ali* Burriel-Carranza, Koppetsch, Garcia-Porta, Carranza–sp. nov. urn:lsid:zoobank.org:act:C3D2FA38-8C9A-48E5-9A5E-668AAFD1A830

(Figs 2–6, 14–17, S1, S2 and S4 and Tables 1 and S1–S7; Alignment of phased haplotypes for the molecular diagnostic characters based on the *mc1r*, *cmos*, *rag1* and *rag2* genes can be found on S1 Appendix).

English name: Ali’s Semaphore Gecko

**Fig 14:**
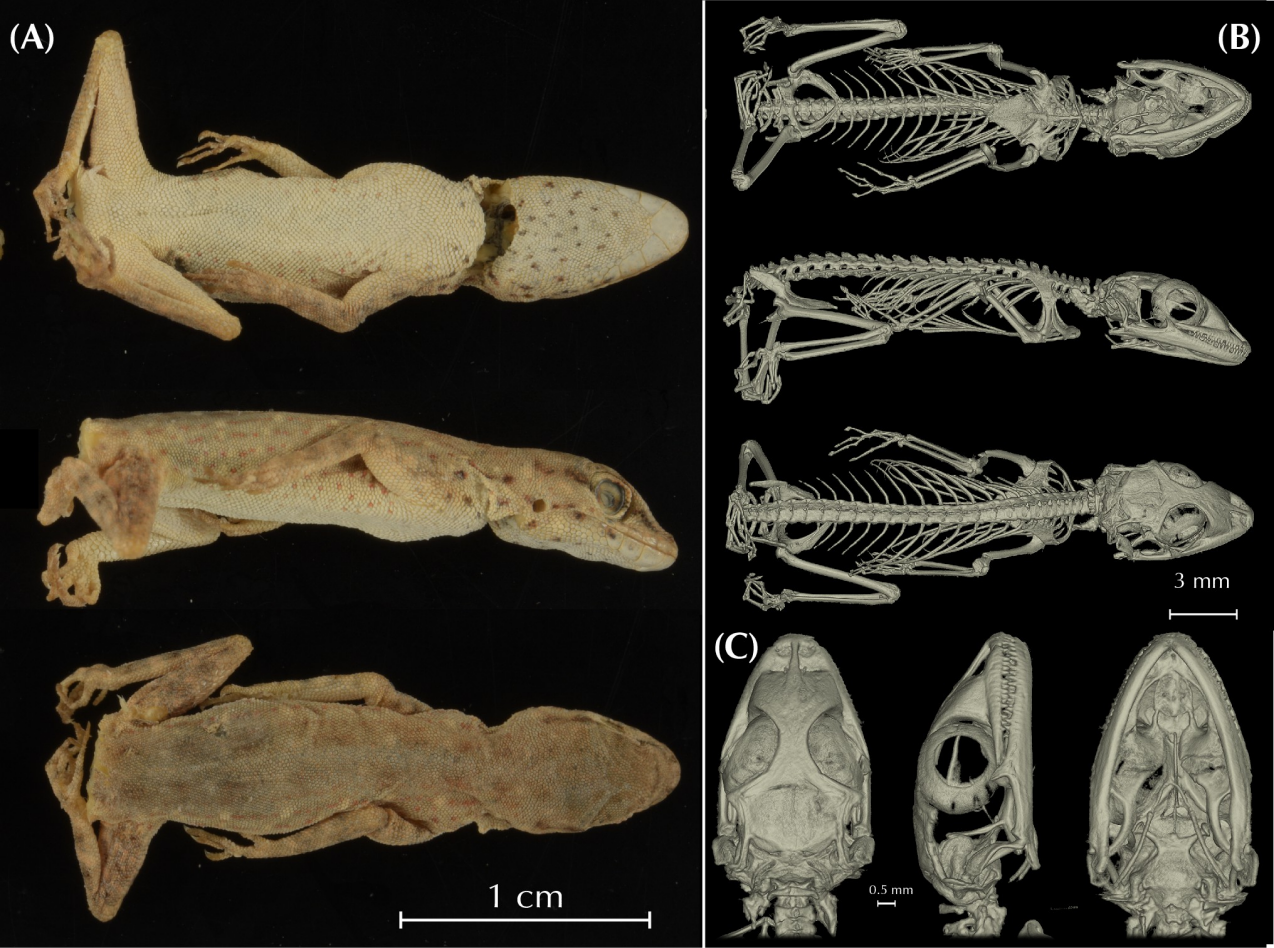
External and internal morphology of *Pristurus ali* sp. nov. holotype specimen ZFMK104093 (sample code S7547). **(A)** Ventral, lateral and dorsal external view of the specimen; **(B)** Ventral, lateral and dorsal internal view obtained through μ-CT scanning; **(C)** close up of head bone structures of the holotype specimen ZFMK104093 (sample code S7547).

**Fig 15:**
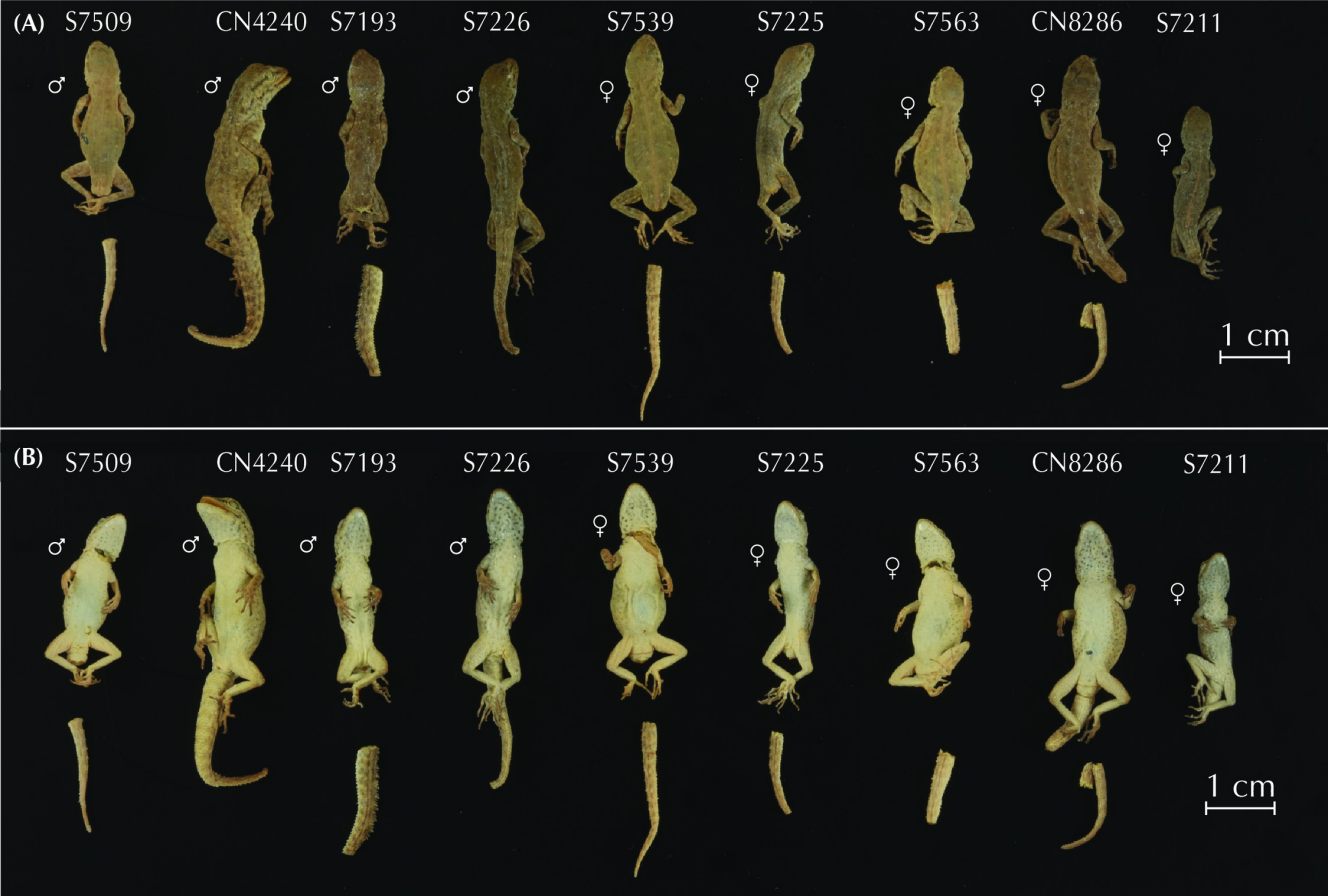
**(A)** Dorsal and **(B)** ventral view of *Pristurus ali* **sp. nov.** paratype specimens showing color variation. All specimens correspond to specimens from the type genetic lineage 7 *sensu* Garcia-Porta et al. (2017). Further variation in specimens of *P*. *ali* **sp.nov.** lineage 6 *sensu* Garcia-Porta et al. (2017) can be consulted in S4 Fig.

**Fig 16:**
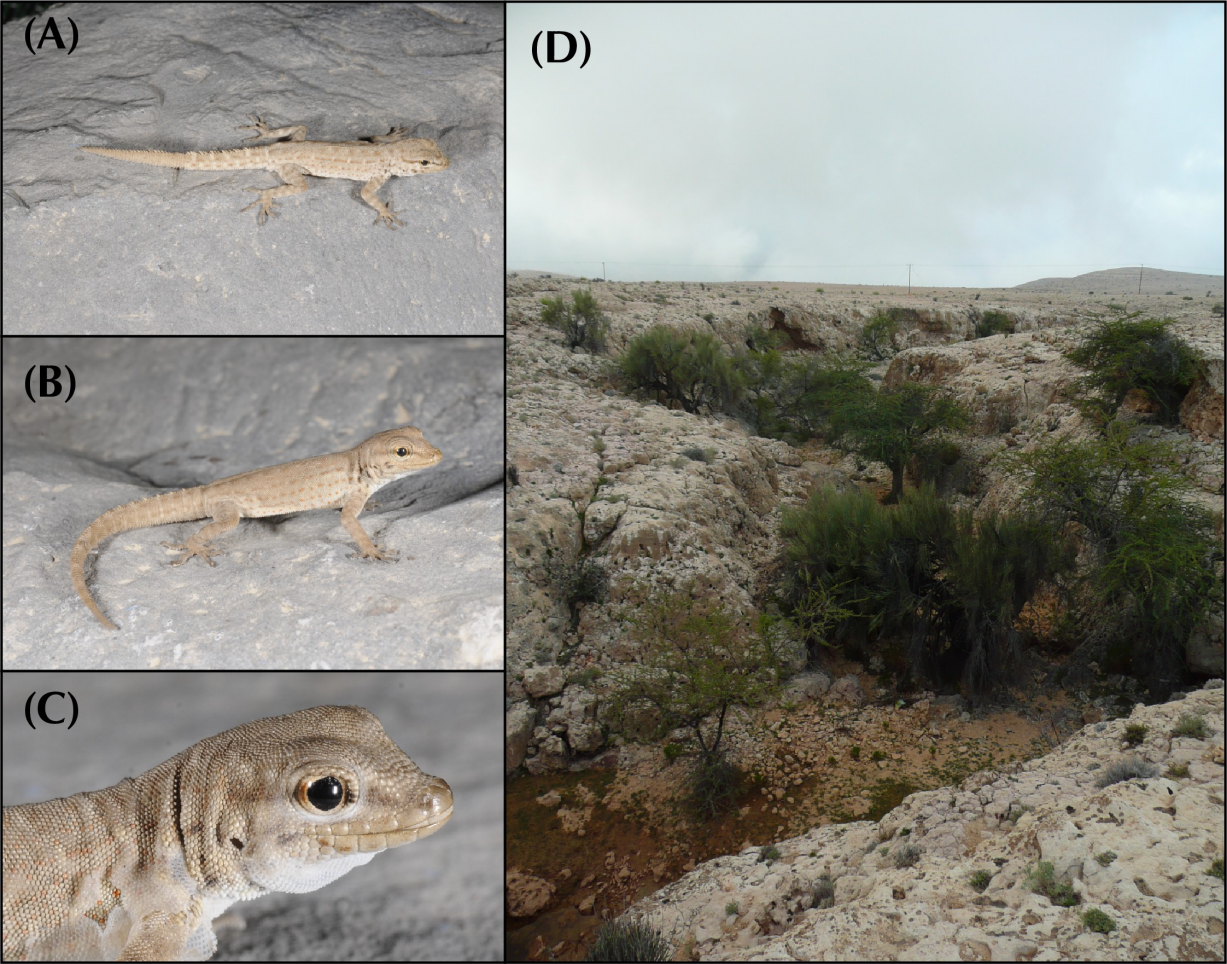
**(A)** Live male specimen of *Pristurus ali* **sp. nov.** lineage 6 *sensu* Garcia-Porta et al. (2017); **(B)** Male specimen from lineage 7 *sensu* Garcia-Porta et al. (2017); **(C)** Head close-up of *P*. *ali* **sp. nov.** specimen from lineage 6 sensu Garcia-Porta et al. (2017) (picture code: BPP6_SC42502); **(D)** Habitat of *P*. *ali* **sp. nov.** near Sayma road, type locality. All pictures taken by S.C.

**Fig 17:**
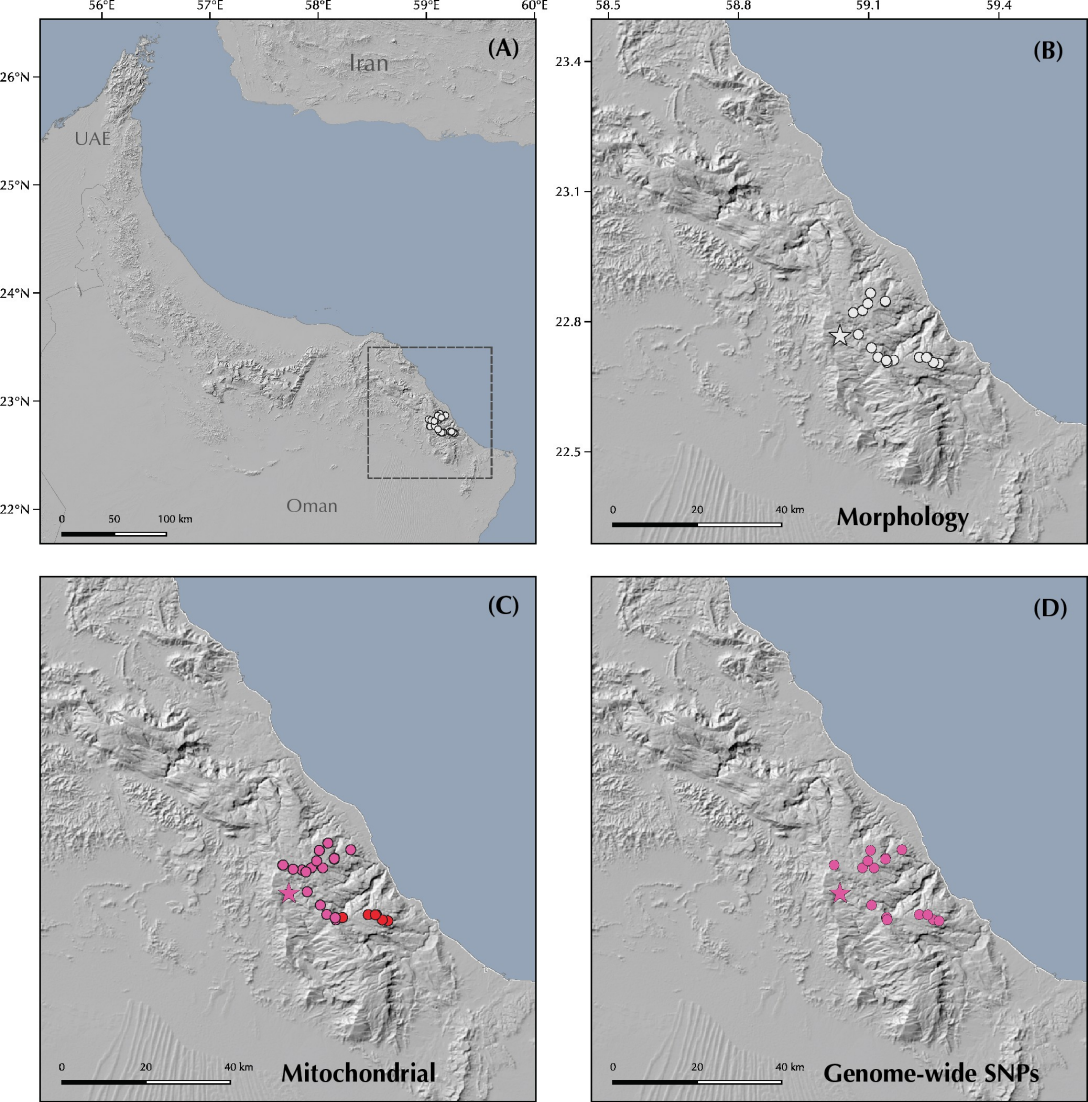
Distribution of *P*. *ali* **sp.nov.** in Arabia, including **(A)** Distribution of the species throughout the Hajar Mountains; **(B)** specimens used for morphological multivariate analyses; **(C)** specimens used for sympatry analysis identified as *P*. *r*. *rupestris* candidate species 6 and 7 by Garcia-Porta et al. (2017) using the 12S mitochondrial genetic marker; **(D)** specimens used for genome-wide SNP analyses. Type locality for the species is represented with a pink or white star. Colors refer to the following populations: Pink: *P*. *r*. *rupestris* candidate species 7 from Garcia-Porta et al. (2017) and *P*. *rupestris* BFD4 from Burriel-Carranza et al. (2024); red: Candidate species 6 from Garcia-Porta et al. (2017) and *P*. *rupestris* BFD4 from Burriel-Carranza et al. (2024).

#### Pristurus rupestris

Arnold, 1977: 106 (part.); Arnold, 1986: 421 (part.); Arnold, 2009: 3 (part.); van deer Kooij, 2000: 116 (part.); Sindaco and Jeremčenko, 2008: 122 (part.); Gardner, 2013: 178 (part.).

*Pristurus rupestris rupestris* candidate species 6 and 7. Garcia-Porta et al., 2017: 5.

*Pristurus r*. *rupestris* Clade 2. Garcia-Porta et al., 2017: 5; Saberi-Pirooz et al., 2019:11.

*Pristurus* sp2. Šmíd et al., 2021: 1189; Tejero-Cicuendez et al., 2021: S1 Fig; Tejero-Cicuéndez et al., 2022: S1 Fig; Burriel-Carranza et al., 2022: 9; Burriel-Carranza et al., 2024: S1 Fig; Carranza et al., 2018: S2 Appendix, 63; Carranza et al., 2021: 119.

*Pristurus rupestris* SP6 and SP7. Saberi-Pirooz et al., 2019: 11.

*Pristurus rupestris* BFD4. Burriel-Carranza et al., 2024: S24 Fig

#### Holotype

ZFMK 104093 (sample code S7547), adult male from Sayma road, 1,327 m asl, Eastern Hajars (North Oman), 22.76619’N, 59.03366’E, WGS84, collected in May 2011 by S. Carranza, F. Amat and E. Gómez-Díaz (assigned to *Pristurus rupestris rupestris* candidate species 7 from Garcia-Porta et al. 2017; *Pristurus rupestris* BFD4 from Burriel-Carranza et al. 2024; see Table 1) [27, 28].

#### Paratypes

IBES7509 (sample code S7509; MorphoBank M849691–M849693), adult male from Tiwi road, 1,717 m, Eastern Hajars (North Oman), 22.82554’N, 59.08568’E, WGS84, collected in May 2011 by S. Carranza, F. Amat and E. Gómez-Díaz (assigned to *Pristurus rupestris rupestris* candidate species 7 from Garcia-Porta et al. 2017; *Pristurus rupestris* BFD4 from Burriel-Carranza et al. 2024; see Table 1) [27, 28]; IBECN4240 (sample code CN4240; MorphoBank M849687–M849689), adult male from Al Salil, 1,691 m, Eastern Hajars (North Oman), 22.83206’N, 59.02031’E WGS84, collected in May 2013 by S. Carranza, F. Amat, M. Metallinou, P. de Pous and Marc Simó-Riudalbas (assigned to *Pristurus rupestris rupestris* candidate species 7 from Garcia-Porta et al. 2017; *Pristurus rupestris* BFD4 from Burriel-Carranza et al. 2024; see Table 1) [27, 28]; IBES7193 (sample code S7193; MorphoBank M849682–M849685), adult male from Tiwi road, 1,533 m, Eastern Hajars (North Oman), 22.82532’N, 59.11173’E, WGS84, collected in December 2010 by J. Garcia-Porta (assigned to *Pristurus rupestris rupestris* candidate species 7 by Garcia-Porta et al. 2017; *Pristurus rupestris* BFD4 from Burriel-Carranza et al. 2024; see Table 1) [27, 28]; NHMOK 2653 (sample code S7226), adult male from Jabal Bani Jabir, 1,956 m, Eastern Hajars (North Oman), 22.71105’N, 59.14123’E, WGS84, collected in December 2010 by J. Garcia-Porta (assigned to *Pristurus rupestris rupestris* candidate species 7 from Garcia-Porta et al. 2017; *Pristurus rupestris* BFD4 from Burriel-Carranza et al. 2024; see Table 1) [27, 28]; IBES7539 (sample code S7539; MorphoBank M849700–M849703), adult female from Jabal Bani Jabir, 2,045 m, Eastern Hajars (North Oman), 22.70583’N, 59.1421’E, WGS84, collected in May 2011 by S. Carranza, F. Amat and E. Gómez-Díaz (assigned to *Pristurus rupestris rupestris* candidate species 7 from Garcia-Porta et al. 2017; *Pristurus rupestris* BFD4 from Burriel-Carranza et al. 2024; see Table 1) [27, 28]; MZB 2024–0983 (sample code S7225; MorphoBank M849705–M849708), adult female from Tiwi road, 1,533 m, Eastern Hajars (North Oman), 22.82532’N, 59.11173’E, WGS84, collected in December 2010 by J. Garcia-Porta (assigned to *Pristurus rupestris rupestris* candidate species 7 from Garcia-Porta et al. 2017; *Pristurus rupestris* BFD4 from Burriel-Carranza et al. 2024; see Table 1) [27, 28]; MZB 2024–0984 (sample code S7563; MorphoBank M849695–M849698), adult female from Tiwi road, 1,559 m, Eastern Hajars (North Oman), 22.84148’N, 59.09768’E, WGS84, collected in May 2011 by S. Carranza, F. Amat and E. Gómez-Díaz (assigned to *Pristurus rupestris rupestris* candidate species 7 from Garcia-Porta et al. 2017; *Pristurus rupestris* BFD4 from Burriel-Carranza et al. 2024; see Table 1) [27, 28]; IBECN8286 (sample code CN8286; MorphoBank M849715–M849719), adult female from Tiwi road, 2,046 m, Eastern Hajars (North Oman), 22.71016’N, 59.14027’E, WGS84, collected in November 2014 by S. Carranza (assigned to *Pristurus rupestris rupestris* candidate species 7 from Garcia-Porta et al. 2017; *Pristurus rupestris* BFD4 from Burriel-Carranza et al. 2024; see Table 1) [27, 28]; IBES7211 (sample code S7211; MorphoBank M849710–M849713), adult female from Jabal Bani Jabir, 1,956 m, Eastern Hajars (North Oman), 22.71105’N, 59.14123’E, WGS84, collected in December 2010 by J. Garcia-Porta (assigned to *Pristurus rupestris rupestris* candidate species 7 by Garcia-Porta et al. 2017; *Pristurus rupestris* BFD4 from Burriel-Carranza et al. 2024; see Table 1) [27, 28].

#### Other material examined

Additional *Pristurus ali* **sp. nov.** specimens previously assigned to *P*. *r*. *rupestris* candidate species 7 (same as type specimens) and 6 from Garcia-Porta et al. (2017) [27], and to *P*. *rupestris* BFD4 from Burriel-Carranza et al. (2024) [28] that were used for morphological, genetic, genomic, and geographical analyses can be consulted in Table 1.

#### Etymology

The species epithet ‘*ali*’ is dedicated to Ali Alghafri from the Environment Authority, Oman in recognition for his companionship, guidance and help during several field expeditions across the Hajar Mountains of Oman dedicated to the study of the geckos of the *Pristurus rupestris* species complex, among other reptiles. The species epithet name is defined as a noun in apposition (not a noun in the genitive case) to avoid ending with a non-euphonious double-i.

#### Diagnosis

*Pristurus ali* **sp. nov.** corresponds to a genetically highly distinct lineage from the *Pristurus rupestris* species complex *sensu* Garcia-Porta et al. (2017) [27] inhabiting the Eastern Hajar Mountains (North Oman). It represents the second oldest split within the *Pristurus rupestris* species complex, having diverged from all the other species within the group (except *Pristurus omanensis* **sp. nov.** which represents the oldest split) around 6.9 mya (95% HPD: 5.6–8.2 mya; Fig 5), and is restricted to a somewhat small geographic range (Fig 17). This small species of *Pristurus* is characterized by a combination of the following characters: (1) a maximum recorded SVL of 25.8 mm; (2) 6–7 upper labial scales; (3) 4 lower labial scales; (4) 3–6 small postmental scales; (5) absence of mid-dorsal enlarged scales (crest) on the body; (6) laterally compressed tail usually with conspicuous dorsal and smaller ventral enlarged scales (crest) in males, and less conspicuous (sometimes absent) in females; (7) five unique mutations in the *mc1r* alignment: C instead of T in position 147; G instead of A in position 286; C instead of T in position 399; G instead of A in position 531; C instead of T in position 648; (8) five unique mutations in the *cmos* alignment: C instead of T in position. 6; G instead of A in position 98; A instead of C in position 147; C instead of T in position 153; A instead of G in position 225; (9) 1 unique mutation in the *rag1* alignment: A instead of G in position 206; (10) 1 unique mutation in the *rag2* alignment: G instead of T in position 405 (see S1 Appendix).

#### Morphological, genetic and phylogeographic remarks

Morphological differentiation of *Pristurus ali* **sp. nov.** from other species of the *Pristurus rupestris* species complex is convoluted, and even though there are no unique diagnostic characters to distinguish these species, *P*. *ali* **sp. nov.** specimens tentatively have a more robust body, specifically shorter brachia and wider scapular girdles, than *Pristurus rupestris* sensu stricto, *P*. *omanensis* **sp. nov.** and *P*. *feulneri* **sp. nov.** (which is described below and corresponds to *P*. *r*. *rupestris* candidate species 10.11,12–15 in Garcia-Porta et al. 2017; *Pristurus rupestris* BFD7–BFD11 in Burriel-Carranza et al. 2024) [27, 28] as evidenced by the morphological multivariate analyses (Fig 3A and S2–S4 Tables). The reconstructed morphospace of the *P*. *rupestris* species complex represented by the first two Principal Components of the PCA (explaining almost 50% of all shape variance; Fig 3A), revealed some differentiation of *Pristurus ali* **sp. nov.**, yet the range of variation of this species still overlapped with the other taxa. Procrustes ANOVA and subsequent pairwise comparisons generally corroborated these findings, where differences among candidate species were found between *P*. *ali* **sp. nov.** and all other taxa with the exception of the below described *P*. *assareen* **sp. nov.** (*P*. *r*. *rupestris* candidate species 16 in Garcia-Porta et al. 2017; *Pristurus rupestris* BFD12 in Burriel-Carranza et al. 2024) [27, 28]. Specifically, differences between these candidate species were due to distinct head dimensions, shorter brachium length and wider scapular and pelvic girdles in *P*. *ali* **sp. nov.**, as evidenced by PC loadings and procrustes ANOVAs on the individual variables (S2–S4 Tables). These findings are in agreement with Garcia-Porta et al. (2017) [27] and would correspond to the robust morphotype described therein. Comparisons of within species variance found no differences among putative taxa, which was likewise observed in the overlapping distributions in the PCA. Taken together, although our morphological analyses found some evidence that while *Pristurus ali* **sp. nov.** was somewhat distinct morphologically, there were no diagnostic morphological traits that distinguished the species unambiguously and could be used as a morphological taxonomic character for this group. *Pristurus ali* **sp. nov.** is also geographically separated from *P*. *omanensis* **sp. nov.** by its elevational occurrence across its distribution range. While both species can be found in the Eastern Hajars, the former has been only found above 900 m asl while the latter is mostly found below 700 m asl.

Molecular analyses show a strong differentiation between *P*. *ali* **sp. nov.** and all other species of the *P*. *rupestris* species complex. The results of the nuclear networks for the *cmos*, *mc1r* and *rag2* loci shown in Fig 4B, indicate that *P*. *ali* **sp. nov.** has private alleles for each of these genes (not shared with any other species included in the present analysis). *Pristurus ali* **sp. nov.** is monophyletic in both mitochondrial (Fig 4A) and nuclear (Figs 5 and S1) phylogenetic analyses. This species diverged from its sister group (all other species in the *Pristurus rupestris* species complex except for *P*. *omanensis* **sp. nov.**) about 6.8 mya, representing the second oldest divergence event within the species complex (Fig 5). Uncorrected p-distances for the mitochondrial *12S* gene (9.4–21.1%; S5 Table) as well as population genomic fixation indexes (F_ST_ > 0.65 in all pairwise comparisons; S6 Table) support a great deal of differentiation between *P*. *ali* **sp. nov.** and the other *Pristurus* species. Furthermore, no signs of gene flow are apparent between *P*. *ali* **sp. nov.** even though it has been recorded in sympatry with *P*. *rupestris* sensu stricto and the below described *P*. *feulneri* **sp. nov.** (*P*. *r*. *rupestris* candidate species 15 in Garcia-Porta et al. 2017; *P*. *rupestris* BFD11 in Burriel-Carranza et al. 2024; S7 Table) [27, 28]. Similar to *P*. *omanensis* **sp. nov.**, mitochondrial and nuclear phylogenies yielded contrasting results when interrogating the intraspecific diversity within *P*. *ali* **sp. nov.** The mitochondrial phylogenetic reconstruction recovered two distinct allopatric lineages within *P*. *ali* **sp. nov.** (corresponding to *P*. *r*. *rupestris* candidate species 6 and 7 from Garcia-Porta et al. 2017) [27]. However, when using genome-wide nuclear SNP data, this segregation disappeared in the phylogenomic reconstructions as well as in population structure analyses, and *P*. *ali* **sp. nov.** was recovered as a single, homogeneous population (corresponding to *Pristurus rupestris* BFD4 in Burriel-Carranza et al. 2024) [28]. When the mitochondrial separation is enforced, the genome-wide F_ST_ value inferred with *dataset2* is as low as 0.06 (Fig 6).

#### Description of the holotype

ZFMK 104093 (sample code S7547) (Fig 14). Adult male. Complete specimen with the tip of the tongue missing (used for DNA extraction), throat damaged and tail detached in two separate parts. Data on nine morphometric and two pholidotic variables (see Materials and Methods) are provided in S1 Table. SVL 23.8 mm, head length 30.4% of SVL, and head width 63% of head length, tail tip regenerated. Moderately built, round body, habitus comparable to *P*. *rupestris* sensu stricto; limbs and tail not especially slender. Head robust and does not rise very steeply in profile, snout slightly blunt. Nostril separated from the rostral and situated between three scales: a wide supranasal extending to the midline margin, a small upper postnasal and a large lower postnasal extending laterally along the lower edge. Supranasals separated in two scales. Scales on snout granular, polygonal and slightly pointed upwards. About nine scales in a straight line from the lower postnasal to the anterior edge of the orbit. Scales on snout, anterior to eyes, larger than those on top of head. Around 21 scales across mid-orbital region. Palpebral fold edged anteriorly with large scales and not edged with pointed (ciliate) scales. Ear opening round. Upper labials (right/left) 7/7. Lower labials (right/left) 4/4, the fourth decreasing in size from the three large anterior scales. Four enlarged scales running backwards along the proximal borders of the third lower labials to end at about the level of the angle of the mouth. Mental large, wedge-shaped, truncated posteriorly by four gular scales. Gulars small, rather pointed posteriorly, becoming slightly imbricate on throat. Dorsal scales on body small, polygonal and homogeneous, slightly pointed posteriorly and granular. Ventrals flat and distinctly imbricate. Scales on dorsal and anterior surface of the forelimbs large, flat and imbricate, fairly pointed. Scales beneath the upper forelimbs granular and smaller than ventral body scales; those beneath lower forelimbs large, flat, imbricate and polygonal, larger than ventral body scales. Claws relatively shallow and weakly recurved. Scales on dorsal and anterior surface of hindlimbs large, flat and imbricate, fairly pointed. Scales beneath the upper hindlimbs granular and smaller than ventral body scales, those beneath lower hindlimbs large, flat, imbricate and polygonal, larger than ventral body scales. Tail relatively short and robust, anteriorly more or less cylindrical, posteriorly slightly compressed, with a slight groove along each side. Ridges of strongly elongated scales present, low crest on midline and on lower mid-line of tail is made up of a longitudinal row of enlarged scales, more enlarged dorsally. Tail scales large, flat and imbricate, pointed posteriorly; dorsals and ventrals considerably larger than those on body.

Coloration in alcohol is light brown above; broad but blurred dorsal midline pale and slightly grayish. Dorsolaterally discontinuous thin pale reddish lines that dissolve into rows of small spots ventrolaterally to the flanks. Pale whitish spots distributed in an irregular pattern dorsolaterally, and roughly double the diameter of the reddish spots. Head light brown with a broad dark brownish stripe running from close to the nostril through the eye and then dorsolaterally until above the ear opening. Single large dark spots located right beneath the angle of the mouth until the third lower labial scale. Same-colored but roughly half the diameter sized spots conformed by two to three gular scales present on the ventral side of the throat, distributed from the level of the anterior eye opening to the insertion of the forelimbs. Ventral coloration whitish. Limbs light brown with irregular dark brown markings above, ventrally pale, toes not banded. Tail similarly colored like dorsals, pale gray-brown above with blurred, light, narrow cross-bars in the non-regenerated part.

The figured μ-CT-scanned specimen (Fig 14B) has a maximal skull length of 7.8 mm, maximal height of 3.4 mm and maximal width of 4.5 mm. 26 presacral vertebrae. Noteworthy minor qualitative cranial osteological differences (Fig 14C) to the other holotype specimens of the *Pristurus rupestris* species complex are: Quadratum well-developed and slightly laterally protruding; ossified cranium; sclerotic rings strongly ossified; vomer posteriorly rounded and bordered by a heart-shaped opening; osseous naris edge smooth and slightly rounded; premaxilla and nasal rising moderately steep to frontal region.

#### Variation

Data on nine morphometric and two pholidotic variables (see Materials and methods) for all nine paratypes (see above) are provided in S1 Table. Largest male 25.7 mm SVL; largest female 24.3 mm. Eight of nine paratypes have completely or partially broken tails; three of nine have the throat sliced. Paratype IBECN4240 (sample CN4240) has a partially regenerated tail. Dorsal coloration ranging from pale beige to grayish, with only blurred and thin pale to orangish dorsal midlines present in some individuals usually in females. Multiple small dark brownish gular spots present in all paratypes, ranging from a blend to prominent appearance. Ventrally inconspicuous dark brownish to reddish small spots present in some paratypes, but ventrolaterally only and not reaching to midventral region (Fig 15).

#### Distribution

*Pristurus ali* **sp. nov.** is endemic to Oman, where it has only been found in the Eastern Hajars (Fig 17). Despite intensive surveys across the Hajar Mountain range, it has never been found to the West of the Eastern Hajars. It is only found in mid to high elevations as it has only been recorded from 919 m asl up to 2,046 m asl.

#### Habits

*Pristurus ali* **sp. nov.** is a diurnal species, although it can also be seen active after sunset. It has been found on rocks, boulders and tree trunks but most probably it is also present inside the mountain villages on human-made structures such as walls, houses, and gardens (Fig 16). It performs complex signaling including push-ups, inflating and laterally compressing the body and throat, and curling and waving the tail up over the back. It preys on ants and other arthropods using a sit-and-wait strategy. Females lay single hard-shelled eggs throughout the year.

#### Conservation

This species is endemic to a restricted area of the Eastern Hajar Mountains in Oman. It has an area of occupancy (AOO) of 76 km^2^ and an extent of occurrence (EOO) of 307 km^2^. Despite the restricted EOO and AOO, there are no known threats and no continuing decline in habitat area or quality, and the species should be assessed as Least Concern in view of its distribution, abundance, and presumed large and stable population.

### *Pristurus feulneri* Burriel-Carranza, Koppetsch, Garcia-Porta, Els, Carranza–sp. nov. urn:lsid:zoobank.org:act:11000749-4B61-446D-B734-042F6EF7C9E4

(Figs 2–6, 18–21, S5–S8 Tables 1 and S1, S2, S1–S7; Alignment of phased haplotypes for the molecular diagnostic characters based on the *mc1r*, *cmos*, *rag1* and *rag2* genes can be found on S1 Appendix).

English name: Feulner’s Semaphore Gecko

**Fig 18:**
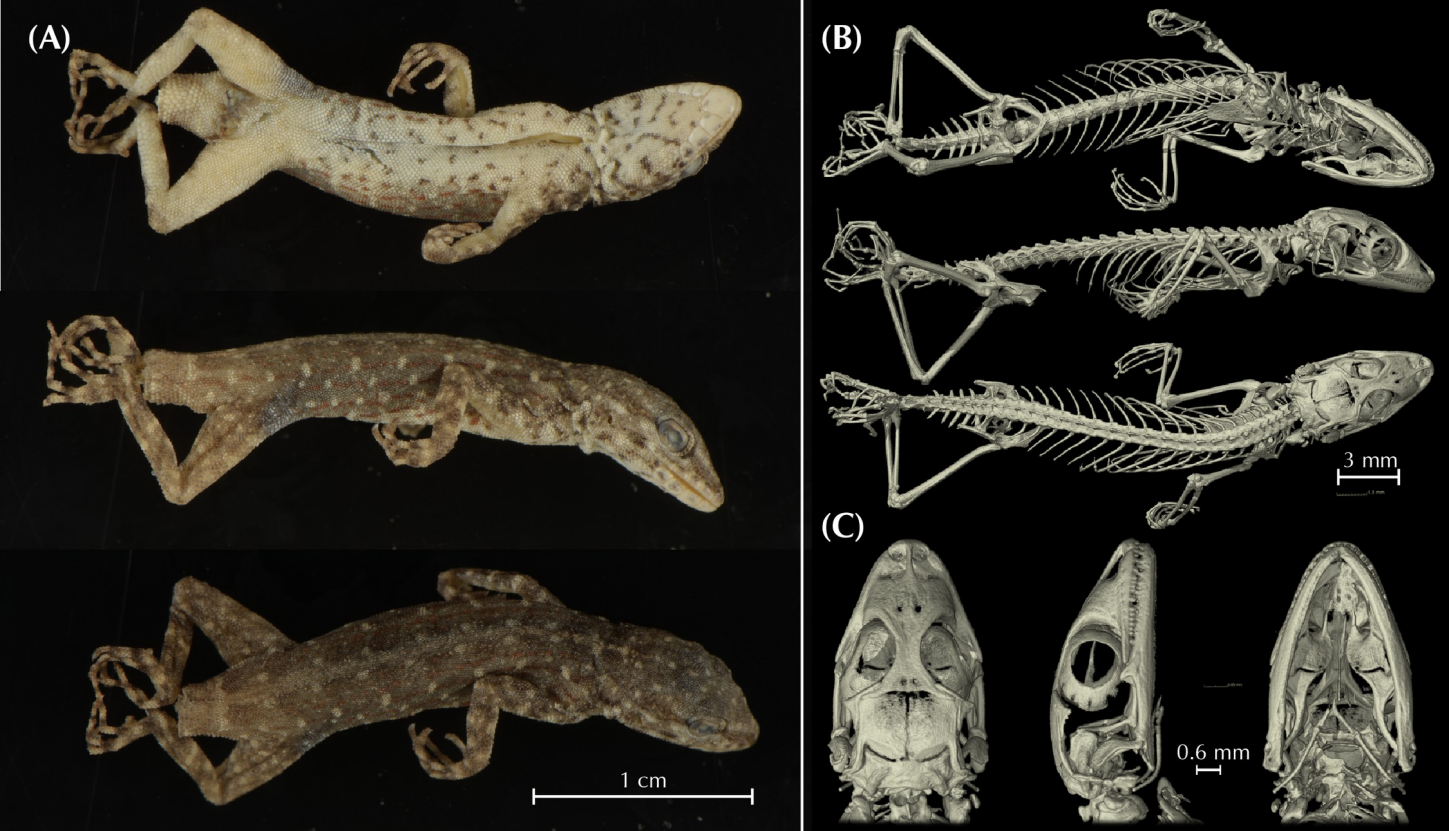
External and internal morphology of *Pristurus feulneri* sp. nov. holotype specimen ZFMK104094 (sample code S6102). **(A)** Ventral, lateral and dorsal external view of the specimen; **(B)** Ventral, lateral and dorsal internal view obtained through μ-CT scanning; **(C)** close up of head bone structures of the holotype specimen ZFMK104094 (sample code S6102).

**Fig 19:**
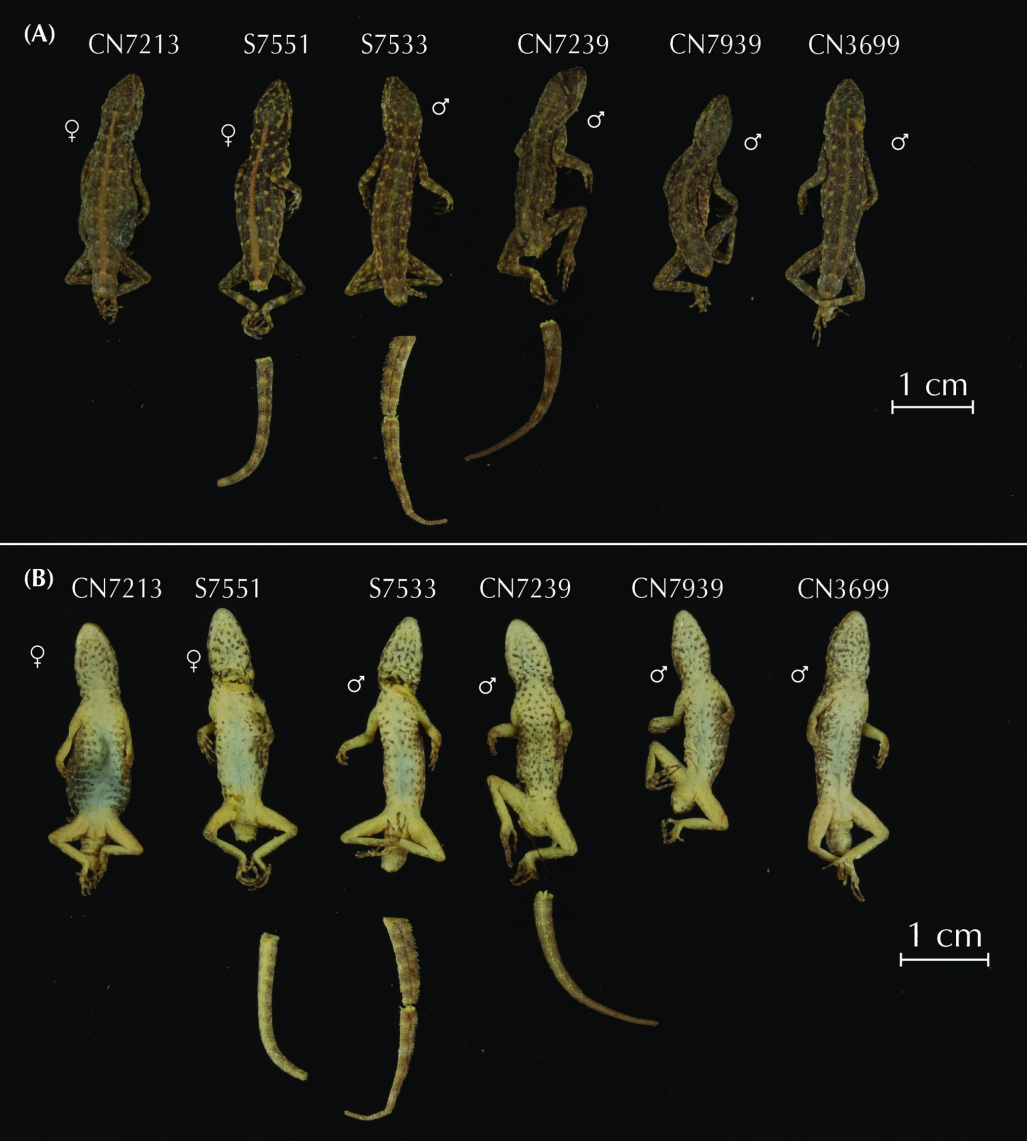
Dorsal **(A)** and ventral **(B)** view of *Pristurus feulneri* **sp. nov.** paratype specimens. To be taxonomically coherent, all paratype specimens were chosen from the same genetic lineage (genetic lineage 13 *sensu* Garcia-Porta et al. 2017; genomic lineage BFD9 *sensu* Burriel-Carranza et al. 2024). Further variation in *P*. *feulneri* **sp. nov.** specimens lineages 10.11, 12, 14, and 15 *sensu* Garcia-Porta et al. (2017) (lineages BFD7,8,10, and BFD11 *sensu* Burriel-Carranza et al. 2024) can be consulted in S5–S8 Figs.

**Fig 20:**
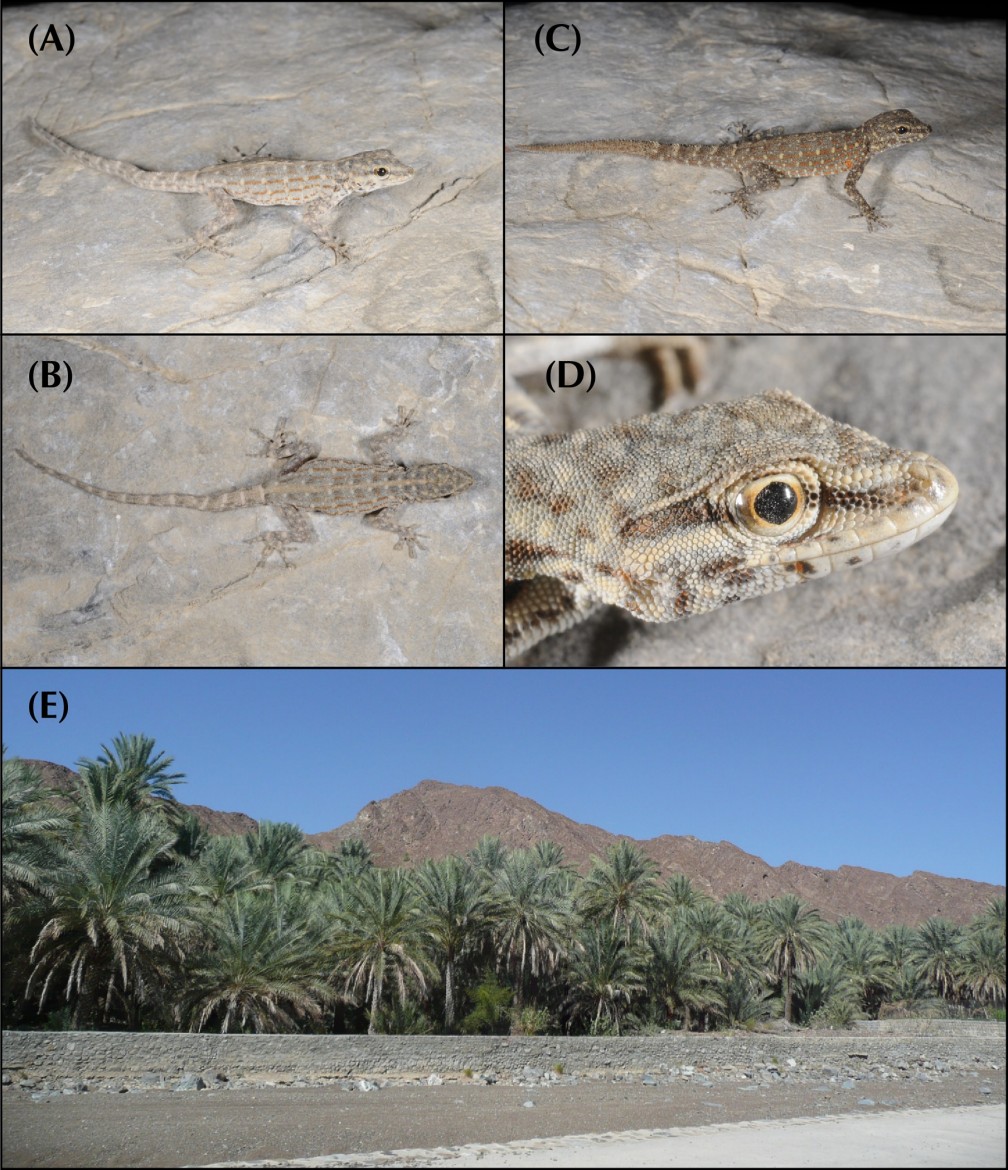
**(A)** *P*. *feulneri* **sp. nov.** female specimen from lineage 12 *sensu* Garcia-Porta et al. (2017) with pale coloration, and a conspicuous dorsal line; **(B)** Dorsal view of the same specimen; **(C)** *P*. *feulneri* **sp. nov.** male specimen with dark coloration and a conspicuous dorsal crest in tail. The specimen corresponds to *P*. *feulneri* **sp. nov.** lineage 12 *sensu* Garcia-Porta et al. (2017); **(D)** Head close-up of a *P*. *feulneri* **sp. nov.** lineage 15 *sensu* Garcia-Porta et al. (2017) specimen from the Eastern Hajars; (**E**) Habitat of *P*. *feulneri* **sp. nov.** near Mizbar, locality of some paratype specimens. All pictures taken by S.C.

**Fig 21:**
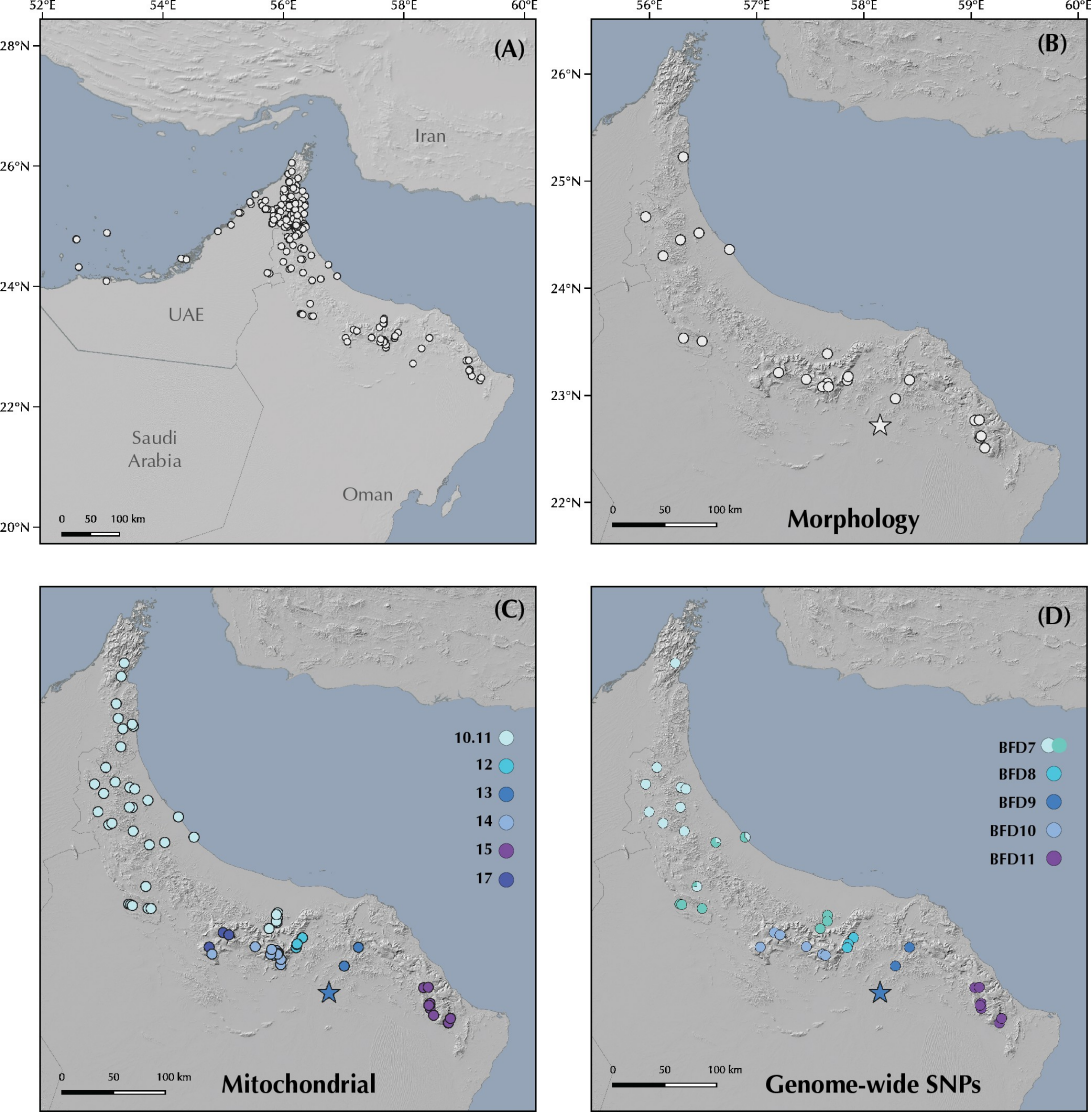
Distribution of *P*. *feulneri* **sp.nov.** in Arabia, including: **(A)** all known introduced populations throughout the coast of UAE and surrounding islands; **(B)** specimens used for morphological multivariate analyses; **(C)** specimens used for sympatry analysis identified as *P*. *rupestris* candidate species 10.11–15 by Garcia-Porta et al. (2017) using the 12S mitochondrial genetic marker, including specimens from lineage 17 discovered in the present study; **(D)** specimens used for genome-wide SNP analyses colored by the most supported ADMIXTURE configuration in Burriel-Carranza et al. (2024). Type locality for the species is represented with a blue or white star. Legend in caption **(C)** refer to *P*. *feulneri* **sp. nov.** candidate species within Garcia-Porta et al. (2017). Legend in caption **(D)** correspond to *P*. *feulneri* **sp. nov.** lineages within Burriel-Carranza et al. (2024).

#### Pristurus rupestris

Arnold, 1977: 106 (part.); Arnold, 1986: 421 (part.); Arnold, 2009: 3 (part.); van deer Kooij, 2000: 116 (part.); Sindaco and Jeremčenko, 2008: 122 (part.); Gardner, 2013: 178 (part.).

*Pristurus rupestris rupestris* candidate species 10.11, 12, 13, 14 and 15. Garcia-Porta et al., 2017: 5.

*Pristurus r*. *rupestris* Clade 3. Garcia-Porta et al., 2017: 5; Saberi-Pirooz et al., 2019:11.

*Pristurus* sp3. Šmíd et al., 2021: 1189; Tejero-Cicuendez et al., 2021: S1 Fig; Tejero-Cicuéndez et al., 2022: S1 Fig; Burriel-Carranza et al., 2019: S1 Appendix: 35; Burriel-Carranza et al., 2022: 9; Burriel-Carranza et al., 2024: S1 Fig; Carranza et al., 2018: S2 Appendix, 64; Carranza et al., 2021: 119).

*Pristurus rupestris* SP10/11, SP12, SP13, SP14 and SP15. Saberi-Pirooz et al., 2019: 11.

*Pristurus rupestris* BFD7, BFD8, BFD9, BFD10 and BFD11. Burriel-Carranza et al., 2024: S24 Fig.

#### Holotype

ZFMK 104094 (sample code S6102), adult male from Lizq, 482 m, Eastern Hajars (North Oman), 22.71765’N, 58.15035’E, WGS84, collected in November 2010 by J. Garcia-Porta (assigned genetically to *Pristurus rupestris rupestris* candidate species 13 from Garcia-Porta et al. 2017 and to *Pristurus rupestris* BFD9 from Burriel-Carranza et al. 2024; see Table 1) [27, 28].

#### Paratypes

IBECN3699 (sample code CN3699; adult male; MorphoBank M849798–M849800), MZB 2024–0986 (sample code CN7939; adult male; MorphoBank M849785–M849787), IBECN7239 (sample code CN7239; adult male; MorphoBank M849778–M849783), MZB 2024–0985 (sample code CN7213; adult female; MorphoBank M849789–M849791) four specimens from Mizbar, 549 m, Eastern Hajars (North Oman), 23.14319’N, 58.42435’E, WGS84, collected in November 2014 by S. Carranza (assigned to *Pristurus rupestris rupestris* candidate species 13 from Garcia-Porta et al. 2017; *Pristurus rupestris* BFD9 in Burriel-Carranza et al. 2024; see Table 1) [27, 28]; IBES7533 (sample code S7533; MorphoBank M849793–M849796), adult male; NHMOK 2655 (sample code S7551), adult female; two specimens from Mizbar, 555 m, Eastern Hajars (North Oman), 23.14307’N, 58.4244’E, WGS84, collected in May 2011 by S. Carranza, F. Amat and E. Gómez-Díaz (assigned to *Pristurus rupestris rupestris* candidate species 13 from Garcia-Porta et al. 2017; *Pristurus rupestris* BFD9 in Burriel-Carranza et al. 2024; see Table 1) [27, 28].

#### Other material examined

Additional *Pristurus feulneri* **sp. nov.** specimens formerly assigned to *P*. *r*. *rupestris* candidate species 10.11, 12, 13, 14 and 15 from Garcia-Porta et al. (2017) [27] and to *Pristurus rupestris* BFD7–BFD11 in Burriel-Carranza et al. (2024) [28] that were used for morphological, genetic, genomic, and geographical analyses can be consulted in Table 1.

#### Etymology

The species epithet “*feulneri*” is a genitive Latin noun in honor of American Naturalist Gary R. Feulner, Chairman of the Dubai Natural History Group since 1995, in recognition for his 39 years of dedication and contributions to advancing our understanding of the biodiversity and biogeography of the fauna and flora of the Hajar Mountains.

#### Diagnosis

*Pristurus feulneri* **sp. nov.** corresponds to a genetically highly distinct lineage from the diverse *Pristurus rupestris* species complex inhabiting the Hajar Mountains (North Oman and Eastern UAE). This species represents the most abundant *Pristurus* species of the Hajar Mountains and is distributed across the whole mountain range (Fig 21). *Pristurus feulneri* **sp. nov.** is characterized by a combination of the following characters: (1) a maximum recorded SVL of 27.3 mm; (2) 6–7 upper labial scales; (3) 4–5 lower labial scales; (4) 3–6 small postmental scales; (5) absence of mid-dorsal enlarged scales (crest) on the body; (6) laterally compressed tail usually with conspicuous dorsal and smaller ventral enlarged scales (crest) in males, and less conspicuous (sometimes absent) in females; (7) one unique mutation in alignment *cmos*: an A instead of a G in position 241; (8) two unique mutations in alignment *rag1*: T instead of C in position 94; A instead of G in position 206 (see S1 Appendix).

#### Morphological, genetic and phylogeographic remarks

*Pristurus feulneri* **sp. nov.** is morphologically cryptic from the other species of the *Pristurus rupestris* species complex.

**Fig 22:**
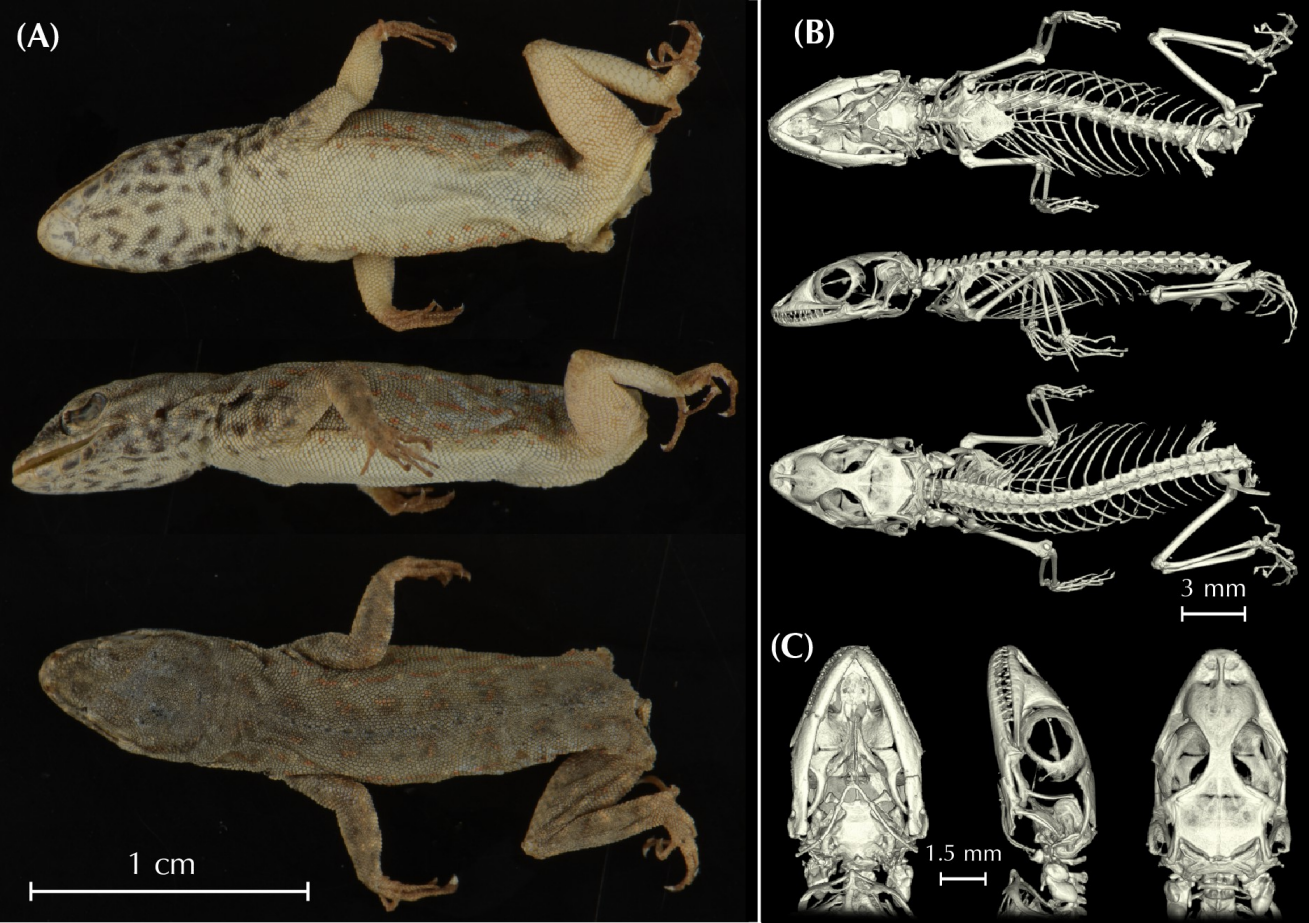
External and internal morphology of *Pristurus assareen* sp. nov. holotype specimen ZFMK104095 (sample code CN3704). **(A)** Ventral, lateral and dorsal external view of the specimen; **(B)** Ventral, lateral and dorsal internal view obtained through μ-CT scanning; **(C)** close up of head bone structures of the *P*. *assareen* **sp. nov.** holotype specimen.

The reconstructed morphospace of the *P*. *rupestris* species complex (Fig 3A) revealed that *P*. *feulneri*’s **sp. nov.** morphospace completely overlaps with *P*. *rupestris* sensu stricto and *P*. *omanensis* **sp. nov.**, and presents a great deal of overlap with *P*. *ali* **sp. nov.** and *P*. *assareen* **sp. nov.** (*Pristurus rupestris* candidate species 16 in Garcia-Porta et al. 2017; *Pristurus rupestris* BFD12 in Burriel-Carranza et al. 2024; described below) [27, 28]. Procrustes ANOVA and subsequent pairwise comparisons (S3 and S4 Tables) corroborated that there were no significant differences between *P*. *feulneri* **sp. nov.** and the first two species while there were significant differences with the last two. Specifically, differences between these candidate species were due to distinct head dimensions, longer brachium length, and narrower scapular and pelvic girdles in *P*. *feulneri* **sp. nov.**. These findings are in agreement with Garcia-Porta et al. (2017) [27] and could correspond to the slender morphotype described therein. Tentatively, *P*. *feulneri* **sp. nov.** seems to present a more apparent dorsal midline in both males and females (especially in specimens from the Western and Central Hajars; Figs 19 and S5–S8), than *P*. *rupestris* sensu stricto (Fig 7), *P*. *omanensis* **sp. nov.** (Figs 11, 12 and S3), *P*. *ali* **sp. nov.** (Figs 14, 15 and S4), and *P*. assareen **sp. nov.** (*Pristurus rupestris rupestris* candidate species 16. Garcia-Porta et al. 2017; *Pristurus rupestris* BFD12. Burriel-Carranza et al., 2024; Figs 21 and 22; described below) [27, 28]. *Pristurus feulneri* **sp. nov.** occurs syntopically with *P*. *rupestris* sensu stricto, *P*. *omanensis* **sp. nov.** and *P*. *ali* **sp. nov.** (S7 Table).

Molecular analyses on mitochondrial (Fig 4A), nuclear loci (Fig 4B), and genome-wide SNP data (Figs 3B, 3C, 5, 6, S1 and S2) are fully concordant with the differentiation of *P*. *feulneri* as a distinct species from the other representatives of the *Pristurus rupestris* species complex. *Pristurus feulneri* is monophyletic in both mitochondrial (Fig 3A) and nuclear phylogenetic analyses (Figs 5 and S1). In all phylogenetic reconstructions, it forms a clade together with *P*. *rupestris* sensu stricto and the fourth species of *Pristurus* described below (*Pristurus rupestris rupestris* candidate species 16. Garcia-Porta et al. 2017; *Pristurus rupestris* BFD12. Burriel-Carranza et al., 2024) [27, 28], which split apart about 4.5 mya (95% HPD: 3.6–5.3 mya; Fig 5). The results of the nuclear networks for the *mc1r*, *cmos*, *rag1 and rag2* loci shown in Fig 4B, indicate that *P*. *feulneri* **sp. nov.** has some private alleles for each of these genes (not shared with any other species included in the present analysis), especially in *cmos* where all haplotypes are private. Uncorrected p-distances with the mitochondrial *12S* marker (13.5–24.5%; S5 Table) as well as F_ST_ estimates (0.61–0.83; S6 Table) with genome-wide SNP data show a strong interspecific genetic differentiation.

*Pristurus feulneri* **sp. nov.** is the species of the *Pristurus rupestris* species complex with the highest intraspecific diversity (uncorrected *p*-distance of the mitochondrial *12S* gene = 8.4%; S5 Table). Phylogenetic reconstructions with the mitochondrial marker *12S* recovered similar results to the ones observed in Garcia-Porta et al. (2017) [27]. However, with the inclusion of new specimens not present in the former study, we were able to identify another deep lineage within *Pristurus feulneri* **sp. nov.** from the Central Hajars, to which we assigned the lineage number 17 (Fig 4A). Intraspecific genome-wide F_ST_ values inferred with *dataset2* ranged from 0.38–0.69 between populations, showing a great deal of differentiation within this species (Fig 6). Moreover, species delimitation analysis inferred through BPP A10 support the existence of up to four species within *P*. *feulneri* **sp. nov.** (Fig 5) but the *gdi* approach suggest that this diversity is not fully supported as interspecific diversity with the exception of candidate species 15 in Garcia-Porta et al. (2017) (*P*. *rupestris* BFD 11 in Burriel-Carranza et al., 2024; Figs 5 and S2) [27, 28]. However, topological discordances in the internal nodes of *P*. *feulneri* **sp. nov.** were found between mitochondrial phylogenetic reconstructions (Fig 3A), phylogenomic reconstructions (S1 Fig), and MSC species tree reconstructions (Fig 5), which could suggest incomplete lineage sorting. Also, although levels of genetic divergence between candidate species 15 in Garcia-Porta et al. (2017) (*P*. *rupestris* BFD 12 in Burriel-Carranza et al., 2024) [27, 28] and the other populations within *P*. *feulneri* **sp. nov.** are high, the former seems to fall within the cluster of intraspecific divergence rather than interspecific estimates (Fig 6). Therefore, we refrain from further splitting any allopatric populations of *P*. *feulneri* **sp. nov.** until more data are available. In the Eastern Hajars we find the deepest lineage within the species, which diverged from all other *P*. *feulneri* **sp. nov.** about 2.9 mya (95% HPD: 2.3–3.4 mya; formerly assigned to *P*. *rupestris* candidate species 15 in Garcia-Porta et al. 2017; *P*. *rupestris* BFD11 in Burriel-Carranza et al. 2024; Fig 5) [27, 28]. This clade is sister to all other deep lineages of *P*. *feulneri* **sp. nov.** in the genome-wide SNP analyses (Figs 5 and S1) but conforms a monophyletic clade with lineage 17 in the mitochondrial phylogenetic reconstruction (Fig 4A). Also in the Eastern Hajars but further west, we find the population assigned to *P*. *rupestris rupestris* candidate species 13 in Garcia-Porta et al. (2017) [27] and to *P*. *rupestris* BFD9 in Burriel-Carranza et al. (2024) [28]. The greatest diversity of *P*. *feulneri* **sp. nov.** populations is found in the Central Hajars with one population inhabiting the Semail gap (*P*. *rupestris rupestris* candidate species 12 in Garcia-Porta et al. 2017; *P*. *rupestris* BFD8 in Burriel-Carranza et al. 2024) [27, 28], another population inhabiting the southern slope of the Jabal Akhdar (conformed by lineage 17 and *P*. *rupestris rupestris* candidate species 14 in Garcia-Porta et al 2017; *Pristurus rupestris* BFD10 in Burriel-Carranza et al. 2024) [27, 28], and a third population in the northern slope of the Jabal Akhdar (*P*. *rupestris rupestris* candidate species 10.11 in Garcia-Porta et al. 2017; *P*. *rupestris* BFD7 in Burriel-Carranza et al. 2024; Fig 21C and 21D) [27, 28], which extends to the West until the Musandam Peninsula and to all the coastal and island localities across the western UAE (see Distribution below). Therefore, *Pristurus feulneri* **sp. nov**. is the only species of the *Pristurus rupestris* species complex present in the UAE.

#### Description of the holotype

ZFMK 104094 (sample code S6102) (Fig 18). Adult male. Specimen ventrally sliced from gular fold to insertion of hindlimbs (internal tissue used for DNA extraction), right lower forelimb with ventral incision and tail missing (only the first 2 mm of the anterior part present). Data on nine morphometric and two pholidotic variables (see Materials and Methods) are provided in S1 Table. SVL 24.6 mm, head length 27.5% of SVL, and head width 63% of head length. Moderately built, round body, habitus comparable to *P*. *rupestris*; limbs and tail not especially slender. Head robust and does not rise very steeply in profile, snout slightly blunt. Nostril separated from the rostral and situated between three scales: a wide supranasal extending to the midline margin, a small upper postnasal and a large lower postnasal extending laterally along the lower edge. Supranasals are separated in two scales. Scales on snout granular, polygonal and slightly pointed upwards. About nine scales in a straight line from the lower postnasal to the anterior edge of the orbit. Scales on snout, anterior to eyes, are larger than those on top of head. Around 26 scales across mid-orbital region. Palpebral fold edged anteriorly with large scales and not edged with pointed (ciliate) scales. Ear opening droplet-shaped. Upper labials (right/left) 7/7. Lower labials (right/left) 4/4, the fourth decreasing in size from the three large anterior scales. Five enlarged scales running backwards along the proximal borders of the third lower labials to end at about the level of the angle of the mouth. Mental large, wedge-shaped, truncated posteriorly by four small postmental scales. Gulars small, rather pointed posteriorly, becoming slightly imbricate on throat. Dorsal scales on body small, polygonal and homogeneous, slightly pointed posteriorly. Ventrals flat and distinctly imbricate. Scales on dorsal and anterior surface of the forelimbs slightly larger than dorsal body scales, flat and imbricate, fairly pointed. Scales beneath the upper forelimbs granular and smaller than ventral body scales; those beneath lower forelimbs large, flat, imbricate and polygonal, larger than ventral body scales. Claws relatively shallow and weakly recurved. Scales on dorsal and anterior surface of hindlimbs large, flat and imbricate, fairly pointed. Scales beneath the upper hindlimbs granular and smaller than ventral body scales, those beneath lower hindlimbs large, flat, imbricate and polygonal, larger than ventral body scales. Tail rudiment cylindrical, bearing the first anterior scales of the caudal crest on midline made up of a longitudinal row of enlarged scales.

Coloration in alcohol grayish brown dorsally; broad dorsal midline pale grayish. Dorsolaterally thin pale reddish lines that dissolve into discontinuous lines posteriorly, and also ventrolaterally to the flanks. Bright whitish spots distributed in transversal rows dorsolaterally, and more than double the diameter of the reddish lines. Head same color as the body with irregularly distributed bright whitish spots. A broad dark brownish stripe running from the nostril through the eye and then dorsolaterally until above the ear opening, bordered both dorsally and ventrally by blurred pale grayish bands. Single large dark blotches located from beneath the angle of the mouth to the entire gular and ventral region reaching posteriorly to the insertion of the hindlimbs. Most of them being conformed by more than four gular scales, with larger and more prominent blotches on the throat decreasing in size posteriorly. Ventral background coloration is creamish-whitish. Limbs dark pale ventrally and brownish with bright whitish spots dorsally, forming regular bright whitish bands dorsally on the toes. Tail rudiment similarly colored like dorsals.

The figured μ-CT-scanned specimen (Fig 18B) has a maximal skull length of 7.1 mm, maximal height of 2.8 mm and maximal width of 4.1 mm. 25 presacral vertebrae. Noteworthy minor qualitative cranial osteological differences (Fig 18C) to the other holotype specimens of the *Pristurus rupestris* species complex are: Quadratum not laterally protruding; parabasisphenoid damaged; vomer posteriorly only slightly rounded and bordered by a hoof-shaped opening; osseous naris edge smooth and slightly rounded; parietal incompletely ossified; premaxilla and nasal moderately steep to frontal region.

#### Variation

Data on nine morphometric and two pholidotic variables (see Materials and Methods) for all six paratypes (see above) are provided in S1 Table. Largest male 25.3 mm SVL; largest female 24.4 mm. All paratypes have broken or incomplete tails; two of six have the throat sliced. Paratype IBECN7239 (sample CN7239) has a regenerated tail. Dorsal coloration similar for all paratypes, with conspicuous broad dorsal midline colored pale grayish to bright orange-beige. Dorsal bright spots present in all paratypes, colored pale-beige to yellowish. Multiple large gular blotches present in all paratypes. Ventral pattern variable, but dark brownish to reddish spots present in all paratypes, being distributed from ventrolaterally only to covering the entire ventral region (Fig 19).

#### Distribution

This species of *Pristurus* is the most ubiquitous of its genus in the Hajar Mountains and the only species of the *Pristurus rupestris* species complex present in the UAE. It has been recorded throughout the whole mountain range, from the Musandam Peninsula in the Western Hajars to Ras al Hadd in the Eastern Hajars. It is distributed throughout the UAE and Oman and it has been recorded from sea level up to 2,317 m asl in the Jabal Kawr (Central Hajars, Oman). There are records of introduced populations along the western UAE coast and surrounding islands (Fig 21A), most likely due to the transport of rocks and sediments from the Hajar Mountains for the development of coastline infrastructures.

#### Habits

*Pristurus feulneri* **sp. nov.** is a diurnal species, although it can also be seen active after sunset. It is abundant on rocks, boulders, tree trunks, and human-made structures such as walls, houses, and gardens (Fig 20). It performs complex signaling including push-ups, inflating and laterally compressing the body and throat, and curling and waving the tail up over the back. It preys on ants and other arthropods using a sit-and-wait strategy. Females lay single hard-shelled eggs throughout the year.

#### Conservation

*Pristurus feulneri* **sp. nov.** should be considered as Least Concern in view of its distribution, abundance, presumed large population, and because it is unlikely to be declining fast enough to qualify for listing in a threatened category.

### *Pristurus assareen* Burriel-Carranza, Koppetsch, Garcia-Porta, Carranza–sp. nov. urn:lsid:zoobank.org:act:1D989386-9EDA-44C9-9256-B9E01EECD12E

(Figs 2–6, 21–24, S1 and S2 and Tables 1 and S1–S7; Alignment of phased haplotypes for the molecular diagnostic characters based on the *mc1r*, *cmos*, *rag1* and *rag2* genes can be found on S1 Appendix).

English name: As Sareen Semaphore Gecko

**Fig 23:**
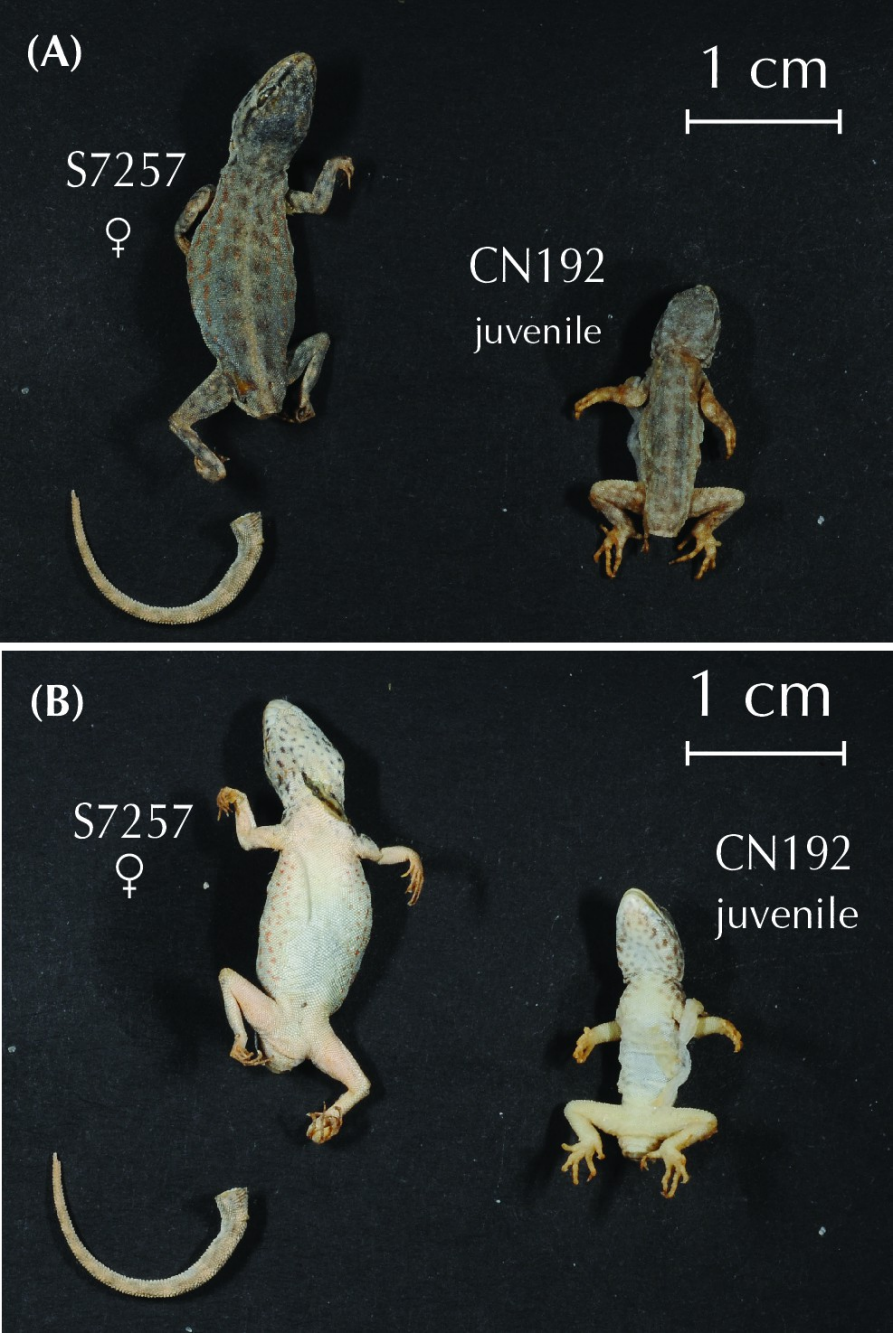
**(A)** Dorsal and **(B)** ventral view of *Pristurus assareen* **sp. nov.** paratype specimens.

**Fig 24:**
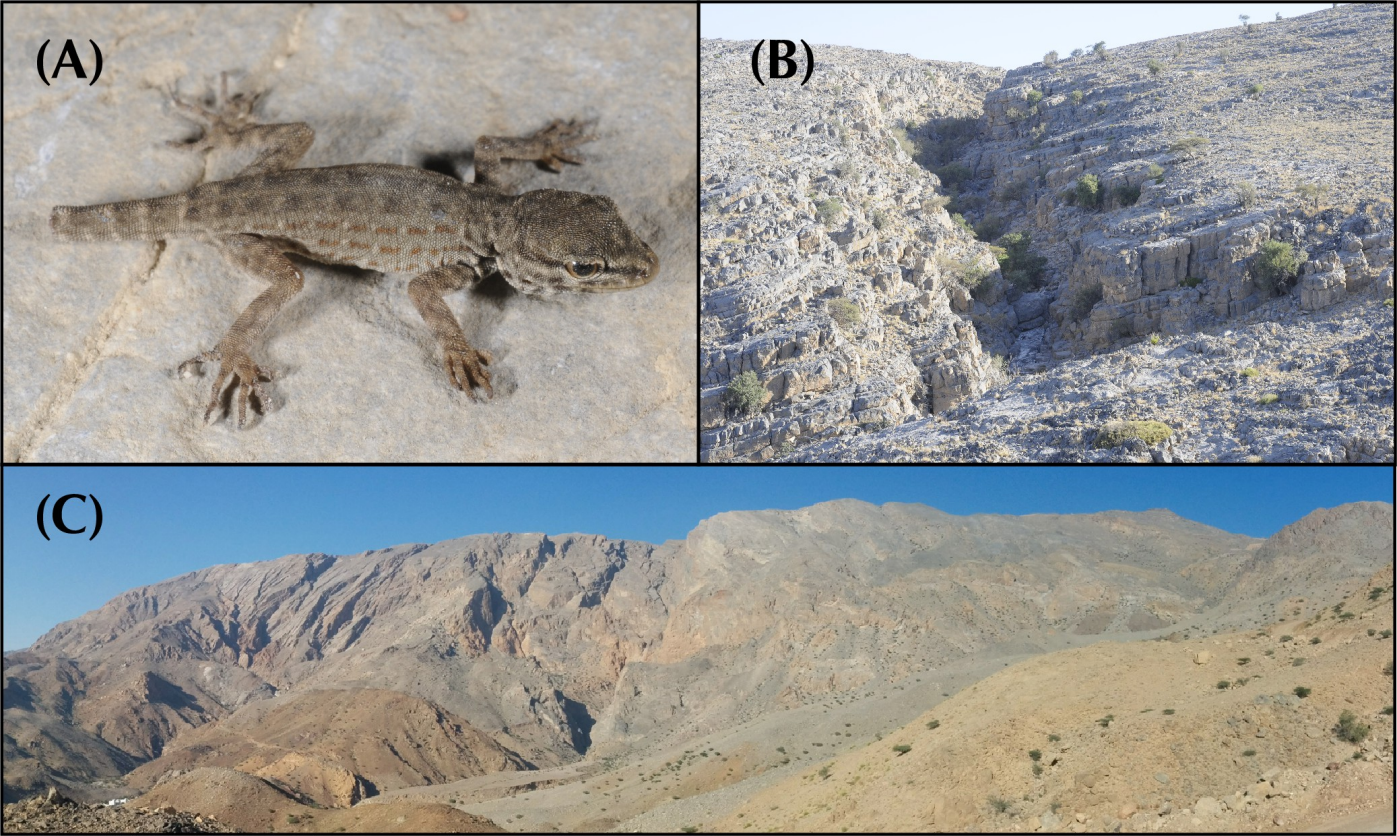
**(A)** *P*. *assareen* **sp. nov.** holotype male specimen (ZFMK104095; sample code CN3704); **(B)** Habitat of *P*. *assareen* **sp. nov.** in wadi As Sareen; **(C)** Landscape of wadi As Sareen in Eastern Hajars, North Oman. All pictures taken by S.C.

#### Pristurus rupestris

Arnold, 1977: 106 (part.); Arnold, 1986: 421 (part.); Arnold, 2009: 3 (part.); van deer Kooij, 2000: 116 (part.); Sindaco and Jeremčenko, 2008: 122 (part.); Gardner, 2013: 178 (part.).

*Pristurus rupestris rupestris* candidate species 16. Garcia-Porta et al., 2017: 5.

*Pristurus* sp4. Šmíd et al., 2021: 1189; Tejero-Cicuendez et al., 2021: S1 Fig; Tejero-Cicuéndez et al., 2022: S1 Fig; Burriel-Carranza et al., 2022: 9; Burriel-Carranza et al., 2024: S1 Fig; Carranza et al., 2018: S2 Appendix, 65; Carranza et al., 2021: 119.

*Pristurus rupestris* SP16. Saberi-Pirooz et al., 2019: 11.

*Pristurus rupestris* BFD12. Burriel-Carranza et al., 2024: S24 Fig.

#### Holotype

ZFMK 104095 (sample code CN3704), adult male from Wadi As Sareen Nature Reserve, 1,682 m asl, Eastern Hajars (North Oman), 22.13182’N, 58.61912’E, WGS84, collected in November 2014 by S. Carranza (assigned to *Pristurus rupestris rupestris* candidate species 16 from Garcia-Porta et al. 2017 and to *Pristurus rupestris* BFD12 in Burriel-Carranza et al. 2024; see Table 1) [27, 28].

#### Paratypes

IBES7257 (sample code S7257; MorphoBank M849924–M849928), adult female from Wadi As Sareen Nature Reserve, 1,683 m asl, Eastern Hajars (North Oman), 22.13167’N, 58.61889’E, WGS84, collected in May 2011 by S. Carranza, F. Amat and E. Gómez-Díaz (assigned to *P*. *r*. *rupestris* candidate species 16 from Garcia-Porta et al. 2017 and to *Pristurus rupestris* BFD12 in Burriel-Carranza et al. 2024; see Table 1) [27, 28]; MZB 2024–0989 (sample code CN192; MorphoBank M849930–M849932), juvenile from Wadi As Sareen Nature Reserve, 1,682 m asl, Eastern Hajars (North Oman), 22.13182’N, 58.61912’E, WGS84, collected in May 2014 by S. Carranza (assigned to *P*. *r*. *rupestris* candidate species 16 from Garcia-Porta et al. 2017 and to *Pristurus rupestris* BFD12 in Burriel-Carranza et al. 2024; see Table 1) [27, 28].

#### Other material examined

So far, this new species is only known from three specimens (holotype ZFMK 104095 (sample code CN3704); paratypes: MZB 2024–0989 (sample code CN192) and IBES7257 (sample code S7257).

#### Diagnosis

*Pristurus assareen* **sp. nov.** corresponds to a genetically highly distinct lineage from the *Pristurus rupestris* species complex *sensu* Garcia-Porta et al. (2017) [27] inhabiting a secluded and small region of the Eastern Hajar Mountains, North Oman. It is sister taxon to *P*. *rupestris* sensu stricto and constitutes the youngest divergence within the species complex about 3.7 mya (95% HPD: 3.0–4.5 mya; Fig 5). This small species of *Pristurus* is only known from a single locality and is characterized by a combination of the following characters: (1) a maximum recorded SVL of 23.96 mm; (2) 6–7 upper labial scales; (3) 3–4 lower labial scales; (4) 3–5 small postmental scales; (5) absence of mid-dorsal enlarged scales (crest) on the body; (6) laterally compressed tail usually with conspicuous dorsal and smaller ventral enlarged scales (crest) in males, and less conspicuous (sometimes absent) in females; (7) one unique mutation in alignment *rag1*: A instead of C in position 200; (8) one unique mutation in alignment *rag2*: A instead og G in position 325 (see S1 Appendix).

#### Etymology

The species epithet ‘*assareen*’ is a noun in apposition that refers to the geographic distribution range of the species, so far only known from a single locality in Wadi As Sareen Nature Reserve, Eastern Hajar Mountains, Oman.

#### Morphological, genetic and phylogeographic remarks

*Pristurus assareen* **sp. nov.** is morphologically cryptic from the other species of the *Pristurus rupestris* species complex. Although no diagnostic characters have been found, *P*. *assareen* **sp. nov.** tends to have a more robust body, similar to *P*. *ali* **sp. nov.**, with relatively shorter brachia and wider scapular girdles than *P*. *rupestris* sensu stricto, *P*. *omanensis* **sp. nov.** and *P*. *feulneri* **sp. nov.**. This new species can be geographically separated from *P*. *omanensis* **sp. nov.**, *P*. *ali* **sp. nov.** and *P*. *feulneri* **sp. nov.** by its distribution: *P*. *assareen* **sp. nov.** is restricted to a single locality in Wadi As Sareen Nature Reserve (Eastern Hajars), at an elevation of 1,680 m asl, where none of the aforementioned species co-occur. However, *P*. *assareen* **sp. nov.** is found syntopically with *P*. *rupestris* sensu stricto, thus the differentiation between these two species relies on molecular methods. Although there are only morphological data from two adult specimens in the multivariate analyses, it seems that *P*. *assareen* **sp. nov.** is morphologically more similar to *P*. *ali* **sp. nov.** than to any other *Pristurus* species within the species complex, sharing the ‘robust’ morphotype described in Garcia-Porta et al. (2017) [27] with the latter (Fig 3A and S2–S4 Tables).

All mitochondrial (Fig 4A), nuclear loci (Fig 4B), and genome-wide SNP analyses (Figs 3A, 3B, 5, S1 and S2) are fully concordant in the differentiation of *P*. *assareen* **sp. nov.** as a distinct species of its sister clade, *P*. *rupestris* sensu stricto. *Pristurus assareen* **sp. nov.** is monophyletic in both mitochondrial (Fig 4A) and nuclear phylogenetic analyses (Figs 5 and S1). In all phylogenetic reconstructions, it forms a clade together with *P*. *rupestris* sensu stricto representing the youngest split within the species complex, originating about 3.7 mya (Fig 5). The results of the nuclear networks for the *cmos*, *mc1r*, *rag1 and rag2* loci shown in Fig 4B, indicate that *P*. *assareen* **sp. nov.** has some private alleles for each of these genes (not shared with any other species included in the present analysis). Although occurring in syntopy, no signs of admixture were detected between *P*. *assareen* **sp. nov.** and *P*. *rupestris* sensu stricto lineage 3 from Garcia-Porta et al. (2017) (*P*. *rupestris* BFD2 in Burriel-Carranza et al. 2024) [27, 28], and genetic and population genomic analyses show a great level of genetic differentiation between *P*. *assareen* **sp. nov.** and all other species described herein, including *P*. *rupestris* sensu stricto (uncorrected *p*-distance for *12S* gene ranges from 10–23.7%; F_ST_ values range from 0.52–0.87; S5 and S6 Tables respectively). Additionally, although some intraspecific pairwise F_ST_ values within *P*. *feulneri* **sp. nov.** are similar to F_ST_ values between *P*. *assareen* **sp. nov.** and *P*. *rupestris* sensu stricto lineage 3 from Garcia-Porta et al. (2017) [27], when incorporating data on genetic distance (d_xy_), *P*. *assareen* **sp. nov.** *vs P*. *rupestris* sensu stricto seems to be placed within the interspecific diversity cluster (colored dots in Fig 6). In addition, it is important to bear in mind that *P*. *assareen* **sp. nov.** and *P*. *rupestris* sensu stricto coexist syntopically at the only known locality for *P*. *assareen* **sp. nov.** in Wadi As Sareen Nature Reserve. In fact, *P*. *rupestris* sensu stricto seems much more abundant at this locality (S.C. pers. observ.).

#### Description of the holotype

ZFMK 104095 (sample code CN3704) (Fig 22). Adult male. Incomplete specimen lacking the entire right hindlimb, parts of the cloacal fissure, and tail. Data on nine morphometric and two pholidotic variables (see Materials and Methods) are provided in S1 Table. SVL 22.3 mm (incomplete due to parts of the cloaca damaged), head length 30.3% of SVL, and head width 59% of head length. Moderately built, round body, habitus comparable to *P*. *rupestris* sensu stricto; limbs and tail not especially slender. Head robust and does not rise very steeply in profile, snout slightly blunt. Nostril separated from the rostral and situated between three scales: a wide supranasal extending to the midline margin, a small upper postnasal and a large lower postnasal extending laterally along the lower edge. Supranasals are separated in two scales. Scales on snout granular, polygonal and slightly pointed upwards. About eight scales in a straight line from the lower postnasal to the anterior edge of the orbit. Scales on snout, anterior to eyes, are larger than those on top of head. Around 20 scales across mid-orbital region. Palpebral fold edged anteriorly with large scales and not edged with pointed (ciliate) scales. Ear opening rounded. Upper labials (right/left) 7/7. Lower labials (right/left) 4/4, the fourth decreasing in size from the three large anterior scales. Four enlarged scales running backwards along the proximal borders of the third lower labials to end at about the level of the angle of the mouth. Mental large, rounded, truncated posteriorly by six gular scales. Gulars small, rather pointed posteriorly, becoming slightly imbricate on throat. Dorsal scales on body small, polygonal and homogeneous, slightly pointed posteriorly. Ventrals flat and distinctly imbricate. Scales on dorsal and anterior surface of the forelimbs slightly larger than dorsal body scales, flat and imbricate, fairly pointed. Scales beneath the upper forelimbs granular and smaller than ventral body scales; those beneath lower forelimbs large, flat, imbricate and polygonal, larger than ventral body scales. Claws relatively shallow and weakly recurved. Scales on dorsal and anterior surface of hindlimbs large, flat and imbricate, fairly pointed. Scales beneath the upper hindlimbs granular and smaller than ventral body scales, those beneath lower hindlimbs large, flat, imbricate and polygonal, larger than ventral body scales. Tail absent.

Coloration in alcohol grayish dorsally; blurred dorsal midline dark grayish. Dorsally and both left and right of the midline irregularly shaped reddish blotches present, surrounded by local grayish patterns slightly darker than the background coloration. Dorsolaterally thin pale reddish lines that dissolve into discontinuous lines posteriorly, and also ventrolaterally to the flanks up to spots comprised by individually colored scales. Blurred whitish spots distributed along the flanks. Head is the same color as the body with a broad dark brownish stripe running from the nostril through the eye and then dorsolaterally until above the ear opening. Single large dark blotches are located from beneath the angle of the mouth to the entire gular region reaching posteriorly to the insertion of the forelimbs. Most of them being large and prominent blotches on the throat becoming more blurred posteriorly. Ventral background coloration creamish-whitish. Limbs dark pale ventrally and brownish with blurred whitish spots dorsally, toes not banded.

The figured μ-CT-scanned specimen (Fig 22B) has a maximal skull length of 7.3 mm, maximal height of 3.0 mm and maximal width of 4.7 mm. 23 presacral vertebrae (vertebral column incomplete). Noteworthy minor qualitative cranial osteological differences (Fig 22C) to the other holotype specimens of the *Pristurus rupestris* species complex are: Vomer posteriorly only slightly rounded and bordered by a hoof-shaped opening; osseous naris edge smooth and slightly rounded; both dentaries fractured; premaxilla and nasal moderately steep to frontal region.

#### Variation

Data on nine morphometric and two pholidotic variables (see Materials and Methods) for the two paratypes (see above) are provided in S1 Table. As of today, only three specimens of *P*. *assareen* **sp. nov.** have been found: one male, one female and one juvenile specimen. Therefore, knowledge on the variation of this species is limited to the material available. Both paratypes have broken or missing tails. Paratype IBES7257 (sample code S7257) has the throat sliced. Dorsal coloration similar for both paratypes, with conspicuous dorsal midline (especially in the female specimen), colored pale-beige. Background coloration is grayish-brownish with reddish spots distributed in longitudinal lines separated by dorsal bright spots. Multiple gular blotches present in both specimens. Ventral pattern uniform pale beige-whitish, with reddish spots distributed ventrolaterally only (Fig 23).

#### Distribution

**Fig 25:**
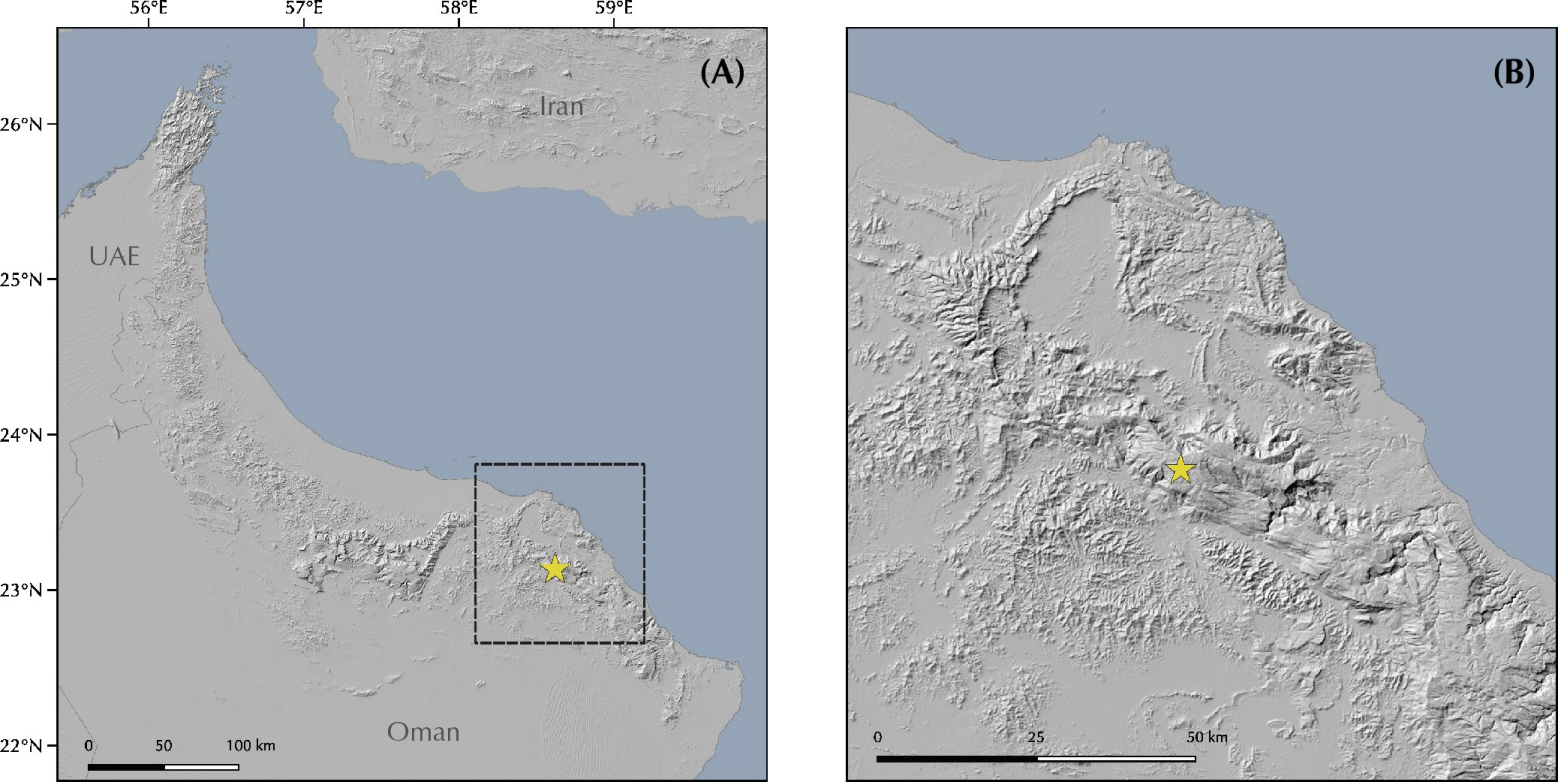
Distribution of *P*. *assareen* **sp. nov.** in Arabia including **(A)** the single locality this species is known from within the Hajar Mountains range context; **(B)** a close-up of the Wadi As Sareen where the species is endemic to.

*Pristurus assareen* **sp. nov.** is only known from a single locality in the Wadi As Sareen Nature Reserve, Eastern Hajars, North Oman (Figs 24 and 25). Despite extensive surveys across surrounding wadis and the whole Hajar Mountain range, this secluded taxon has never been found anywhere else (Fig 25). It occurs at 1,680 m asl and is completely sympatric and syntopic with *P*. *rupestris* sensu stricto and more specifically with lineage 3 *sensu* Garcia-Porta et al. (2017) [27]. Therefore, this new species is endemic to Oman and, so far, constitutes the species of vertebrate with the smallest distribution range in Arabia.

#### Habits

Very little data exists on this very secretive species of the *Pristurus rupestris* species complex. The only three known specimens were found during the day and at sunset in a rocky plateau in complete syntopy with *Pristurus rupestris* sensu stricto (Fig 24). During one of the expeditions to the Hajar Mountains carried out to study the *Pristurus rupestris* species complex, only two specimens out of a total of 25 specimens collected at the only known locality of *Pristurus assareen* **sp. nov.** at Wadi As Sareen Nature Reserve belonged to this species, while the remaining 23 specimens belonged to *Pristurus rupestris* sensu stricto (lineage 3 sensu Garcia-Porta et al. 2017) [27].

#### Conservation

This species is currently considered to be endemic to Wadi As Sareen Nature Reserve in the Eastern Hajar Mountains, Oman. It occurs in a single locality with an area of occupancy (AOO) of 4 km^2^, and is considered to occur in one location based on the inferred threat from competition with a lowland lineage of *Pristurus rupestris* sensu stricto (lineage 3 from Garcia-Porta et al. 2017) [27]. The species should be assessed as Critically Endangered (CR B2ab(ii,v)) on the basis of restricted AOO, and continuing decline in the AOO and in the number of mature individuals.

## Discussion

In the present study, we examined a species radiation that dates back more than 10 mya, encompassing the smallest and most widespread gecko species inhabiting the Hajar Mountains of southeastern Arabia. Results support the existence of four previously unrecognized cryptic species of the genus *Pristurus*, with one species (together with *P*. *rupestris* sensu stricto) being widely distributed across the Hajar Mountains and spanning throughout the entire elevation gradient of the mountain range (*P*. *feulneri* **sp. nov.**; Table 1 and Fig 21), two species displaying restricted ranges endemic to the Eastern Hajars and only occurring from mid to high elevations (*P*. *ali* **sp. nov.** and *P*. *assareen* **sp. nov.**; Table 1 and Figs 17 and 25 respectively), and one lowland dwelling *Pristurus* endemic to Oman, distributed around the Central and Eastern Hajars (*P*. *omanensis* **sp. nov.**; Table 1 and Fig 13).

Despite the lack of morphological diagnostic characters, significant quantitative morphological differences were found between the range-restricted, high elevation *Pristurus* species and all other relatives, the former broadly fitting to a more robust morphotype with shorter limbs and wider heads (Fig 3A and S2–S4 Tables; [27]). However, it is important to note that while these differences exist between some specimens, there is a substantial degree of overlap in shape among species, resulting in considerable variation in morphological traits that effectively render visual species differentiation impossible in the field. In fact, rather than displaying a wide spectrum of shape variation, the species within this complex appear to be morphologically constrained when compared to other *Pristurus* species, comprising less variation than what could be anticipated by their deep evolutionary history [79, 80]. A similar situation also occurred between the *Pristurus rupestris* species complex and the only recently resurrected *Pristurus guweirensis* [18, 22], two non-related and molecularly highly divergent species recognized for more than 140 years as the same species due to their morphological similarity. The conserved morphology seen in the *Pristurus rupestris* species complex is not uncommon in Arabian geckos. A similar case of morphological stasis can be found in the geckos of the genus *Hemidactylus* from southwest Arabia [81], geckos of the genus *Ptyodactylus* [13, 49, 82], and agamids of the genus *Pseudotrapelus* [83]. In those cases, morphologically cryptic species complexes have been found, mostly speciating in allopatry in mountain ranges and with only a few areas where two species coexist in sympatry.

Such conserved morphology in the *P*. *rupestris* species complex might have been driven by the effects of stabilising selection acting through different factors. All *Pristurus* species described in this study (including *P*. *rupestris* sensu stricto) occupy a similar microhabitat, predominantly rocky substrates such as rocks or boulders, tree trunks, or human-made structures [26], hence stabilising selection on adaptive traits related to the exploitation of this particular structural niche might be occurring. Such factor is concordant with a scenario of allopatric speciation, in which a lack of resource partitioning could have driven each species to maintain a similar microhabitat (i.e. presenting extensive overlap in their body size and shape) [13, 84]. Although some species are currently widely distributed throughout the mountain range overlapping in distribution range with other species in the complex, not more than two species coexist at the same locality (S7 Table), and biogeographic reconstructions suggest that speciation between these five species could have occurred in allopatry (Burriel-Carranza et al. 2024: S18 Fig) [28] with subsequent dispersal. Additionally, strong environmental conditions can also favour a constrained morphological spectrum. The extreme environmental conditions of the Hajar Mountains (considered a desert mountain range; [31]), might have also impelled a morphological stasis similar to the case of several cryptic species in the Arctic tundra or deep-sea environments [85].

However, although no apparent phenotypic traits were detected, some pre- or postzygotic reproductive isolation mechanisms must exist between these species since no signs of gene flow were detected among them despite the extensive sampling including several syntopic localities (Figs 3B, 3C and S38–S43 in Burriel-Carranza et al. 2024) [28]. As diurnal species with complex visual signaling [19, 23, 25, 26], these *Pristurus* species might rely on species-specific behaviour to recognise their conspecific relatives. However, other isolation mechanisms such as postzygotic reproductive isolation cannot be ruled out, and future studies should be conducted to better understand the evolution of reproductive isolation in this species complex.

Molecular analyses unequivocally support the existence of at least four new cryptic species of *Pristurus* geckos in the Hajar Mountains, with divergence time estimates ranging from 10.8 to almost four mya (Fig 5). These results highlight the importance of the Hajar Mountains as biodiversity reservoirs, showcasing the oldest case of cryptic speciation in this Arabian hotspot of reptile diversity. The deep mitochondrial and nuclear divergences observed between these species are not only remarkable when compared to other sibling species in the mountain range [13, 14], but are also greater than between several sister species of the genus *Pristurus* [72, 86]. For instance, genetic distances on the mitochondrial *12S* marker are higher across all species of the *Pristurus rupestris* species complex (9.4–24.6%; S5 Table) than between the recently described *P*. *masirahensis* and its continental sister taxon *P*. *minimus* (7.1%) [72].

Intraspecific diversity within the recognized species fluctuates from almost null in *P*. *assareen* **sp. nov.**, to relatively shallow in *P*. *ali* **sp. nov.** and *P*. *omanensis* **sp. nov.**, and to remarkably diverse in *P*. *rupestris* sensu stricto and *P*. *feulneri* **sp. nov.** (Figs 3C, 4A, 5, S1 and S2 and Tables S5 and S6). Multispecies Coalescent (MSC) species delimitation methods, with both Sanger sequencing [27] and next generation sequencing (Figs 5 and S1; [28]) data, generally supported a greater number of species than the ones formally described herein, especially within the latter two species. We proceeded with caution and refrained from further splitting more taxa as intraspecific mito-nuclear discordances (Figs 4A and S1), as well as incomplete lineage sorting (Fig 5) were observed within the recognized species. Moreover, incongruent results were obtained when evaluating species status with an heuristic criterion (the *gdi*) since most of the undescribed diversity fell within the ambiguous zone between 0.2 and 0.7 (Figs 5 and S2). The only exception was the exceptionally diverse population of *P*. *feulneri* **sp. nov.** population 15 sensu Garcia-Porta et al. (2017) [27]. Isolated in the Eastern Hajars, this population diverged from the rest of its conspecific relatives circa 3 mya (Fig 5). We decided not to recognize it as distinct at species level because its reproductive isolation from the other *P*. *feulneri* **sp. nov.** could not be definitively determined (i.e. in contrast to all other described species; S7 Table). This was supported by the lack of reciprocal monophyly between *P*. *feulneri* **sp. nov.** population 15 and its other conspecific populations in the mitochondrial phylogenetic reconstruction (Fig 4A), and by the results of pairwise F_ST_ vs d_xy_ comparisons across the *Pristurus rupestris* species complex populations (following a recent species delimitation approach; [39]). In the latter analysis, *P*. *feulneri* **sp. nov.** population 15 clustered closer to what could be identified as an intraspecific variability cluster, showing a clear gap with the pairwise comparisons between *P*. *assareen* **sp. nov.** and *P*. *rupestris* sensu stricto populations (Fig 6).

*Pristurus rupestris* sensu lato (i.e. including the four new species described herein) is currently considered as Least Concerned by the IUCN Red List of threatened species, and indeed, morphologically typical specimens can be found in great numbers across the Hajar Mountains. However, here we found that two narrow-ranged Eastern Hajars’ endemics (*P*. *ali* **sp. nov.** and *P*. *assareen* **sp. nov.**) were being concealed by the most abundant species in the species complex (*P*. *feulneri* **sp. nov.**, *P*. *omanensis* **sp. nov.** and *P*. *rupestris* sensu stricto). Moreover, *P*. *assareen* **sp. nov.** is a microendemic species only known from a single locality in Wadi as Sareen Nature Reserve, Eastern Hajars, representing one of the smallest-ranged extant Arabian reptiles [87]. Not only *P*. *assareen* **sp. nov.** is more prone to extinction by the intrinsic threats of being a microendemic species [87], but it also occurs in syntopy with its sister species, *P*. *rupestris* sensu stricto, which is quite abundant at *P*. *assareen* **sp. nov.**’s type locality and might pose an additional threat through resource competition. Therefore, *P*. *assareen* **sp. nov.** is in urgent need of protection and conservation assessment. Together with the description of the cryptic species *Ptyodactylus ruusaljibalicus* [13] and the taxonomic reevaluation of the Critically Endangered Leaf-toed Gecko, *Asaccus caudivolvulus* [11], this study exemplifies what potential impacts of the lack of taxonomic knowledge (the Linnean shortfall; [88]) may have on conservation planning and the preservation of biodiversity.

## Supporting information

S1 Fig Maximum likelihood phylogenomic reconstruction of the *Pristurus rupestris* species complex species. This analysis was inferred with 163 *Pristurus* specimens from the *P*. *rupestris* species complex, 10 *P*. *flavipunctatus* used as an outgroup to root the tree, and a concatenated dataset of 137,800 bp and 20,845 SNPs (*dataset3*; Table 1). Numbers in gray circles correspond to genetic lineages recovered as putative species in Garcia-Porta et al. (2017). Black dots at nodes: bootstrap support (bs) > 0.95; White at nodes dots: 0.75 < bs > 0.95. To the right, vertical colored bars indicate the number of lineages recovered as distinct species with different species delimitation methods from Burriel-Carranza et al. (2024; BFD) and the present study (BPP and GDI). BFD: Bayes Factor Delimitation* (*with genomic data); BPP: BPP A10 species delimitation analysis; GDI: Genealogical divergence index (*gdi*).
(PDF)

S2 Fig Comparisons of *Pristurus rupestris* species complex populations and species using two measures of genetic divergence. **(A)** Guide tree used for the multispecies coalescent estimation of population sizes (theta) and divergence times (tau) in BPP. The tree was obtained in the present study (see Fig 4). Boxplots for *gdi* **(B)** and coalescent units **(C)** calculated using the posterior probability distributions for theta and tau estimated in BPP.
(PDF)

S3 Fig *Pristurus omanensis* sp. nov. variation. **(A)** Dorsal and **(B)** ventral view of *Pristurus omanensis* **sp. nov.** specimens showing color variation. All specimens correspond to specimens from the Central Hajars assigned to lineage BFD6 (Burriel-Carranza et al. 2024) and lineage 9 *sensu* Garcia-Porta et al. (2017; see Table 1). Further variation in specimens of *P*. *omanensis* **sp. nov.** lineage BFD5 *sensu* Burriel-Carranza et al. (2024) is shown in Fig 10.
(PDF)

S4 Fig *Pristurus ali* sp. nov. variation. **(A)** Dorsal and **(B)** ventral view of *Pristurus ali* **sp. nov.** specimens showing color variation. All specimens correspond to specimens from the Eastern Hajars assigned to genetic lineage BFD4 (Burriel-Carranza et al. 2024; *P*. *r*. *rupestris* candidate species 6 in Garcia-Porta et al., 2017; see Table 1). Further variation in specimens of *P*. *ali* **sp. nov.** assigned to lineage 7 (*P*. *r*. *rupestris* candidate species 7 in Garcia-Porta et al., 2017) is shown in Fig 14.
(PDF)

S5 Fig *Pristurus feulneri* sp. nov. variation 1. **(A)** Dorsal and **(B)** ventral view of *Pristurus feulneri* **sp. nov.** specimens showing color variation. All specimens correspond to specimens assigned to lineage BFD7 (Burriel-Carranza et al. 2024; *P*. *r*. *rupestris* candidate species 10.11 in Garcia-Porta et al., 2017; see Table 1). Further variation in specimens of *P*. *feulneri* **sp. nov.** genomic lineages BFD8–11 (Burriel-Carranza et al. 2024; *P*. *r*. *rupestris* candidate species 12–15 in Garcia-Porta et al., 2017) are shown in Fig 18 and supplementary figures below.
(PDF)

S6 Fig *Pristurus feulneri* sp. nov. variation 2. (A) Dorsal and (B) ventral view of *Pristurus feulneri* sp. nov. specimens showing color variation. All specimens correspond to specimens assigned to lineage BFD8 (Burriel-Carranza et al. 2024; *P. r. rupestris* candidate species 12 in Garcia-Porta et al., 2017; see Table 1) from the Semail gap, in the Central Hajars. Further variation in specimens of *P. feulneri* sp. nov. lineages BFD7,9–11 (Burriel-Carranza et al. 2024; *P. r. rupestris* candidate species 10.11,13–15 in Garcia-Porta et al., 2017), and genetic are shown in Figs 18, S5, S7 and S8.
(PDF)

S7 Fig *Pristurus feulneri* sp. nov. variation 3. (A) Dorsal and (B) ventral view of *Pristurus feulneri* sp. nov. specimens showing color variation. All specimens correspond to specimens assigned to lineage BFD10 (Burriel-Carranza et al. 2024) and genetic lineages 14 (Garcia-Porta et al., 2017; three specimens to the left) and 17 (present study; three specimens to the right) (see Table 1) from the Jabal Akhdar, in the Central Hajars. Further variation in specimens of *P. feulneri* sp. nov. lineages BFD7–9,1 (Burriel-Carranza et al. 2024; *P. r. rupestris* candidate species 10.11–15 in Garcia-Porta et al., 2017; see Table 1), are shown in Figs 18 and S5, S6 and S8.
(PDF)

S8 Fig *Pristurus feulneri* sp. nov. variation 4. **(A)** Dorsal and **(B)** ventral view of *Pristurus feulneri* **sp. nov.** specimens showing color variation. All specimens correspond to specimens assigned to lineage BFD11 (Burriel-Carranza et al. 2024; *P*. *r*. *rupestris* candidate species 15 in Garcia-Porta et al., 2017; see Table 1) from the Eastern Hajars. Further variation in specimens of *P*. *feulneri* **sp. nov.** genomic lineages BFD7–10 (Burriel-Carranza et al. 2024; *P*. *r*. *rupestris* candidate species 10.11–14 in Garcia-Porta et al., 2017; see Table 1), are shown in Figs 18, S5, S6 and S7.
(PDF)

S1 Table Table on nine morphometric and two pholidotic variables for all holotypes and paratypes of the four new species of *Pristurus* described herein. Holotypes are highlighted in bold.
(XLSX)

S2 Table Loadings and variance explained by each of the first ten components retained from the PCA performed on shape residuals of *Pristurus rupestris* species complex specimens. Abbreviations are as follows: (HL) Head length; (HW) Head width; (HH) Head hight; (ASG) Body width at the scapular girdle; (APG) Body width at the pelvic girdle; (TrL) Trunk length; (LHu) Humerus length; (LUn) Ulna length; (LFe) Femur length; (LTb) Tibia length.
(XLSX)

S3 Table Pairwise procrustes ANOVA on *Pristurus* shape residuals inferred from 10 independent morphological measurements. Significant values (p < 0.05) are shown in bold.
(XLSX)

S4 Table Pairwise procrustes ANOVA’s p-values on the individual morphological variables. Significant values (p < 0.05) are shown in bold. Abbreviations of each variable as in S1 Table.
(XLSX)

S5 Table Uncorrected p-distances for the 12S mitochondrial gene fragment among all five species conforming the *Pristurus rupestris* species complex. Values in the diagonal show intraspecific diversity in the 12S mitochondrial gene fragment.
(XLSX)

S6 Table Standardized F_ST_ values (upper corner) and p-values (bottom corner) across all species within the *Pristurus rupestris* species complex. P. = *Pristurus*.
(XLSX)

S7 Table Sympatry analysis of all *Pristurus* species in the *Pristurus rupestris* species complex. Each row represents a contact locality where two species have been found at less than 50 m from each other. *Species1* and *Species2* columns refer to the first and second species mentioned at column *Species* in sympatry respectively.
(XLSX)

S1 Appendix Alignment of phased haplotypes for the molecular diagnostic characters based on the *mc1r*, *cmos*, *rag1* and *rag2*. (ZIP)
