# Supplementary material for: Cryptic speciation in arid mountains: An integrative revision of the Pristurus rupestris species complex (Squamata, Sphaerodactylidae) from Arabia based on morphological, genetic and genomic data, with the description of four new species
Source: PLoS One. 2025 Feb 24;20(2):e0315000. doi: 10.1371/journal.pone.0315000 (PMC11849857; doi:10.1371/journal.pone.0315000)
Supplement: S1 Fig — This analysis was inferred with 163 Pristurus specimens from the P. rupestris species complex, 10 P. flavipunctatus used as an outgroup to root the tree, and a concatenated dataset of 137,800 bp and 20,845 SNPs (dataset3; Table 1). Numbers in gray circles correspond to genetic lineages recovered as putative species in Garcia-Porta et al. (2017). Black dots at nodes: bootstrap support (bs) > 0.95; White at nodes dots: 0.75 < bs > 0.95. To the right, vertical colored bars indicate the number of lineages recovered as distinct species with different species delimitation methods from Burriel-Carranza et al. (2024; BFD) and the present study (BPP and GDI). BFD: Bayes Factor Delimitation* (*with genomic data); BPP: BPP A10 species delimitation analysis; GDI: Genealogical divergence index (gdi). (PDF) [file pone.0315000.s001.pdf]

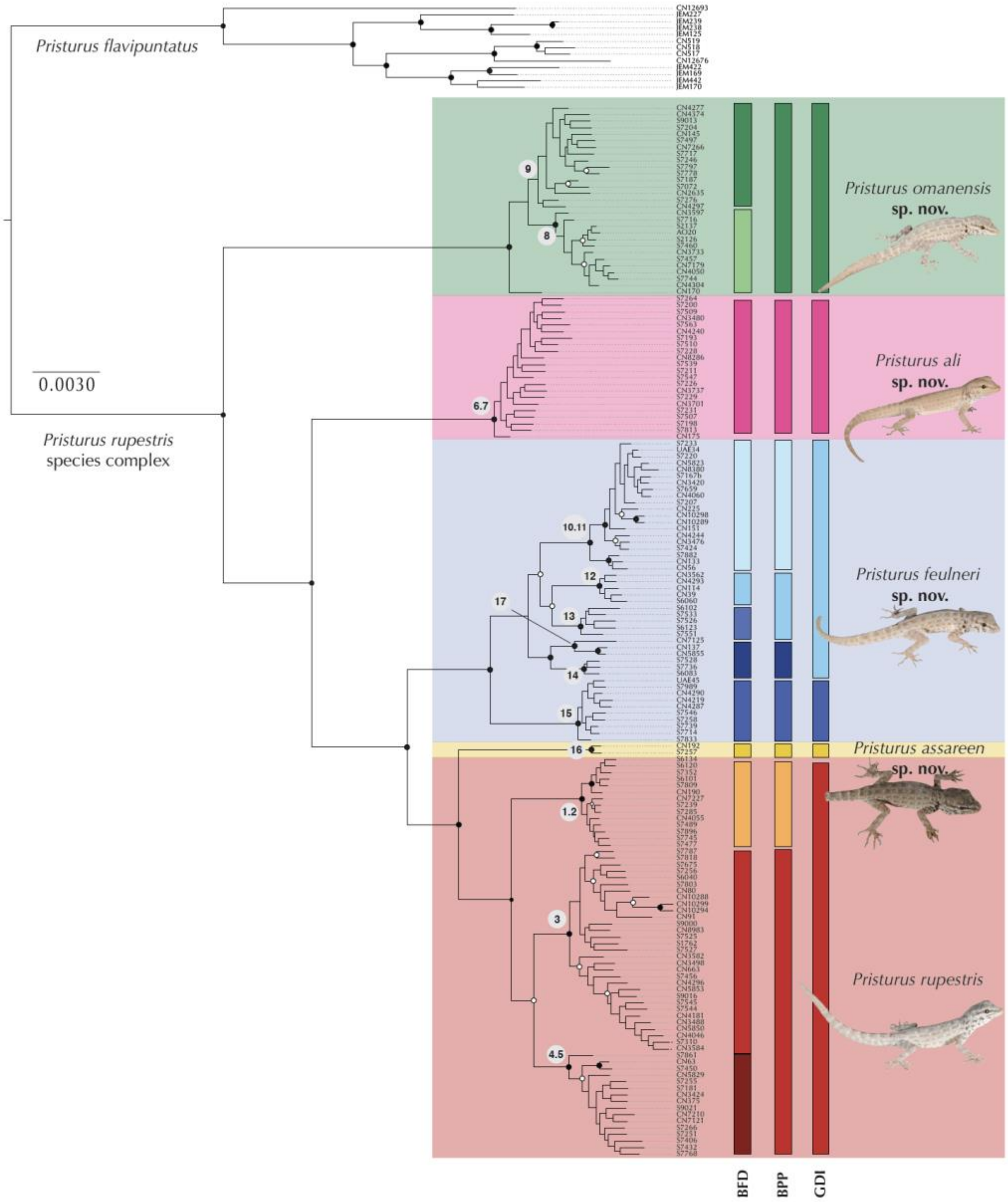

Figure S1. Maximum likelihood phylogenomic reconstruction of the *Pristurus rupestris* species complex. This analysis was inferred with 163 *Pristurus* specimens from the *P. rupestris* species complex, 10 *P. flavipunctatus* used as outgroup to root the tree, and a concatenated dataset of 137,800 bp and 20,845 SNPs (*dataset3*; Table 1). Numbers in grey circles correspond to genetic lineages recovered as putative species in Garcia-Porta et al. (2017). Black dots at nodes: bootstrap support (bs) > 0.95; White at nodes dots: 0.75 < bs > 0.95. To the right, vertical colored bars indicate the number of lineages recovered as distinct species with different species delimitation methods from Burriel-Csrranza et al. (2023b; BFD) and the present study (BPP and GDI). BFD: Bayes Factor Delimitation* (*with genomic data); BPP: BPP A10 species delimitation analysis; GDI: Genealogical divergence index (*gdi*).
