# Supplementary material for: Cryptic speciation in arid mountains: An integrative revision of the Pristurus rupestris species complex (Squamata, Sphaerodactylidae) from Arabia based on morphological, genetic and genomic data, with the description of four new species
Source: PLoS One. 2025 Feb 24;20(2):e0315000. doi: 10.1371/journal.pone.0315000 (PMC11849857; doi:10.1371/journal.pone.0315000)
Supplement: S2 Fig — (A) Guide tree used for the multispecies coalescent estimation of population sizes (theta) and divergence times (tau) in BPP. The tree was obtained in the present study (see Fig 4). Boxplots for gdi (B) and coalescent units (C) calculated using the posterior probability distributions for theta and tau estimated in BPP. (PDF) [file pone.0315000.s002.pdf]

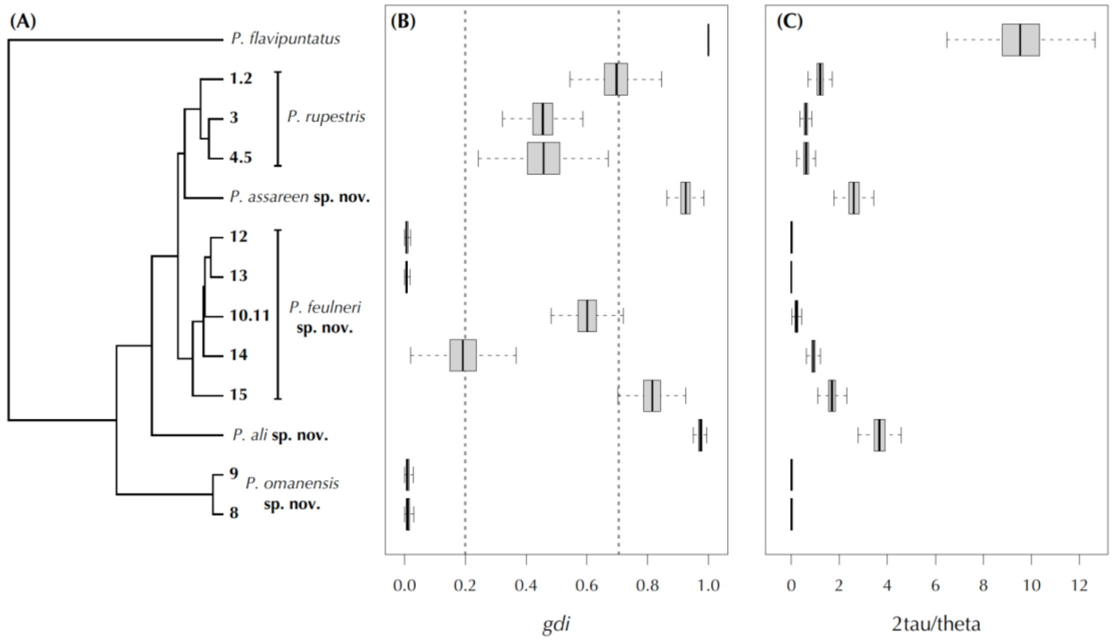

Figure S2. Comparisons of *Pristurus rupestris* species complex populations and species using two measures of genetic divergence. (A) Guide tree used for the multispecies coalescent estimation of population sizes (theta) and divergence times (tau) in BPP. The tree was obtained in the present study (see Figure 4). Boxplots for *gdi* **(B)** and coalescent units **(C)** calculated using the posterior probability distributions for theta and tau estimated in BPP.
