# Supplementary material for: Cryptic speciation in arid mountains: An integrative revision of the Pristurus rupestris species complex (Squamata, Sphaerodactylidae) from Arabia based on morphological, genetic and genomic data, with the description of four new species
Source: PLoS One. 2025 Feb 24;20(2):e0315000. doi: 10.1371/journal.pone.0315000 (PMC11849857; doi:10.1371/journal.pone.0315000)
Supplement: S3 Fig — (A) Dorsal and (B) ventral view of Pristurus omanensis sp. nov. specimens showing color variation. All specimens correspond to specimens from the Central Hajars assigned to lineage BFD6 (Burriel-Carranza et al. 2024) and lineage 9 sensu Garcia-Porta et al. (2017; see Table 1). Further variation in specimens of P. omanensis sp. nov. lineage BFD5 sensu Burriel-Carranza et al. (2024) is shown in Fig 10. (PDF) [file pone.0315000.s003.pdf]

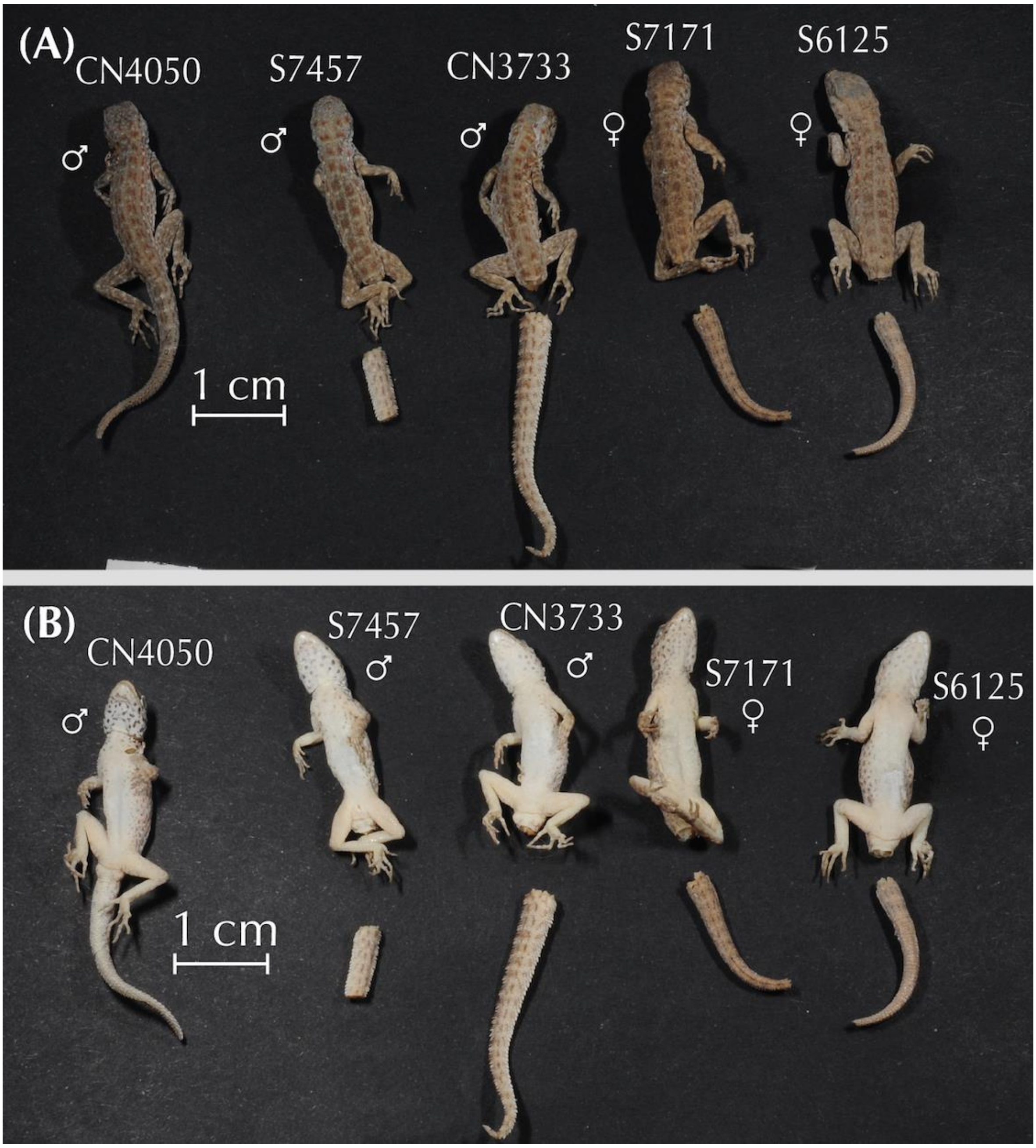


**Figure S3. *Pristurus omanensis* sp. nov. variation. (A)** Dorsal and **(B)** ventral view of *Pristurus omanensis* **sp. nov.** specimens showing color variation. All specimens correspond to specimens from the Central Hajars assigned to genomic lineage BFD6 in Burriel-Carranza et al. (2023b) and genetic lineage 9 in Garcia-Porta et al. (2017) (see Table1). Further variation in specimens of *P. omanensis* **sp. nov.** genomic lineage BFD5 is shown in Figure 10.
