# Supplementary material for: Cryptic speciation in arid mountains: An integrative revision of the Pristurus rupestris species complex (Squamata, Sphaerodactylidae) from Arabia based on morphological, genetic and genomic data, with the description of four new species
Source: PLoS One. 2025 Feb 24;20(2):e0315000. doi: 10.1371/journal.pone.0315000 (PMC11849857; doi:10.1371/journal.pone.0315000)
Supplement: S4 Fig — (A) Dorsal and (B) ventral view of Pristurus ali sp. nov. specimens showing color variation. All specimens correspond to specimens from the Eastern Hajars assigned to genetic lineage BFD4 (Burriel-Carranza et al. 2024; P. r. rupestris candidate species 6 in Garcia-Porta et al., 2017; see Table 1). Further variation in specimens of P. ali sp. nov. assigned to lineage 7 (P. r. rupestris candidate species 7 in Garcia-Porta et al., 2017) is shown in Fig 14. (PDF) [file pone.0315000.s004.pdf]

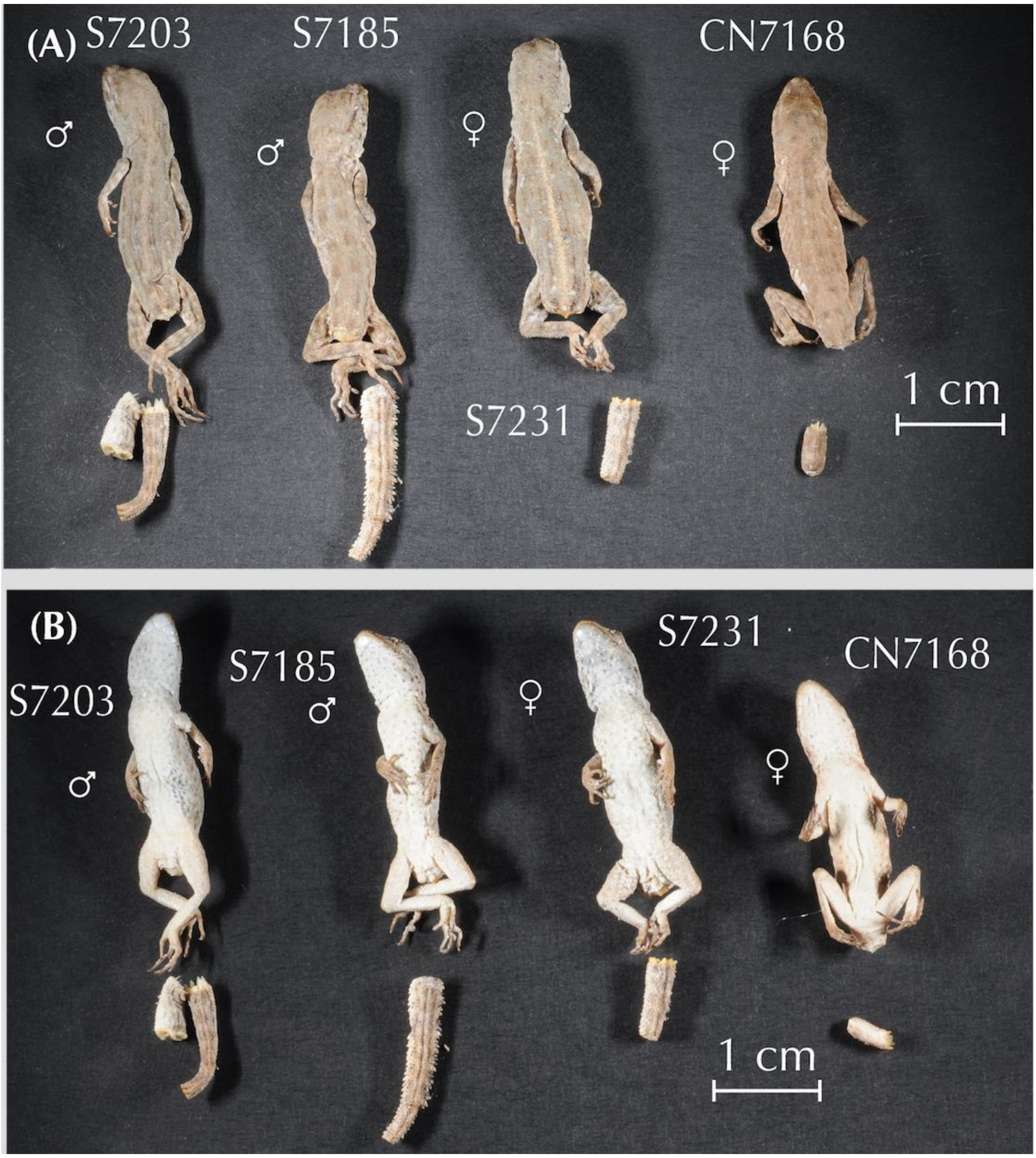

Figure S4. (A) Dorsal and **(B)** ventral view of *Pristurus ali* **sp. nov.** specimens showing color variation. All specimens correspond to specimens from the Eastern Hajars assigned to genomic lineage BFD4 in Burriel-Carranza et al. (2023b) and genetic lineage 6 in Garcia-Porta et al. (2017) (see Table1). Further variation in specimens of *P. ali* **sp. nov.** genomic lineage BFD4, genetic lineage 7 is shown in Figure 14.
