# Supplementary material for: Cryptic speciation in arid mountains: An integrative revision of the Pristurus rupestris species complex (Squamata, Sphaerodactylidae) from Arabia based on morphological, genetic and genomic data, with the description of four new species
Source: PLoS One. 2025 Feb 24;20(2):e0315000. doi: 10.1371/journal.pone.0315000 (PMC11849857; doi:10.1371/journal.pone.0315000)
Supplement: S7 Fig — (A) Dorsal and (B) ventral view of Pristurus feulneri sp. nov. specimens showing color variation. All specimens correspond to specimens assigned to lineage BFD10 (Burriel-Carranza et al. 2024) and genetic lineages 14 (Garcia-Porta et al., 2017; three specimens to the left) and 17 (present study; three specimens to the right) (see Table 1) from the Jabal Akhdar, in the Central Hajars. Further variation in specimens of P. feulneri sp. nov. lineages BFD7–9,1 (Burriel-Carranza et al. 2024; P. r. rupestris candidate species 10.11–15 in Garcia-Porta et al., 2017; see Table 1), are shown in Figs 18 and S5, S6 and S8. (PDF) [file pone.0315000.s007.pdf]

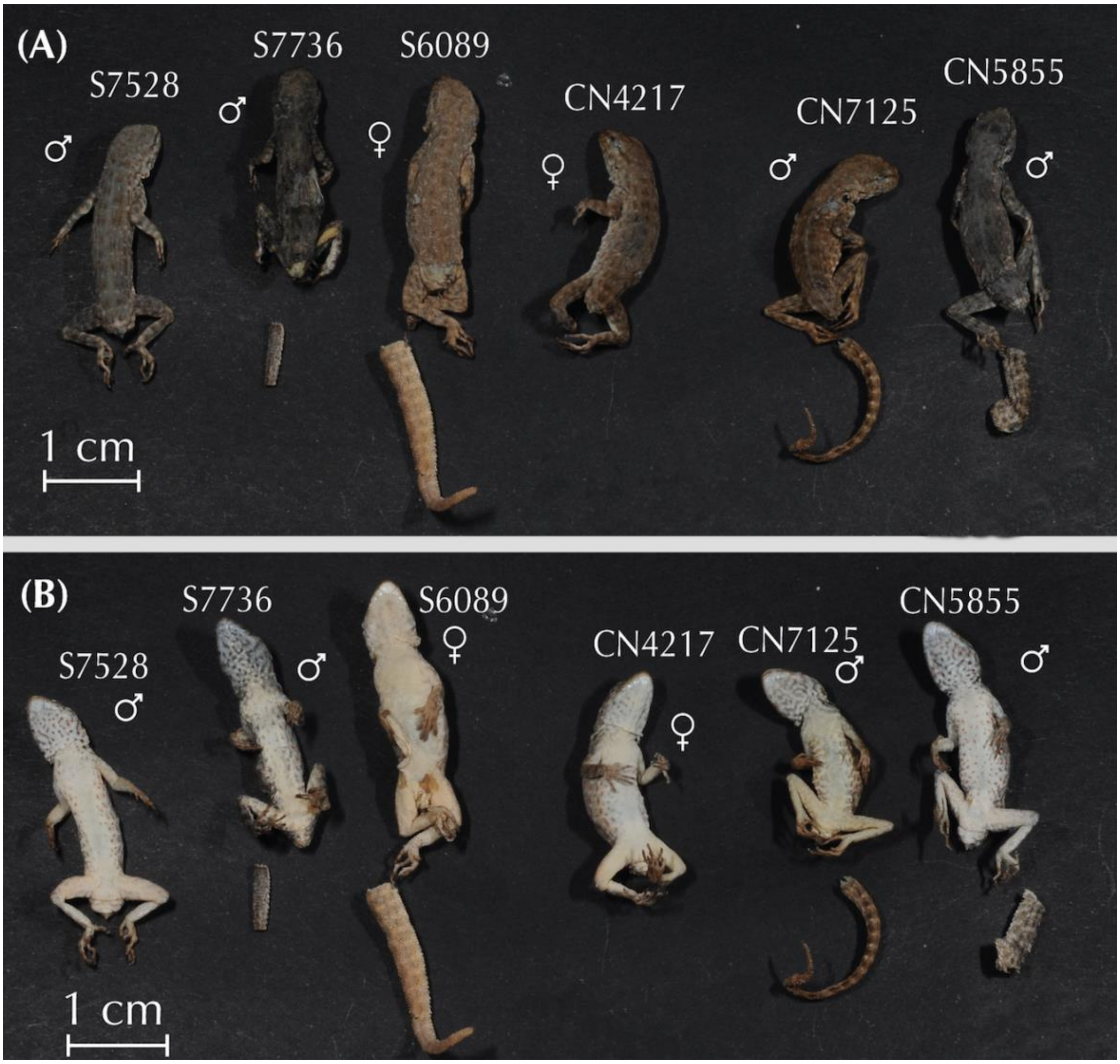

Figure S7. (A) Dorsal and **(B)** ventral view of *Pristurus feulneri* **sp. nov.** specimens showing color variation. All specimens correspond to specimens assigned to genomic lineage BFD10 in Burriel-Carranza et al. (2023b) and genetic lineages 14 in in Garcia-Porta et al. (2017) (three specimens to the left) and 17 (three specimens to the right) (see Table1) from the Jabal Akhdar, in the Central Hajars. Further variation in specimens of *P. feulneri* **sp. nov.** genomic lineages BFD7–9,11, and genetic lineages 10.11–15 is shown in Figures 18, S5, S6 and S8.
