# Supplementary material for: Cryptic speciation in arid mountains: An integrative revision of the Pristurus rupestris species complex (Squamata, Sphaerodactylidae) from Arabia based on morphological, genetic and genomic data, with the description of four new species
Source: PLoS One. 2025 Feb 24;20(2):e0315000. doi: 10.1371/journal.pone.0315000 (PMC11849857; doi:10.1371/journal.pone.0315000)
Supplement: S8 Fig — (A) Dorsal and (B) ventral view of Pristurus feulneri sp. nov. specimens showing color variation. All specimens correspond to specimens assigned to lineage BFD11 (Burriel-Carranza et al. 2024; P. r. rupestris candidate species 15 in Garcia-Porta et al., 2017; see Table 1) from the Eastern Hajars. Further variation in specimens of P. feulneri sp. nov. genomic lineages BFD7–10 (Burriel-Carranza et al. 2024; P. r. rupestris candidate species 10.11–14 in Garcia-Porta et al., 2017; see Table 1), are shown in Figs 18, S5, S6 and S7. (PDF) [file pone.0315000.s008.pdf]

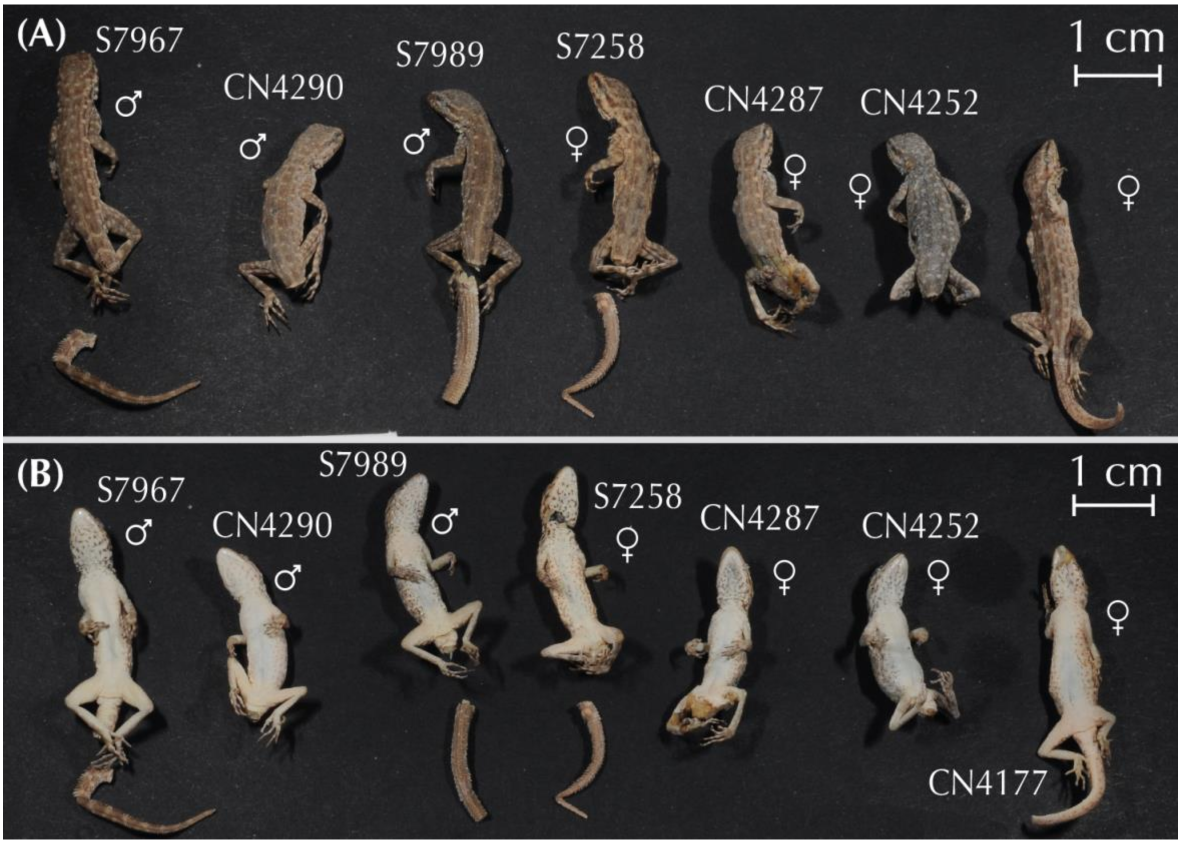

Figure S8. (A) Dorsal and **(B)** ventral view of *Pristurus feulneri* **sp. nov.** specimens showing color variation. All specimens correspond to specimens assigned to genomic lineage BFD11 in Burriel-Carranza et al. (2023b) and genetic lineage 15 in Garcia-Porta et al. (2017) (see Table1) from the Eastern Hajars. Further variation in specimens of *P. feulneri* **sp. nov.** genomic lineages BFD7–10, and genetic lineages 10.11–14,17 is shown in Figures 18, S5, S6 and S7.
